# Circular Photocaged mRNA for Light‐induced Late‐stage Activation of Translation

**DOI:** 10.1002/anie.8731921

**Published:** 2026-07-27

**Authors:** Bayram Terzi, Greta Charlotte Dahm, Kristoffer Heintz, Martin Sumser, Yasuaki Kimura, Hiroshi Abe, Andrea Rentmeister

**Affiliations:** ^1^ Department of Chemistry Ludwig‐Maximilians‐Universität (LMU) München 81377 München Germany; ^2^ Cluster For Nucleic Acid Sciences and Technologies – NUCLEATE; ^3^ Department of Chemistry Graduate School of Science Nagoya University Nagoya Aichi 464–8602 Japan

**Keywords:** 5′ cap, circular RNA, mRNA, optochemical biology, translation

## Abstract

mRNA is a new medical modality as vaccine and raises hopes to become a protein replacement agent. Despite notable advances in manufacturing, stabilization and modification, its short half‐life remains a major limitation. Circular RNA bears potential to solve this issue, as degradation of mRNA proceeds primarily from the 5′ and 3′ ends. However, circular RNAs normally lack a 5′ cap and are less efficiently translated. We present a strategy to circularize mRNA covalently via click chemistry by modifying the 5′ cap and the 3′ poly(A) tail with bioorthogonal functional groups. The resulting Cyclic Click Cap‐containing mRNA (CyCliCap‐mRNA) is translationally muted by a photocleavable protecting group. Upon irradiation by light, the photocleavable protecting group is removed and the respective linear Cap0‐mRNA released. We show that CyCliCap‐mRNA can be activated for efficient translation at late timepoints (24‐48 h post transfection) in HeLa cells, which is not possible with the respective linear mRNA. The light‐mediated opening releases linear mRNA with a native Cap0 and thus overcomes the problem of inefficient translation of circular RNAs.

## Graphical abstract

A clickable photo‐caged 5′ cap (CyCliCap) allows to make Cyclic Click Cap‐containing mRNA (CyCliCap‐mRNA) which is remarkably stable and translationally muted until irradiated, allowing for late‐stage activation of translation in cells by light.

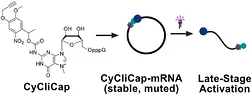

## 1 Introduction

**FIGURE 1:**
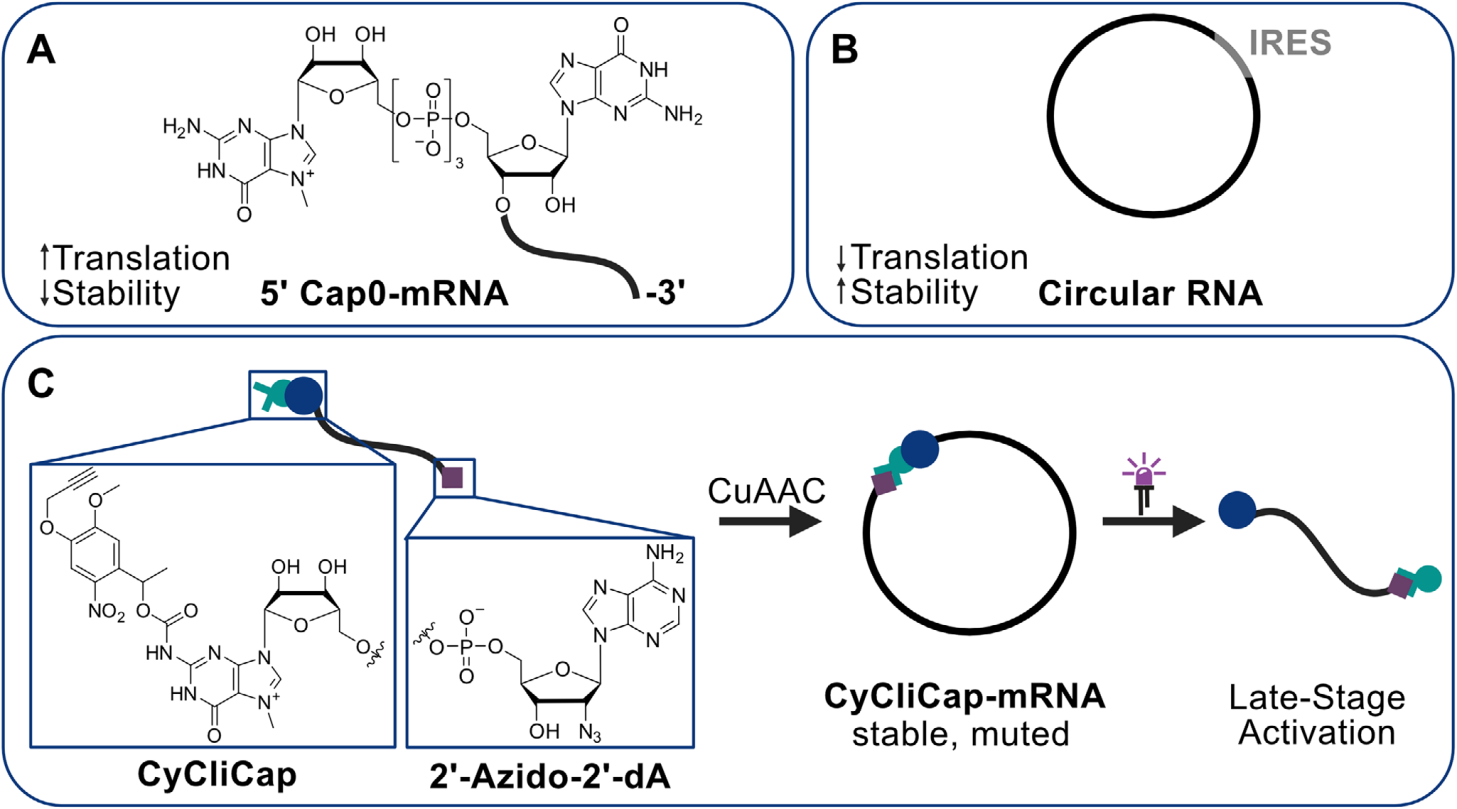
Concept of circular photocaged mRNA (CyCliCap‐mRNA) to increase cap‐caged mRNA stability for late‐stage activation of translation. A Eukaryotic mRNA with the native 5′ Cap0 structure for efficient translation initiation. The 5′ cap protects from 5′ end but not 3′ end degradation. B Circular RNAs (circRNAs) cannot be degraded from the 5′ nor the 3′ end and are highly stable. Naturally occurring circRNAs require an internal ribosome entry site (IRES) for translation initiation, but this is typically less efficient than cap‐dependent translation. C This work: Cyclic Click Cap (CyCliCap)‐mRNAs combine the advantages of circular RNAs and mRNAs. They require the photocaged 5′ cap with a clickable linker (CyCliCap) and an azido‐modification at the 3′ end (2′‐azido‐2′‐dA). Circularization is achieved by copper(I) catalyzed azide‐alkyne cycloaddition (CuAAC) in vitro and connects the loose ends, preventing the main routes of degradation. The resulting CyCliCap‐mRNAs are translationally muted until irradiated with UV‐light (LED icon). Upon irradiation, circular mRNA is linearized and the native Cap0 structure is unleashed, enabling high translational output even at late timepoints. Parts of this figure were created using biorender.com.

The recent success of mRNAs being approved as vaccines has raised hopes that they could also be suitable protein replacement agents. However, mRNAs would have to be more stable, yield higher amounts of protein and be less immunogenic to achieve this [1]. Several modifications of mRNAs are currently used in clinical applications or are being tested to increase the amount of protein produced. Most importantly the 5′ cap, which in its simplest form (Cap0) is an N7‐methylated guanosine linked to the transcription start nucleotide (TSN) by a 5′‐5′ triphosphate bridge, is required for efficient translation and reduced immunogenicity (Figure 1A). Cap1 with its additional 2′‐O‐methyl group at the TSN and additional natural or non‐natural modifications at the N^6^‐position of adenosine as TSN further improves the properties [2, 3, 4]. Internal modifications (e.g. m^1^Ψ) are also required to further reduce immunogenicity [5, 6]. In addition, sequence optimization of mRNA in the untranslated regions (UTRs), open reading frame, as well as the poly(A) tail proved important to enhance stability and translation efficiency [7, 8, 9, 10, 11, 12, 13]. Despite all efforts, mRNA stability in cells remains a major limitation.

mRNA can be degraded by multiple pathways and degradation from the ends is most relevant. Deadenylation‐dependent decay starts from the 3′ end followed by decapping and exoribonuclease digest from the 5′ end [14]. Deadenylation‐independent decay starts by decapping followed by exoribonuclease digestion from the 5′ end [15]. Both pathways are irrelevant in so‐called circular RNAs (Figure 1B). Circular RNAs can naturally be formed by back‐splicing and were shown to have higher stability [16]. Some of them can also be translated, however, they do not have the 5′ cap structure characterizing eukaryotic mRNAs and required for efficient translation.

Translation of circular mRNAs starts from an internal ribosomal entry site (IRES) and is typically less efficient than cap‐dependent translation [17]. The high stability of circular mRNAs has stimulated interest in their use for medical purposes. To maximize circRNA translation, factors such as vector topology, UTRs, internal ribosome entry sites have been optimized [18]. Synthetic aptamers recruiting the translation initiation machinery have been tested [18].

The addition of a noncovalent cap was found to initiate translation of circular mRNAs without IRESs [19, 20]. Recent work showed that equipping circular mRNA with a covalently connected 5′ cap can improve circular mRNA stability and translation [20, 21]. An alternative approach to stabilize mRNA was the recent incorporation of stability enhancing elements into mRNA [22]. Taken together, these recent reports show that circular mRNA remarkably increases the stability of mRNA in cells and in vivo. However, the translation of the circular constructs lags behind canonical mRNA with a 5′ cap and currently cannot be activated. Programmable control of translation would open the opportunity to build various layers for post‐transcriptional gene regulation, which can be interesting to build “smart vaccines” and artificial gene circuits in synthetic biology [23, 24].

We recently developed photocaged 5′ caps (FlashCaps) that enable the preparation of translationally muted mRNAs by in vitro transcription [25, 26, 27, 28]. These resulting FlashCap‐mRNAs can be activated by light. The activation in cells works very well at early timepoints (typically 6 h after transfection). At later timepoints (e.g. 12 h after transfection), the translation can also be activated, but the amount of reporter protein produced is much lower as a consequence of mRNA degradation [26, 29]. The stability of the FlashCap‐mRNAs was not improved compared to regular Cap0‐mRNA [25].

Here, we sought to generate circular mRNA by introducing a covalent connection between the photocaged 5′ cap and the poly(A) tail. The circular mRNA should be highly stable and OFF (i.e. translationally silent). Upon irradiation, the removal of the photocleavable protecting group should (*i*) linearize the circular mRNA and (*ii*) release the native Cap0 structure, leading to efficient translation (Figure 1C). The strength of this strategy is that it combines stability and already reduced translation of circular mRNA with the muting by the photocleavable protecting group in the OFF‐state, while irradiation releases linear mRNA with a native 5′ cap, which is the most efficient ON‐state for translation.

## 2 Results and Discussion

### 2.1 Design, Synthesis and Characterization of CyCliCap

**FIGURE 2:**
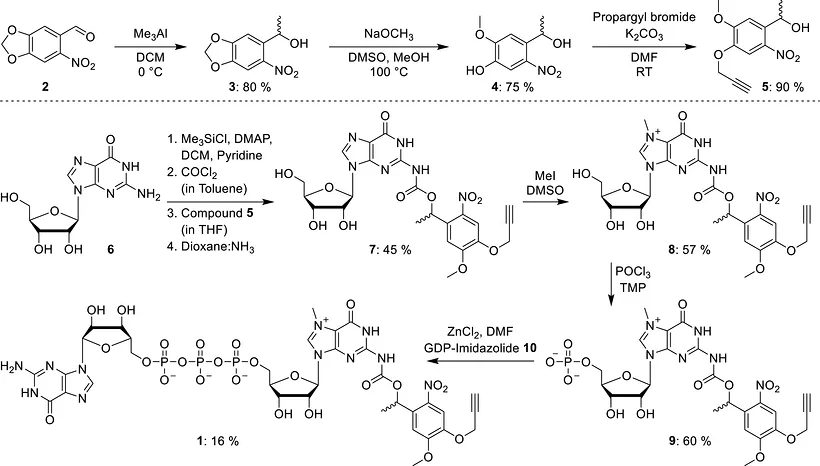
Synthesis of the CyCliCap (**1**). For details see text.

To achieve covalent circularization of mRNA via a photocleavable protecting group, we designed CyCliCap (**1**) (Figure 2). This compound is an analog of the 5′ Cap0 modified with a photocleavable protecting group containing an additional click handle. Previous work demonstrated that a photocleavable protecting group or another large modification at the N^2^‐position of the m^7^G efficiently abrogates binding of the translation initiation factor eIF4E and translation of an mRNA [25, 29, 30]. We chose the widely‐used and well‐characterized 6‐nitropiperonyl methyl (NPM) group, which has very good photo‐deprotection properties (≤ 15 s for NPM at 365 nm) [25] and follows a Norrish type 2 photocleavage mechanism [31, 32]. We derivatized this scaffold with a small propargyl group as click handle (**5**).

For the synthesis of **5**, commercially available 6‐nitropiperonal **2** was reduced to the secondary alcohol **3** via methylation with trimethyl aluminium. Subsequently, the five‐membered ring was opened using sodium methoxide, to obtain **4** with a phenolic hydroxy group. Compound **4** is then propargylated to obtain the clickable and photocleavable alcohol **5**. The synthesis of the CyCliCap **1** followed the previously reported synthetic procedure [25] with minor adjustments. The deprotection was performed in dioxane instead of THF, enabling direct lyophilization of crude‐silica mixture for flash chromatography. The N^2^‐modified guanosine **7** was methylated at the N7 position to **8**, prior to the phosphorylation of the guanosine 5′ OH to **9** to facilitate purification. Subsequent reaction of **9** with guanosine 5′ diphosphate (GDP)‐imidazolide **10** yielded the desired CyCliCap **1**, which was characterized by NMR and HRMS (Figure S18–S57).

**FIGURE 3:**
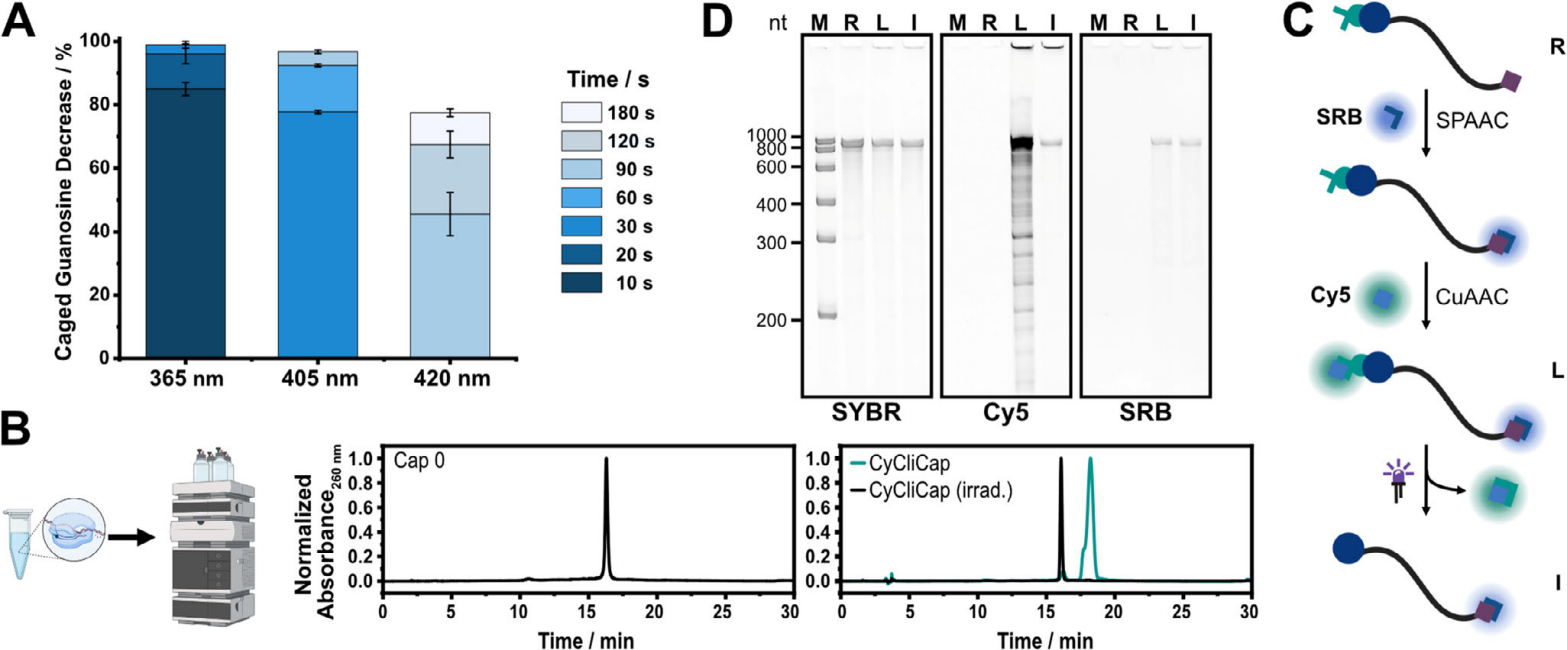
Characterization of CyCliCap and dual‐labeling of mRNA with 5′ CyCliCap and 2′‐azido‐dA at the 3′ end. A Photodeprotection of photocaged guanosine **7** at indicated wavelengths (365 and 405 nm at 140 mW/cm^2^, 420 nm at 56 mW/cm^2^) for different irradiation times. Mean values and standard deviation of *n*  =  3 independent experiments are shown. B HPLC purification of in vitro transcription producing GLuc‐mRNA with Cap0 or CyCliCap. mRNA with CyCliCap is shifted to a longer retention time (∼18 min) due to the hydrophobic effect. Irradiation of mRNA with CyCliCap removes the photocleavable group and releases Cap0‐mRNA with shorter retention time (∼16 min). C Schematic illustration of fluorophore labeling and removal to validate functionalized 5′ and 3′ ends. DBCO‐SRB was used to label the azido‐modified poly(A) tail via strain‐promoted azide‐alkyne cycloaddition (SPAAC). N_3_‐Sulfo‐Cy5 was used to label mRNA with CyCliCap via copper(I) catalyzed azide‐alkyne cycloaddition (CuAAC). Irradiation was performed for 30 s at 365 nm at 460 mW/m^2^. D PAA‐Gel analysis of GLuc‐mRNA dual labeling with CyCliCap and azido‐A at different stages of preparation, as illustrated in C. M: RiboRuler Low Range RNA Ladder. R: GLuc‐mRNA with CyCliCap and azido‐modified poly(A) tail; L: Dual‐labeled GLuc mRNA with CyCliCap‐Cy5‐label at the 5′ end and azido‐A‐Sulforhodamine (SRB)‐label at the 3′ end. I: Irradiated dual‐labeled GLuc‐mRNA with CyCliCap and removed Cy5 label and azido‐A‐SRB‐label retained. Parts of this figure were created using biorender.com.

To confirm that the clickable photocleavable group can be efficiently removed, we irradiated **7** in water:acetonitrile (1:1) with LEDs at 365 nm, 405 nm or 420 nm, respectively, and analyzed the photo‐deprotection by HPLC (Figure 3A, Table S1,S2, Figure S36, S37). We found that 30 s irradiation was sufficient to achieve 100% deprotection at 365 nm (140 mW/cm^2^). Longer wavelengths were also suitable: Irradiation at 405 nm (140 mW/cm^2^) required 90 s for complete deprotection. At 420 nm (56 mW/cm^2^) complete photodeprotection was not achieved, even at prolonged irradiation times (≥180 s, Figure 3A). Taken together, we established the synthesis of the CyCliCap, a 5′ Cap0 analog with a photocleavable protecting group at the N^2^‐position of m^7^G and an additional click handle and validated that it can be efficiently “uncaged” at 365 nm and 405 nm.

### 2.2 In Vitro Transcription of mRNA and Dual Labeling of mRNA With CyCliCap

Next, we wanted to introduce CyCliCap into mRNA and see whether it can be used for subsequent fluorescent labeling with click chemistry. We used in vitro transcription (IVT) with T7 RNA polymerase in the presence of CyCliCap to prepare mRNA with this 5′ cap (Figure 3B). After optimization, we used magnesium acetate as source for Mg^2+^ to prevent precipitation of the lipophilic CyCliCap. To purify mRNA with CyCliCap from uncapped RNA, we benefited from the hydrophobic effect of the CyCliCap and were able to use HPLC, as previously demonstrated for PureCap and TCO‐cap [30, 33]. IVT with Cap0 shows only one peak, but it contains Cap0‐mRNA and uncapped RNA (Figure 3B, left chromatogram). In contrast, IVT with CyCliCap shows two well‐separated peaks on HPLC separation, corresponding to uncapped RNA and mRNA with CyCliCap (Figure S1A), facilitating purification. Control mRNAs, i.e. Cap0‐mRNA and ApppG‐mRNA (used as negative control for cap‐independent translation) were routinely treated with 5′ polyphosphatase followed by digestion with exonuclease XRN1 to degrade uncapped RNA and retain only capped mRNA. Capping efficiencies and yields from IVTs for GLuc‐mRNA with CyCliCap were 31% and 370 ng/µL, which were lower than for the controls with Cap0 (47%, 550 ng/µL) and ApppG (61%, 600 ng/µL), but in the same order of magnitude (Figure S1). In addition to GLuc‐mRNA, two additional reporter‐mRNAs were produced with the indicated 5′ cap, namely HiBiT‐ and eGFP‐mRNA (Figure S1).

For the envisaged covalent circularization of mRNA ends, we needed a second compatible clickable group at the 3′ end. Ideally, the mRNA should also contain a poly(A) tail, as this increases stability and translation [34, 35]. We and others had previously used 2′‐azido‐dATP in combination with yeast poly(A) polymerase to label mRNA at the poly(A) tail [36, 37, 38]. In this method, mRNA is extended at the poly(A) tail with 2′‐azido‐2′‐dATP and subsequently labeled via strain‐promoted azide‐alkyne cycloaddition (SPAAC). Importantly, we had demonstrated that this labeling method incorporates multiple azido‐adenosine nucleotides and does not impair translation but rather increases the amount of protein produced [36, 37]. Therefore, we now aimed to combine the 5′ cap labeling of mRNA with CyCliCap with this 3′ poly(A) tail labeling (Figure 3C and Figure S2).

To achieve this, we prepared mRNA with a CyCliCap and applied the poly(A) tail labeling with 2′‐azido‐2′‐dATP (Figure 3D, “R”). A sulforhodamine (SRB)‐cyclooctyne conjugate (DBCO‐SRB, Figure S3 for the structure) was clicked to the poly(A) tail via SPAAC. In a second step, we subjected the alkyne group of CyCliCap to a copper(I) catalyzed azide‐alkyne cycloaddition (CuAAC) with N_3_‐Sulfo‐Cy5 (Figure S3 for the structure). This dually labeled mRNA (“L”, Figure 3C) was irradiated (30 s, 365 nm, 460 mW/cm^2^) to remove the photocleavable protecting group including the conjugated Cy5 fluorophore (Figure 3C, “I”). All three steps were followed by gel analysis and SYBR Gold staining as well as fluorescent imaging in the Cy5 and SRB channels (Figure 3C,D).

To our delight, gel analysis of the dually labeled mRNA (“L”) showed a band at the expected length in both the Cy5 and the SRB channel (Figure 3D), indicating that both bioorthogonal groups had been successfully introduced and reacted. The fluorescence signal is absent before labeling (“R”), although the RNA is clearly visible in the SYBR gold channel. After irradiation (“I”), the Cy5 signal becomes much weaker, showing that the intended removal of the photocleavable protecting group indeed cleaves the Cy5 fluorophore. In the SRB‐channel, the bands for “L” and “I” are of similar intensity, showing that the SRB‐label is not affected by the photocleavage. The Cy5 signal was much stronger than the SRB signal. This is most likely the result of incomplete poly(A) tail labeling, as previously observed [36].

Taken together, CyCliCap can be used for in vitro transcription and facilitates purification of the resulting mRNAs by HPLC. We showed that mRNA with CyCliCap can be labeled at both ends and that the 5′ end label can be removed by irradiation. These data suggest that both ends of the mRNA are available for the intended covalent circularization via CuAAC.

### 2.3 Generation and in Vitro Characterization of Cyclic Click Capped mRNA (CyCliCap‐mRNA)

**FIGURE 4:**
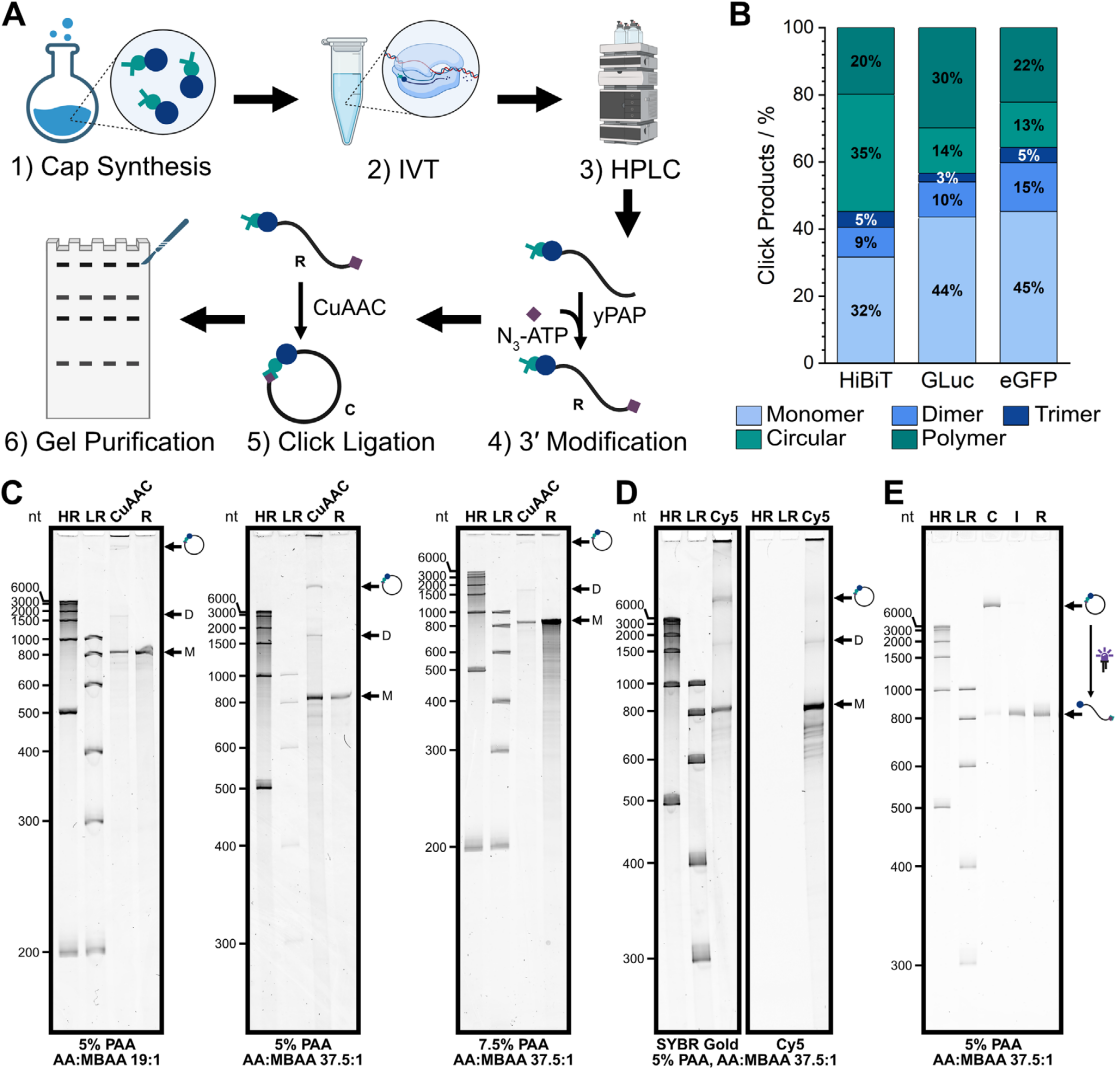
Circularization of mRNA via splint‐guided click ligation. A Six steps to produce Cyclic Click Cap‐containing mRNA (CyCliCap‐mRNA). The synthesized CyCliCap was used for in vitro transcription (IVT), the produced mRNA with a 5′ CyCliCap was purified by HPLC. After 3′ end modification with yeast poly(A) polymerase (yPAP) and 2′‐azido‐2′‐dATP (N_3_‐ATP), CyCliCap‐pre‐mRNA was obtained and circularized to CyCliCap‐mRNA using copper(I) catalyzed azide‐alkyne cycloaddition (CuAAC) and a DNA splint. Circular mRNA was purified via PAA gel electrophoresis. B Relative quantification of observed click products from gels using ImageJ (Table S3–S5 and Figure S5–S7) C Identification and confirmation of circular mRNA (801 nt) using gel shift assay. Migration of circular RNAs differs depending on gel composition **[** 39 **]** M: Monomer. D: Dimer. D Cy5‐Labeling of the crude click ligation mixture with N_3_‐Sulfo‐Cy5 to identify available alkyne at the 5′ cap. Note that only linear species are labeled. E Light‐induced in vitro linearization of gel‐purified CyCliCap‐mRNA (365 nm, 30 s, 460 mW/cm^2^) confirms that the resulting mRNA runs like the linear control CyCliCap‐pre‐mRNA (R). HR: RiboRuler High Range RNA ladder. LR: RiboRuler Low Range RNA ladder. CuAAC: Crude click mixture. R: Poly(A) modified GLuc‐mRNA with a 5′ CyCliCap. Cy5: Crude click mixture labeled with N_3_‐Sulfo‐Cy5. C: CyCliCap‐GLuc‐mRNA. I: Irradiated CyCliCap‐GLuc‐mRNA. AA: Acrylamide; MBAA: N,N’‐methylenebisacrylamide. Parts of this figure were created using biorender.com.

Based on these insights we developed the covalent circularization of mRNA via click chemistry by modifying the 5′ cap and the 3′ poly(A) tail with bioorthogonal functional groups. The resulting Cyclic Click Cap‐containing mRNA is termed CyCliCap‐mRNA. The successful approach consists of six steps ((Figure 4A): 1) Synthesis of CyCliCap, 2) IVT to prepare mRNA with CyCliCap, 3) HPLC purification of mRNA with CyCliCap (removal of uncapped RNA and shorter products), 4) poly(A) tail labeling with 2′‐azido‐2′‐dATP (N_3_‐ATP) to obtain CyCliCap‐pre‐mRNA, 5) splint‐guided click ligation of 5′ and 3′ ends containing CyCliCap and 2′‐azido‐2′‐dATP via CuAAC, 6) gel purification of the crude click mixture to isolate circular mRNA from other linear click products.

Steps 1–4 were described above. For step 5, the splint‐ligation, we worked under highly dilute conditions to favor intra‐ over intermolecular reaction. After optimization, typically 0.25 µM RNA were annealed with equimolar amount of DNA splint in a temperature gradient (from 80°C to 25°C in 45 min). It was important to precipitate the RNA/DNA hybrid after annealing and perform the CuAAC reaction in 50% (v/v) DMSO to obtain good conversion. The reaction was performed for 30 min at 45°C and directly quenched by addition of EDTA, followed by purification on a PAA gel (Figure 4A). Regarding the RNA construct, it was important to have an additional sequence (40 nt) downstream of the genetically encoded poly(A) tail, to aid annealing (Figure S4). Three different splint constructs (with and without spacers) were tested for this purpose but performed in a similar manner with respect to circularization efficiency, letting us choose the simplest construct (Figure S4).

Gel analysis of the CuAAC reaction revealed several bands, indicating formation of different products (Figure 4C, S5–S7). We could readily identify concatemers, i.e. dimers and trimers, by their length with the help of RNA ladders (Figure 4C, S5–S7). The identification of circularized mRNA was at first difficult. We then benefited from the observation that circular RNAs migrate differently depending on gel percentage with respect to the RNA ladder [39] and optimized the gel composition accordingly. For long RNAs (GLuc‐, eGFP‐mRNA), the gel pore size had to be increased to enable migration in PAA gels. This was achieved by increasing the ratio of acrylamide to methylenebisacrylamide from 19:1 to 37.5:1. Changing the ratio from 19:1 to 37.5:1 in a 5 % (but not in 7.5 %) PAA gel caused a significant gel shift for one band, which was identified as the circular mRNA, due to its aberrant gel migration behavior (Figure 4C). After optimizing the PAA gels, we were able to separate CyCliCap‐mRNA from linear products. We performed the cyclization for three reporter mRNAs of different lengths, i.e. HiBiT‐mRNA (Figure S5), GLuc‐mRNA (Figure 4C, Figure S6) and eGFP‐mRNA (Figure S7) and calculated the yields and product distribution from the respective gels. Relative quantification of all click products from the product bands of all three constructs is summarized in Figure 4B. For the relatively short HiBiT‐mRNA (267 nt), a circularization efficiency of around 35 % was observed, while 32 % of the mRNA remained unreacted. The circularization efficiency decreased to 14 % for GLuc mRNA (801 nt) with 44 % remaining as monomers. This reduction of circular mRNA formation was expected as the probability of bringing 5′ cap and poly(A) tail in proximity decreases for longer transcripts. Dimer and trimer formation remained constant at around 10 % and 5 %, respectively, as these result from intermolecular reactions. Polymer formation was around 20 % to 30 %. A limiting factor for the yield is likely the incomplete modification of the 3′ end with N_3_‐ATP [36].

To independently confirm that the isolated CyCliCap‐mRNA is indeed circular, we labeled all reaction products via the CuAAC reaction. Only non‐circularized mRNA should contain non‐reacted terminal alkyne, allowing for N_3_‐Sulfo‐Cy5 labeling. As expected, gel analysis showed Cy5‐labeling for linear RNA species (monomer, dimer and polymer) (Figure 4D). To our delight, the CyCliCap‐mRNA band was not labeled with Cy5, indicating that the alkyne of the CyCliCap had been completely conjugated into the cyclic product.

Finally, we tested whether CyCliCap‐mRNA would indeed undergo light‐induced linearization. To this end, we used the purified CyCliCap‐mRNA and irradiated it (365 nm, 10 s, 460 mW/cm^2^). Subsequent gel analysis showed that the migration of the band was drastically shifted after irradiation and migrated at the expected length of the GLuc‐mRNA (Figure 4D). A control that had not been irradiated retained the migration behavior of the CyCliCap‐mRNA and a control of a cap0‐GLuc‐mRNA with a modified poly(A) tail migrated similar to the irradiated CyCliCap‐mRNA (Figure 4D). Similar experiments with HiBiT‐mRNA and eGFP‐mRNA are shown in Figures S8–S11. Taken all together, the data obtained from the gel shift assay, Cy5 labeling and the light‐induced linearization show that the CyCliCap‐mRNA is circular and light‐responsive.

### 2.4 Translation Studies of Cyclic Clicked Capped mRNA (CyCliCap‐mRNA) in Cells

**FIGURE 5:**
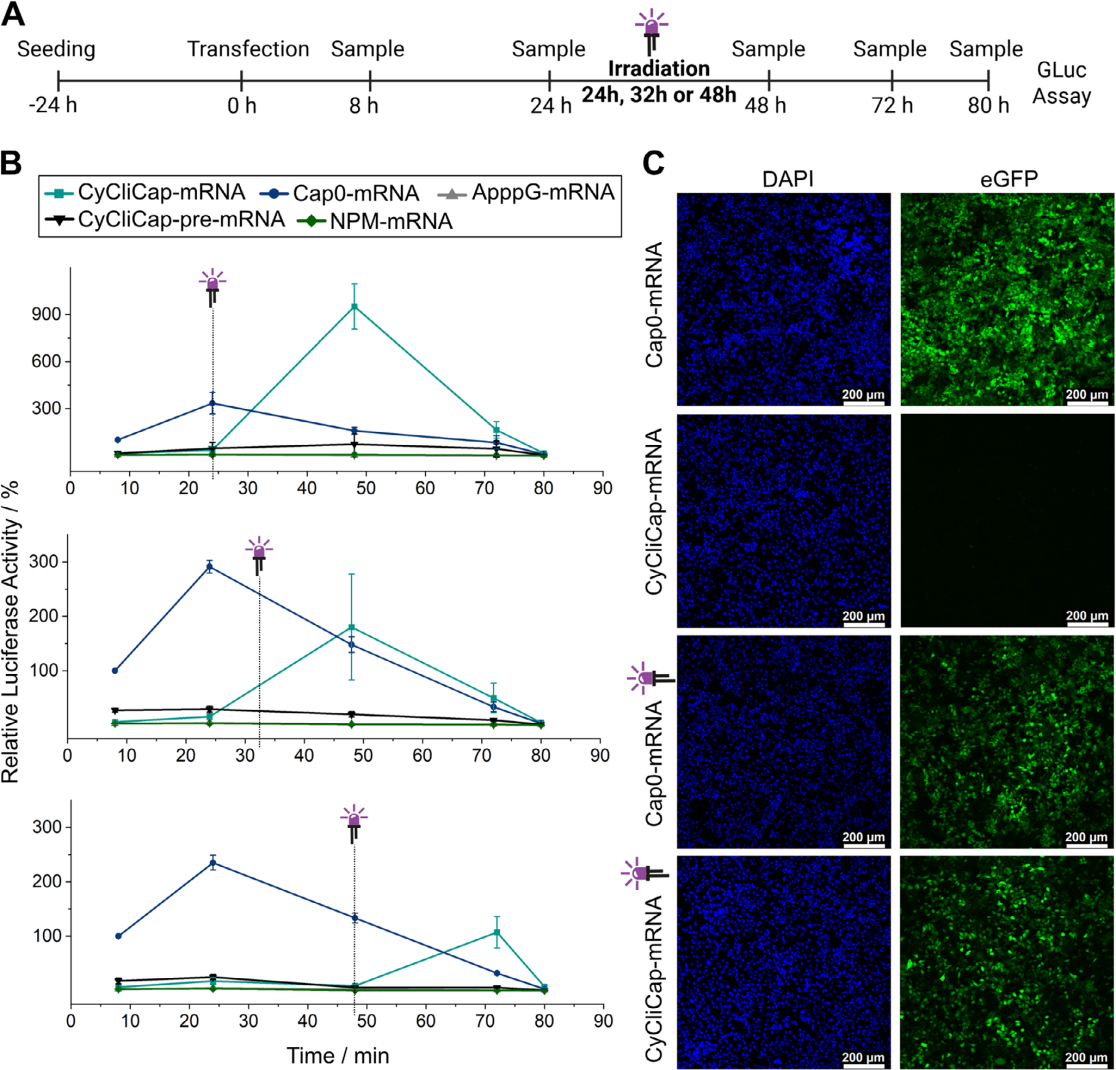
CyCliCap‐mRNAs are translationally muted and enable late‐stage activation of translation in HeLa cells. A Scheme illustrating the experimental setup of the luciferase assay. The supernatant was collected at each time point and the media was changed. 24 h, 32 h or 48 h after transfection, all cells were irradiated (365 nm, 10 s, 460 mW/cm^2^). Luciferase assay was performed at indicated timepoints until 80 h post transfection. B Relative protein output from HeLa cells transfected with differently capped GLuc‐mRNAs. CyCliCap‐mRNA (circular); CyCliCap‐pre‐mRNA (linear precursor of CyCliCap‐mRNA before circularization); Cap0‐mRNA (with Cap0 and linear, i.e. canonical); ApppG‐mRNA (with ApppG‐cap and linear, i.e. cap‐independent translation), NPM‐mRNA (with NPM‐FlashCap and linear). Mean values and standard deviation of *n*  =  3 independent experiments are shown. For the 80 h time‐point, *n* = 2 independent replicates are shown. All values are normalized to Cap0‐GLuc‐mRNA at 8 h post transfection (= 100%). C Fluorescence confocal microscopy images of HeLa cells transfected with CyCliCap‐eGFP‐mRNA or control mRNAs. Cells were either kept in the dark or irradiated (365 nm, 10 s) at 24 h after transfection (indicated by LED icon). Cap0‐eGFP‐mRNA serves as a positive control. Samples were imaged for eGFP fluorescence 48 h after transfection. Shown is one representative set of *n * =  3 independent replicates. Parts of this figure were created using biorender.com.

Finally, we wanted to see whether CyCliCap‐mRNAs would be translated or muted in cells and whether light can be used to activate their translation at different timepoints. To this end, we prepared CyCliCap‐GLuc‐mRNAs as described above and used them to transfect HeLa cells. The resulting GLuc protein is secreted, allowing us to measure the amount of protein produced from the mRNA in the medium (Figure 5A). The cells were irradiated (365 nm, 10 s, 460 mW/cm^2^) after 24 h, 32 h or 48 h. The luciferase activity of all samples was normalized to the positive control, which was the Cap0‐GLuc‐mRNA at 8 h post transfection (Figure 5B, upper panel, 100%; 24 h, 330 %). Compared to the positive control, the CyCliCap‐GLuc‐mRNA was translationally muted, showing only 10–40 % of relative luciferase activity at 8 to 24 h post transfection (Figure 5B). This was close to the negative control, an ApppG‐capped‐GLuc‐mRNA, which benchmarks cap‐independent translation (Figure 5B, 5 % for 8 and 24 h). After irradiation at 24 h post‐transfection, the luciferase activity originating from CyCliCap‐GLuc‐mRNA drastically increased to remarkably high 950 % (upper panel, 48 h post transfection). This clearly shows that the light‐mediated cleavage of CyCliCap works also in cells and that the released Cap0‐GLuc‐mRNA can be efficiently translated. The high level of luciferase activity indicates that the muted CyCliCap‐GLuc‐mRNA had remained stable for this long period of time. The observation that the translational output of CyCliCap‐mRNA after irradiation is even higher than the positive control (Cap0‐mRNA) could originate from several effects (e.g., remaining covalent 3′ modification or reduced immunogenicity [40, 41]) and warrants further investigation.

To obtain further insights into how stable CyCliCap‐mRNAs are, we also investigated light‐mediated activation of translation at later timepoints—up to 56 h post transfection. Remarkably, CyCliCap‐mRNA enabled activation of translation at all of these timepoints. Specifically, irradiation at 32 h post‐transfection still led to similar luciferase levels as the Cap0‐GLuc mRNA (Figure 5B, middle panel: 48 h, 180%). Irradiation at 48 h (Figure 5B, lower panel) and even 56 h (Figure S12) also activated CyCliCap‐mRNA for translation, albeit to a lower extent. Importantly, the controls with linear mRNA—the CyCliCap‐pre‐mRNA (same modifications, no circularization) as well as the NPM‐FlashCap‐mRNA (photocaged 5′ cap, no poly(A) modification)—could not be activated for translation at 24 h post‐transfection (Figure 5B, 24 h). At 72 h post transfection (Figure 5B, 72 h) the amount of produced protein is decreased for all samples and nearly no luciferase activity is detected after this timepoint. For unirradiated HeLa cells kept in dark over 80 h, the translation of Cap0‐GLuc‐mRNA, ApppG‐GLuc‐mRNA and the linear CyCliCap‐pre‐GLuc‐mRNA is comparable to that of the irradiated samples (Figure S13). The CyCliCap‐GLuc‐mRNA remains translationally muted, showing no significant luciferase activity.

We wondered whether the remarkable activation of mRNA translation could be attributed to the circularity or whether the concatemers would suffice to stabilize the mRNA and activate its translation at late timepoints. To find out, we also isolated the side products of the click reaction from the gel (Figure 4B, Figure S6) and used them to transfect HeLa cells for the same experiment (Figure S14). However, none of the side‐products reached the luciferase activity of the positive control (Cap0‐GLuc‐mRNA). Furthermore, only the polymer fraction could be activated at 24 h post‐transfection, reaching similar protein levels as the Cap0 sample at this time point.

Finally, we aimed to confirm the translation assay of CyCliCap‐mRNA with a different reporter and an independent assay. We therefore prepared CyCliCap‐eGFP‐mRNA as described above and used it to transfect HeLa cells. These cells were either kept in the dark or irradiated at 24 h post transfection. Cells were fixed at 48 h post‐transfection and analyzed by confocal fluorescence microscopy (Figure 5C, S15–S17). In the absence of light, CyCliCap‐eGFP‐mRNA did not yield green fluorescing cells, indicating that the mRNA is muted. When irradiated, the CyCliCap‐eGFP‐mRNA resulted in bright green fluorescence, similar to the positive control (Cap0‐eGFP‐mRNA). It should be noted that in case of eGFP production the images represent the total amount of protein produced during the experiment. Compared to the positive control, the irradiated CyCliCap‐mRNA starts translation 24 h later, accounting for the observed lower green fluorescence. Taken together, these data show that CyCliCap‐mRNAs are translationally muted and can be successfully activated at remarkably late time points. Strikingly, the total protein output produced from CyCliCap‐GLuc‐mRNA outperformed Cap0‐GLuc‐mRNA.

## 3 Conclusion

This strategy combines the reduced translation of circular mRNA with the muting by the photocleavable protecting group for an efficient OFF‐state. Circular mRNAs have gained attention due to their high stability, owing to a lack of degradation from the ends. However, they are normally not as well translated as they lack a 5′ cap structure. Similar to other cyclic RNAs, CyCliCap‐mRNA is circular and cannot be degraded from the ends. However—in contrast to previous designs—irradiation releases the canonical linear Cap0‐mRNA and therefore produces an efficient ON‐state. Therefore, CyCliCap‐mRNA shows little background and enables remarkable activation at considerably later timepoints than the respective photocaged linear mRNA. The approach bears potential for long‐term applications as tool for conditional activation of ectopic mRNAs in cells and model organisms and provides an avenue towards time‐dependent dosing of mRNA therapeutics. For the current study in cultured mammalian cells, a few hundred nanograms of CyCliCap‐mRNA were sufficient. For future studies requiring larger amounts, the individual steps of the preparation protocol need to be improved. In particular, the capping efficiency, 3′ modification, click reaction and gel purification could be optimized.

## Author Contributions

**Bayram Terzi**: conceptualization, investigation, data curation, writing – original draft, writing – review and editing, methodology, validation, visualization. **Greta Charlotte Dahm**: conceptualization, investigation, writing – original draft, writing – review and editing, methodology, validation, visualization, data curation. **Kristoffer Heintz**: investigation, methodology, validation. **Martin Sumser**: investigation, visualization, writing – review and editing, data curation. **Yasuaki Kimura**: funding acquisition, supervision, writing – review and editing. **Hiroshi Abe**: funding acquisition, supervision, writing – review and editing. **Andrea Rentmeister**: conceptualization, funding acquisition, writing – original draft, writing – review and editing, project administration, supervision.

## Conflicts of Interest

The authors declare no conflict of interest.

## Supporting information



The authors have cited additional references within the Supporting Information [42, 43].**Supporting File**: anie73709‐sup‐0001‐SuppMat.pdf.

## Data Availability

The data that supports the findings of this study are available in the supplementary material of this article
