# Supplementary material for: Circular Photocaged mRNA for Light‐induced Late‐stage Activation of Translation
Source: Angew Chem Int Ed Engl. 2026 Jul 27;65(39):e8731921. doi: 10.1002/anie.8731921 (PMC13592297; doi:10.1002/anie.8731921)
Supplement: Supplementary file 1 [file ANIE-65-e8731921-s001.pdf]

Supplementary Information

1. General Procedures 2

Preparation of capped mRNA 2

Sequences of the PCR products 2

3' End modification with yeast poly(A) polymerase 4

Fluorophore clicking to modified 3' end (SPAAC reaction) 4

Fluorophore clicking to CyCliCap (CuAAC reaction) 4

mRNA circularization 4

Irradiation 5

In-cell luminescence assay 5

Microscopy 5

2. Supplementary Tables 7

3. Supplementary Figures 9

4. Chemical Synthesis 20

1-(6-Nitrobenzo[*d*][1,3]dioxol-5-yl)ethan-1-ol (3) 20

4-(1-Hydroxyethyl)-2-methoxy-5-nitrophenol (4) 20

1-(4-((Propargyl)oxy)-5-methoxy-2-nitrophenyl)ethan-1-ol (5) 21

N^2-(1-(4-((Propargyl)oxy)-5-methoxy-2-nitrophenyl)ethyl)oxycarbonylguanosine (7) 21

N^2-(1-(4-((Propargyl)oxy)-5-methoxy-2-nitrophenyl)ethyl)oxycarbonyl-7-methyl-guanosine (8) 22

N^2-(1-(4-((Propargyl)oxy)-5-methoxy-2-nitrophenyl)ethyl)oxycarbonyl-7-methyl-guanosine 5'-phosphate (9) 23

Guanosine 5'-diphosphate imidazolide (10) 23

CyCliCap (1) 23

5. Compound Characterization 24

6. References 47

1. General Procedures

Preparation of capped mRNA

Capped mRNA was prepared via *in vitro* transcription by wild-type T7 RNA polymerase from dsDNA template. The DNA template was generated by PCR and purified with the NucleoSpin Gel and PCR Clean-up kit (Machery-Nagel). In vitro transcription was performed in transcription buffer (40 mM Tris base, 2 mM spermidin, 0.01 % Triton X-100, 1.5 % PEG6000, 5 mM DTT, 15 mM magnesium actetate) using 10-15 ng/µL DNA template, 3.2 mM cap analog, 2 mM ATP, 2 mM CTP, 2 mM UTP, 1.5 mM GTP with T7 RNA polymerase (2.5 mg/mL, in-house), Ribolock (Thermo) and pyrophosphatase (0.004 U/µL, Thermo) for 3 h at 37 °C. Subsequently, DNA template was digested by DNase I (12.5 U per µg DNA template, Thermo) for 30 min at 37 °C and crude IVT mixture was purified using Monarch Spin RNA Cleanup Kit (New England Biolabs). Linear CyCliCap-mRNA was directly HPLC purified on YMC-Triart Bio C4 column at 50 °C (0-5 min 100 % buffer A: 50 mM triethylammonium acetate (TEAA) with 5 % acetonitrile (MeCN), 5-30 min gradient to 25 % buffer B: 100 % MeCN). Since Cap 0- and ApppG cap-mRNA cannot be separated from uncapped mRNA on HPLC, uncapped RNA was removed by XRN1 digestion prior to HPLC purification. For this, IVT product (5 µg) was incubated with RNA 5′ polyphosphatase (10 U, Biosearch Technologies) in 1x reaction buffer at 37 °C for 2 h, converting 5′ triphosphorylated RNA to 5′ monophosphorylated RNA. Subsequently, magnesiumchloride (4.6 mM) was added and uncapped RNA was digested by exonuclease XRN1 (2 U, New England Biolabs) at 37 °C for 1.5 h. The digestion was purified using Monarch Spin RNA Cleanup Kit (New England Biolabs) before injection on HPLC. HPLC purified mRNA was precipitated with isopropanol and resolved in water.

Sequences of the PCR products

Following PCR products were used for IVT. The generated RNA sequence initiates with G and is underlined. The open reading frame coding for the respective protein is written in bold letters.

PCR product used for the IVT of 185 nt RNA (5′ to 3′):

TAATACGACTCACTATAGGGAAATAAGAGAGAAAAGAAGAGTAAGAAGAAATATAA
GAGAATTCGCCACCATGGGAGTCAAAGTTCTGTTTGCCCTGATCTGCATCGCTG
TGGCCGAGGCCAAGCCCACCGAGAACAACGAAGACTTCAACATCGTGGCCGTG
GCCAGCAACTTCGCGACCACGGATCTCGATGCTGACCGC

PCR product used for the IVT of HiBiT mRNA (5′ to 3′):

TAATACGACTCACTATAGGGAAATAAGAGAGAAAAGAAGAGTAAGAAGAAATATAA
GAACC**ATGGTGAGCGGCTGGCGGCTGTTCAAGAAGATTAGCTAA**GCTGCCTTC
TGCGGGGCTTGCCTTCTGGCCATGCCCTTCTTCTCTCCCTTGCACCTGTACCTC

TTGGTCTTTGAATAAAGCCTGAGTAGGAAGAATATTAAAAAAAAAAAAAAAAAAAA
AAAAAAAAAAAAAAAAAAAAAAAAATTAATTAACATATGGCGGCCGCTCTAGAACT
AGTGTCGAC

PCR product used for the IVT of GLuc mRNA (5′ to 3′):

TAATACGACTCACTATAGGGAAATAAGAGAGAAAAGAAGAGTAAGAAGAAATATAA
GAGAATTCGCCACC**ATGGGAGTCAAAGTTCTGTTTGCCCTGATCTGCATCGCTG**
TGGCCGAGGCCAAGCCCACCGAGAACAACGAAGACTTCAACATCGTGGCCGT
GGCCAGCAACTTCGCGACCACGGATCTCGATGCTGACCGCGGGAAGTTGCCC
GGCAAGAAGCTGCCGCTGGAGGTGCTCAAAGAGATGGAAGCCAATGCCCGGA
AAGCTGGCTGCACCAGGGGCTGTCTGATCTGCCTGTCCCACATCAAGTGCACG
CCCAAGATGAAGAAGTTCATCCCAGGACGCTGCCACACCTACGAAGGCGACA
AAGAGTCCGCACAGGGCGGCATAGGCGAGGCGATCGTCGACATTCCTGAGAT
TCCTGGGTTCAAGGACTTGGAGCCCATGGAGCAGTTCATCGCACAGGTCGATC
TGTGTGTGGACTGCACAACTGGCTGCCTCAAAGGGCTTGCCAACGTGCAGTGT
TCTGACCTGCTCAAGAAGTGGCTGCCGCAACGCTGTGCGACCTTTGCCAGCA
AGATCCAGGGCCAGGTGGACAAGATCAAGGGGGCCGGTGGTGACTAAGGATC
CGCTGCCTTCTGCGGGGCTTGCCTTCTGGCCATGCCCTTCTTCTCTCCCTTGCA
CCTGTACCTCTTGGTCTTTGAATAAAGCCTGAGTAGGAAGAATATTAAAAAAAAAA
AAAAAAAAAAAAAAAAAAAAAAAAAAAAAAAAAAATTAATTAACATATGGCGGCCG
CTCTAGAACTAGTGTCGAC

PCR product used for the IVT of eGFP mRNA (5′ to 3′):

TAATACGACTCACTATAGGGAAATAAGAGAGAAAAGAAGAGTAAGAAGAAATATAA
GAGAATTCGCCACC**ATGGTGAGCAAGGGCGAGGAGCTGTTCACCGGGGTGGT**
GCCCATCCTGGTCGAGCTGGACGGCGACGTAAACGGCCACAAGTTCAGCGTG
TCCGGCGAGGGCGAGGGCGATGCCACCTACGGCAAGCTGACCCTGAAGTTCA
TCTGCACCACCGGCAAGCTGCCCGTGCCCTGGCCCACCCTCGTGACCACCCT
GACCTACGGCGTGCAGTGCTTCAGCCGCTACCCCGACCACATGAAGCAGCAC
GACTTCTTCAAGTCCGCCATGCCCGAAGGCTACGTCCAGGAGCGCACCATCTT
CTTCAAGGACGACGGCAACTACAAGACCCGCGCCGAGGTGAAGTTCGAGGGC
GACACCCTGGTGAACCGCATCGAGCTGAAGGGCATCGACTTCAAGGAGGACG
GCAACATCCTGGGGCACAAGCTGGAGTACAACTACAACAGCCACAACGTCTAT
ATCATGGCCGACAAGCAGAAGAACGGCATCAAGGTGAACTTCAAGATCCGCC
ACAACATCGAGGACGGCAGCGTGCAGCTCGCCGACCACTACCAGCAGAACAC
CCCCATCGGCGACGGCCCCGTGCTGCTGCCCGACAACCACTACCTGAGCACC
CAGTCCGCCCTGAGCAAAGACCCCAACGAGAAGCGCGATCACATGGTCCTGC
TGGAGTTCGTGACCGCCGCCGGGATCACTCTCGGCATGGACGAGCTGTACAA
GTAGGGATCCGCTGCCTTCTGCGGGGCTTGCCTTCTGGCCATGCCCTTCTTCTC
TCCCTTGCACCTGTACCTCTTGGTCTTTGAATAAAGCCTGAGTAGGAAGAATATTA

AAAAAAAAAAAAAAAAAAAAAAAAAAAAAAAAAAAAAAAAAAAAAATTAATTAACATAT GGCGGCCGCTCTAGAACTAGTGTCGAC

3′ End modification with yeast poly(A) polymerase

For introduction of azido groups at the 3′ end, mRNA (150-200 ng/µL) was incubated with 2′-azido-2′-dATP (1 mM, Jena Bioscience) and yeast poly(A) polymerase (30 U/µL, Thermo Fisher Scientific) in 1x poly(A) polymerase reaction buffer at 37 °C for 1 h. The reaction was purified using RNA Clean & Concentrator-5 kit (Zymo Research) and the mRNA eluted in water.

Fluorophore clicking to modified 3′ end (SPAAC reaction)

Azido-modified mRNA (1 µg) was incubated with DBCO-SRB (75 µM) at 37 °C for 1 h. Unclicked fluorophore was removed via ethanol precipitation of the mRNA.

Fluorophore clicking to CyCliCap (CuAAC reaction)

Linear CyCliCap-mRNA (1 µg) was mixed with azido-sulfo-Cy5 (40 µM), DMSO (50 % v/v) and premixed $CuSO_4$ (5 mM) and THPTA (25 mM). The reaction was initiated with addition of sodium ascorbate (100 mM) and incubated at 45 °C for 30 min. Subsequently, EDTA was added in excess to quench the reaction and the RNA precipitated with ethanol to remove unclicked fluorophore.

mRNA circularization

Azido-modified linear CyCliCap-mRNA (0.25 µM) was mixed with DNA splint (0.25 µM) in annealing buffer containing HEPES (50 mM) and KCl (150 mM), heated to 80 °C and cooled to 25 °C within 45 min. Subsequently, salts were removed via ethanol precipitation. Click ligation of annealed mRNA (0.25 µM) was performed in DMSO (50 % v/v) with $MgCl_2$ (5 mM) and premixed $CuSO_4$ (5 mM) and THPTA (25 mM). Sodium ascorbate (100 mM) was added to start the reaction. The mixture was incubated at 45 °C for 30 min, then quenched with EDTA and the RNA precipitated with isopropanol. The click products were separated on a denaturing polyacrylamide gel with either 19:1 or 37.5:1 acrylamide to methylenebisacrylamide ratio. The gel was stained with SYBR Gold and scanned on an Amersham Typhoon biomolecular imager (Cytiva). Respective bands were cut out and the desired click product isolated via crush&soak method.

Sequence of the used DNA splint (5′ to 3′):

TCTTTTCTCTCTTATTTCCCGTCGACACTAGTTCTAGAGC

Irradiation

Caged guanosine (10 µL, 500 µM in 1:1 acetonitrile:water) was irradiated in a PCR tube with an in-house made LED box and analyzed by HPLC. LEDs emitting at 365, 405 and 420 nm were used. The LED lamp was positioned directly above the open PCR tube with a distance of 1.5 cm between LED and solution. The actual power of the LED lamp was measured 140 mW/cm^2 for 365 nm and 405 nm LEDs and 56 mW/cm^2 for 420 nm LED.

mRNA (100 ng, 5 µL) in water was irradiated for 30 s in a PCR tube with custom-made LED setup from Thorlabs at 21 °C using a UV-A-LED (λ_{max} = 365 nm) with an electric current of 1400 mA and a measured LED output power of 460 mW/cm^2.

In-cell luminescence assay

HeLa cells (Merck, 93021013-1VL) were cultured in MEM Earle's medium (Sigma Aldrich) supplemented with L-glutamine (2 mM, Thermo Fisher Scientific), non-essential amino acids (1 %, Sigma Aldrich) and fetal calf serum (FCS; 10 %, PAN) under standard conditions (5 % CO_2, 37 °C). Approximately 24 h before transfection, the cells were seeded in a 96-well plate (30,000 cells per well) and cultured in medium. For transfection, Lipofectamine MessengerMAX transfection reagent (0.15 µL, Thermo Fishe Scientific) in Opti-MEM (6.25 µL, Thermo Fisher Scientific) was mixed with GLuc-mRNA (100 ng) in Opti-MEM (6.25 µL, Thermo Fisher Scientific). After 8 h at standard conditions, 10 µl of supernatant was removed and frozen until the luminescence measurement. The cell medium was replaced with fresh medium. After additional 16 h, the supernatant for the second timepoint was collected. Subsequently, the cells were irradiated at 365 nm for 10 s (1400 mA, 460 mW/cm^2) before the cell medium was replaced. The samples for the later timepoints were collected as described for the 8 h samples. Prior to the luminescence measurement, the samples were diluted 1:20 with phosphate-buffered saline (PBS). The diluted samples were transferred to a 96-well plate (5 µL per well). Using the Tecan Infinite M1000 PRO plate reader, 50 µL of the reaction buffer (PJK Reconstruction buffer and Coelenterazine, Gaussia-Juice Luciferase Assay kit) was added to the well and the luminescence activity was measured. For measurement an integration time of 3 s was used. The background of untreated cells was subtracted from all samples and values were normalized to m^{7}GpppG-capped mRNA.

Microscopy

HeLa cells (Merck, 93021013-1VL) were cultured as described above. Approximately 24 h before transfection, cells were seeded in a 96-well plate suitable for microscopy (10,000 cells per well). For transfection, Lipofectamine MessengerMAX transfection reagent (0.15 µL) in Opti-MEM (6.25 µL, Thermo Fisher Scientific) was mixed with eGFP-mRNA (40 ng) in Opti-MEM (6.25 µL, Thermo Fisher Scientific). After 24 h at standard conditions, the cells were irradiated (365 nm, 10 s,1400 mA, 460 mW) before

the cell medium was renewed. Another 24 h later the cells were washed twice with PBS, before fixing with paraformaldehyde (4% in PBS). After washing, cells were stained with 4′,6- diamidino-2-phenylindole (DAPI, 10 µg/mL in PBS). Imaging was performed using a Leica SP8 inverted confocal laser scanning microscope equipped with a 10x (air) and a 40x (oil immersion) objective. Images were analysed with ImageJ and LAS X software (Leica Microsystems). For z-stacks maximum intensity is shown.

2. Supplementary Tables

Table S1 Percentage of “caged” guanosine **7** after irradiation at the indicated wavelengths and durations.

		#1	#2	#3		
	Time [s]	Area [%]	Area [%]	Area [%]	Mean value	Standard deviation
**365 nm 140 mW/cm²**	10	15.6	17.1	12.3	15.0	2.04
	20	5.6	1.8	4.3	3.9	1.59
	30	3.1	0.0	0.0	1.0	1.46
**405 nm 140 mW/cm²**	30	22.9	21.8	22.0	22.2	0.51
	60	8.9	6.7	7.1	7.6	0.97
	90	4.4	3.1	2.2	3.3	0.93
**420 nm 56 mW/cm²**	60	60.7	45.0	57.6	54.4	6.80
	120	35.0	29.0	33.6	32.5	2.54
	180	26.4	19.1	22.0	22.5	3.00

Table S2 Decrease of “caged” guanosine **7** calculated from Table S1 and plotted in Figure 3

		#1	#2	#3		
	Time [s]	Decrease [%]	Decrease [%]	Decrease [%]	Mean value	Standard deviation
**365 nm 140 mW/cm²**	10	84.4	82.9	87.7	85.0	2.04
	20	10.0	15.4	7.9	11.1	3.15
	30	2.5	1.8	4.3	2.9	1.09
**405 nm 140 mW/cm²**	30	77.1	78.2	78.0	77.8	0.51
	60	14.0	15.1	14.9	14.7	0.45
	90	4.5	3.6	4.9	4.3	0.56
**420 nm 56 mW/cm²**	60	39.3	55.0	42.4	45.6	6.80
	120	25.8	16.0	24.0	21.9	4.27
	180	8.6	9.9	11.6	10.0	1.24

Table S3 Composition of the crude click mixture of azido-modified CyCliCap-HiBiT-mRNA.

	#1	#2	#3		
	Composition [%]	Composition [%]	Composition [%]	Mean value	Standard deviation
**Polymer**	20	25	15	20	4.0
**Circular**	34	23	48	35	10.2
**Trimer**	4	6	5	5	0.8
**Dimer**	9	12	6	9	2.4
**Monomer**	34	35	26	32	3.9

Table S4 Composition of the crude click mixture of azido-modified CyCliCap-GLuc-mRNA.

	**#1**	**#2**	**#3**		
	Composition [%]	Composition [%]	Composition [%]	Mean value	Standard deviation
**Polymer**	26	28	36	30	4.4
**Circular**	16	13	12	14	2.0
**Trimer**	2	3	3	3	0.7
**Dimer**	12	9	10	10	1.4
**Monomer**	44	47	39	44	3.2

Table S5 Composition of the crude click mixture of azido-modified CyCliCap-eGFP-mRNA.

	**#1**	**#2**	**#3**		
	Composition [%]	Composition [%]	Composition [%]	Mean value	Standard deviation
**Polymer**	21	22	24	22	1.1
**Circular**	16	15	9	13	3.1
**Trimer**	4	5	4	5	0.6
**Dimer**	13	11	19	15	3.4
**Monomer**	46	46	44	45	1.2

3. Supplementary Figures

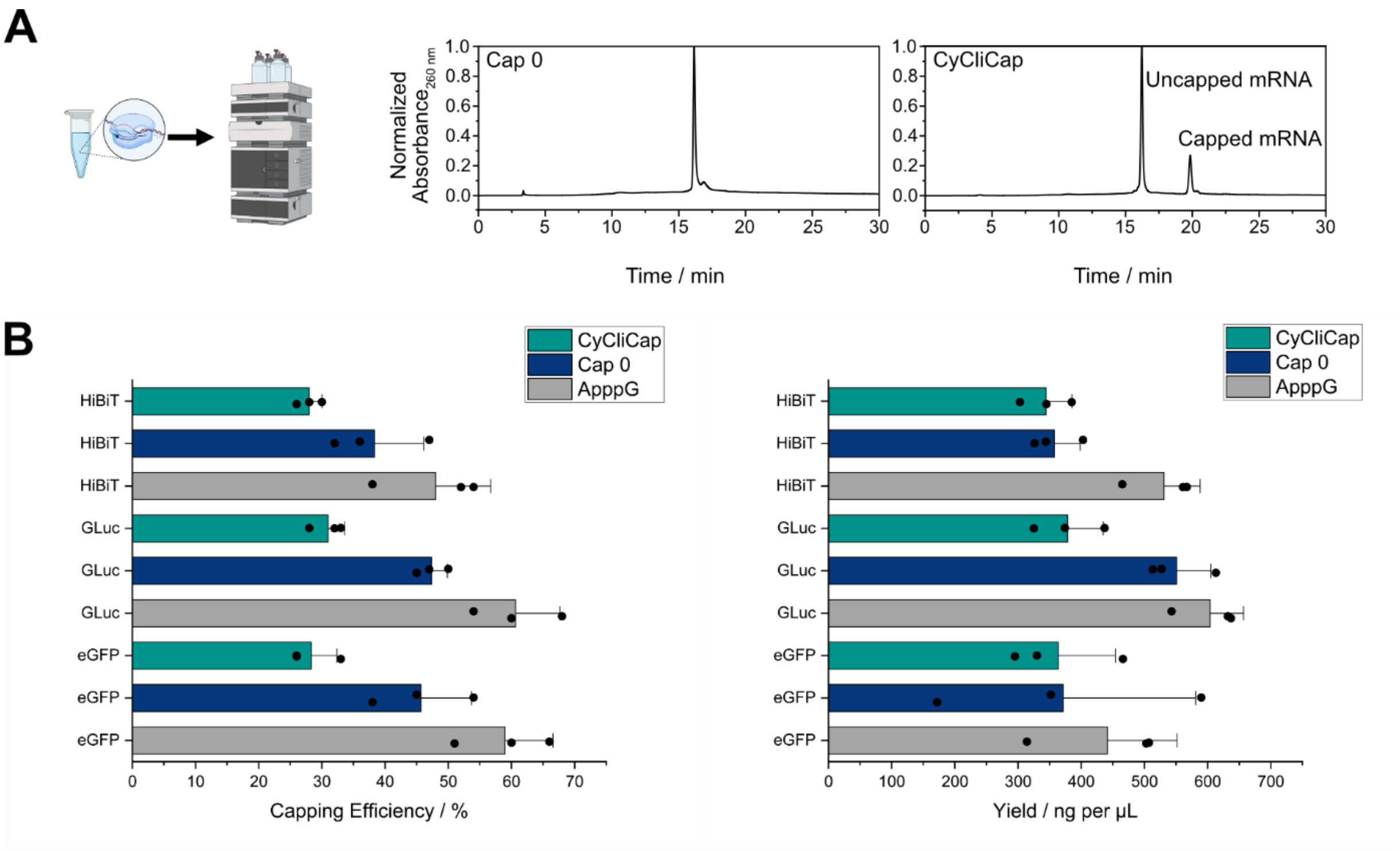

Figure S1 (A) HPLC purification of in vitro transcription producing GLuc-mRNA with Cap0 or CyCliCap. (B) Capping efficiency and yield of prepared HiBiT, GLuc and eGFP-mRNAs capped with CyCliCap, Cap 0 or ApppG cap. The capping efficiency for cap 0 and ApppG mRNAs was calculated as the amount of capped mRNA obtained after enzymatic digestion divided by the amount of total mRNA obtained after IVT. The capping efficiency of CyCliCap mRNA was determined from the HPLC chromatogram of the IVT. Data of n = 3 independent experiments is shown.

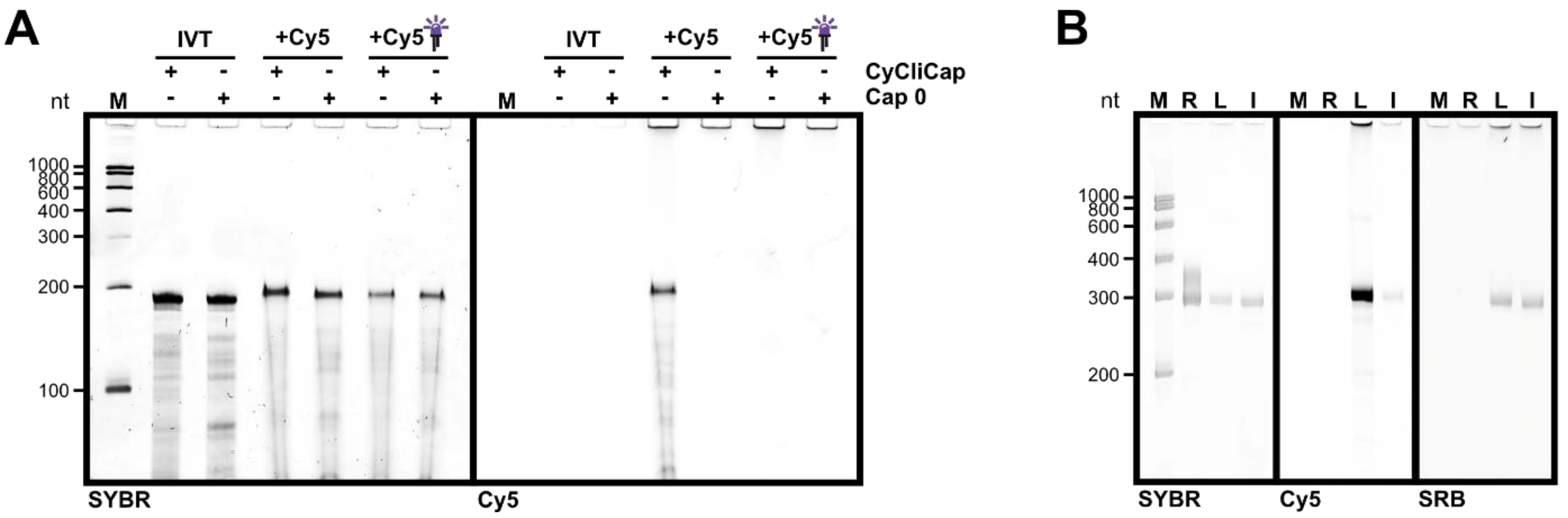

Figure S2 Labeling of RNA. (A) Cap-labeling of 185 nt-RNA. Short RNA was in vitro transcribed (IVT) in presence of CyCliCap or Cap 0 as negative control. After digestion of uncapped RNA, azido-sulfo-Cy5 was clicked (+Cy5). One batch was stored in dark and one batch was irradiated (365 nm, 30 s, 460 mW/cm^2). Only Cy5 clicked CyCliCap-RNA shows a band in the Cy5 channel, which disappears upon irradiation. The negative control Cap 0-RNA shows no signal in Cy5 channel (B) Dual-labeling of HiBiT-mRNA. CyCliCap HiBiT-mRNA was 3′ end modified with azido-ATP (reference R), and clicked with DBCO-SRB via SPAAC as well as azido-sulfo-Cy5 via CuAAC (labeled L). The dual-labeled HiBiT-mRNA (L) was irradiated (I) at 365 nm (460 mW/cm^2) for 30 s . The dual-labeled HiBiT-mRNA shows an intensive band in the Cy5 channel before irradiation, which significantly decreases after irradiation indicating successful labeling and deprotection. The 3′ end SRB labeling remains intact after irradiation.

DBCO-PEG$_4$-5/6-Sulforhodamine B

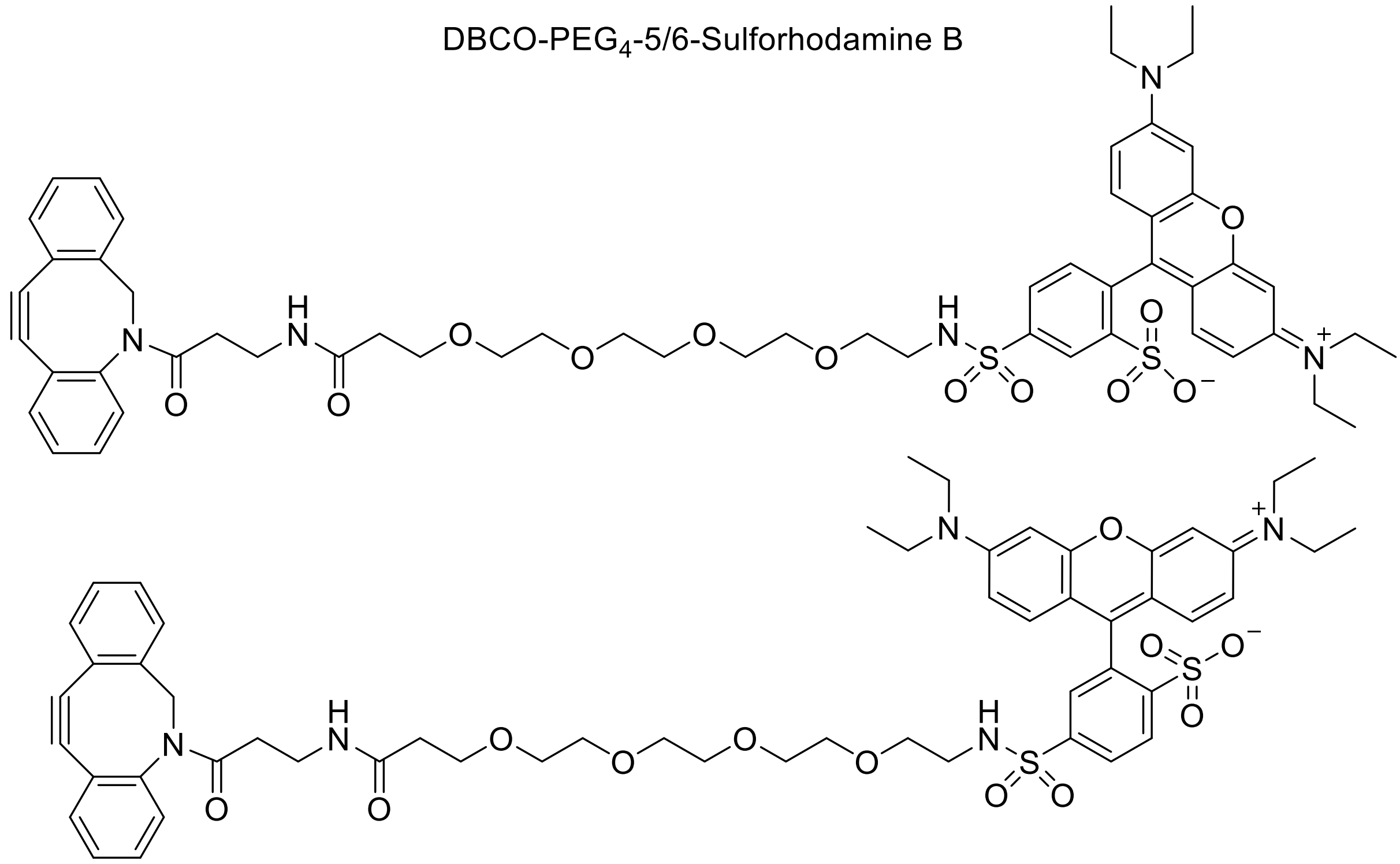

Sulfo-Cy5-Azide

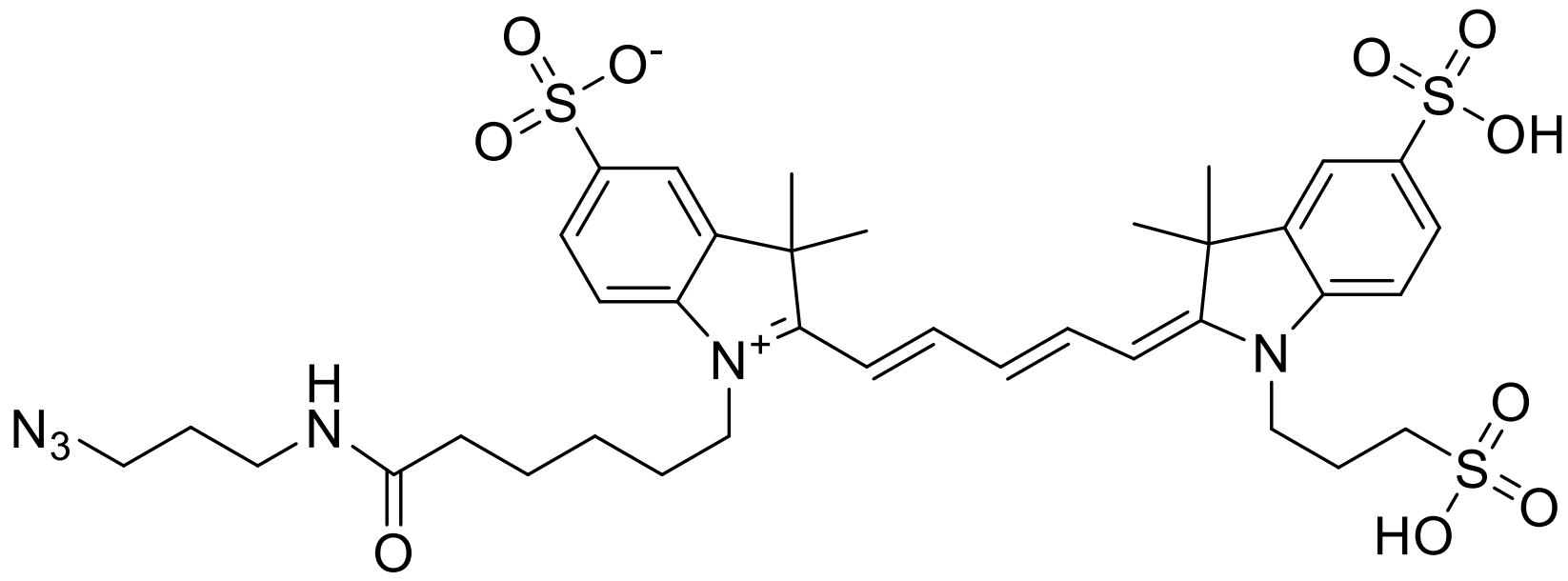

Figure S3 Structures of the used fluorophores.

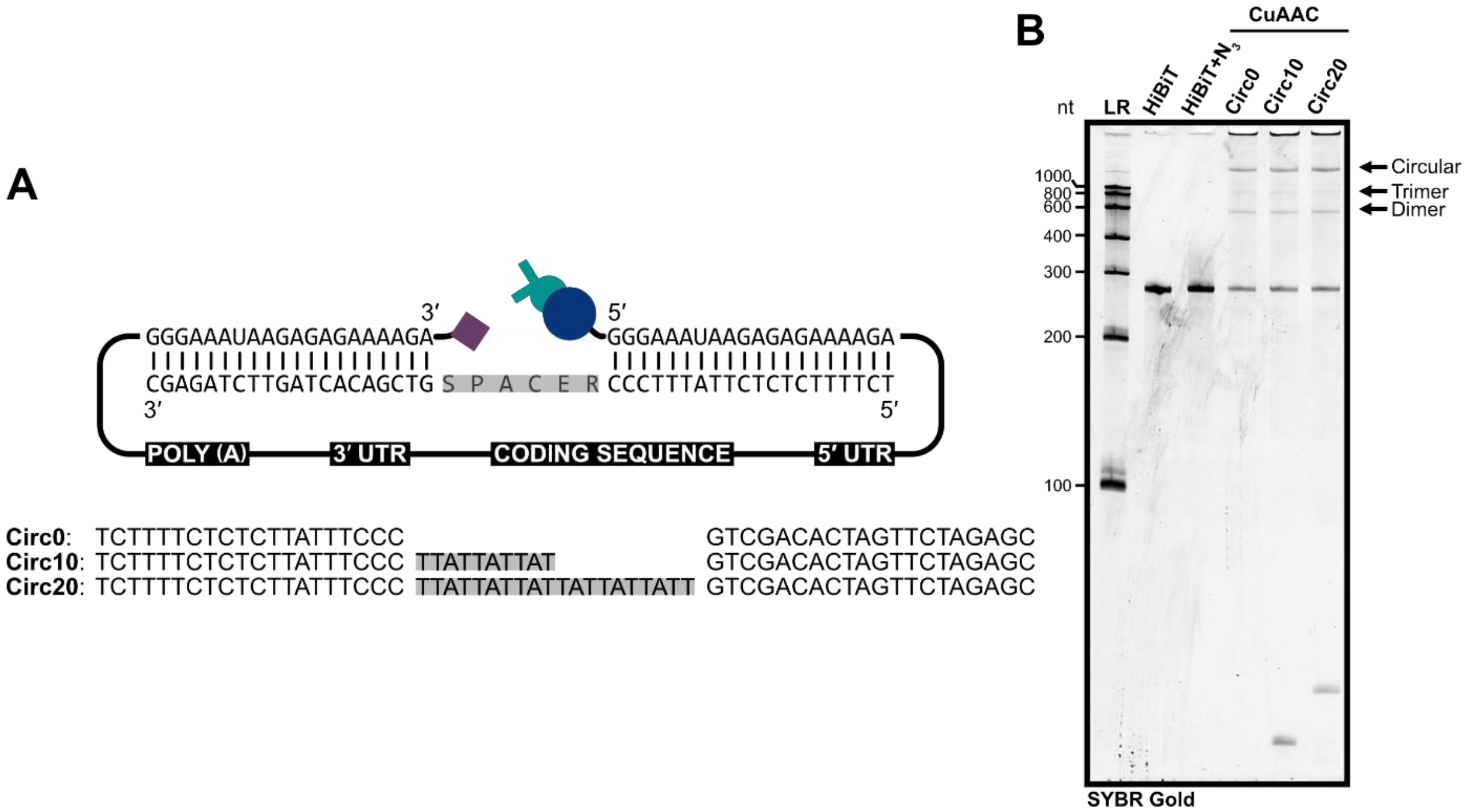

Figure S4 DNA splint design. (A) Three different splints were designed. They hybridize with the first 20 and last 20 encoded nucleotides of the mRNA. As the 3′ end of in vitro transcribed mRNA is not uniform, 10 or 20 bases (Circ10 or Circ20) were introduced as spacer. (B) Analysis of the CuAAC click ligation reaction using azido-modified CycliCap-HiBiT-mRNA with annealed DNA splints. Since all DNA splints perform equally good, Circ0 without a spacer sequence was eventually used for mRNA circularization. LR: RiboRuler Low Range RNA ladder. HiBiT: HiBit-mRNA with CyCliCap. HiBiT+N_3: Azido-modified CyCliCap-HiBiT-mRNA, which was used in the CuAAC click reactions

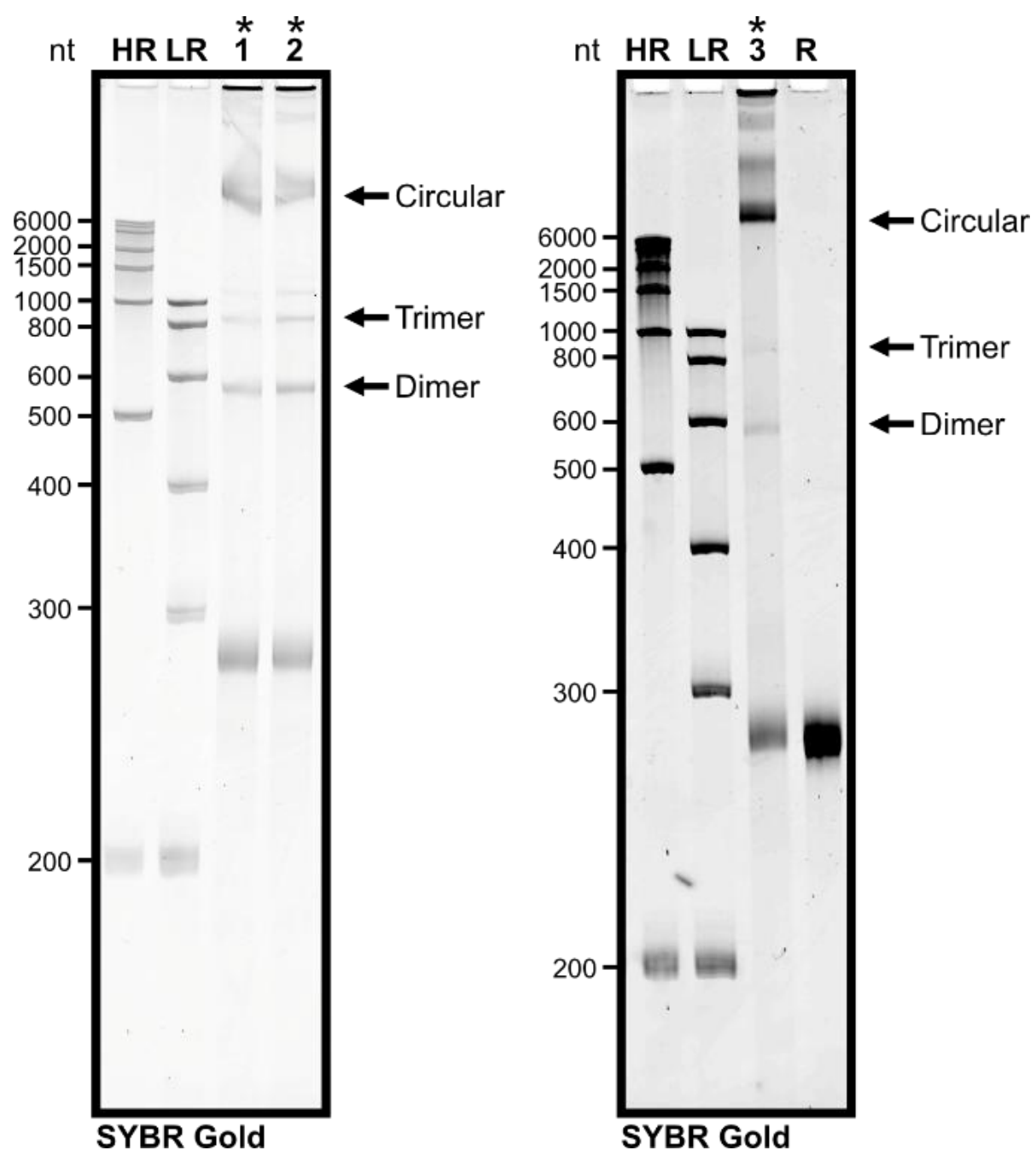

Figure S5 Analysis of crude circular HiBiT mRNA on denaturing polyacrylamide gels. The lanes indicated with a * were used for quantification of the composition of the crude click mixture using Image J. HR: RiboRuler High Range RNA ladder. LR: RiboRuler Low Range RNA ladder. Reference R: azido-modified linear CyCliCap-HiBiT-mRNA. See Table S3 for the ImageJ analysis.

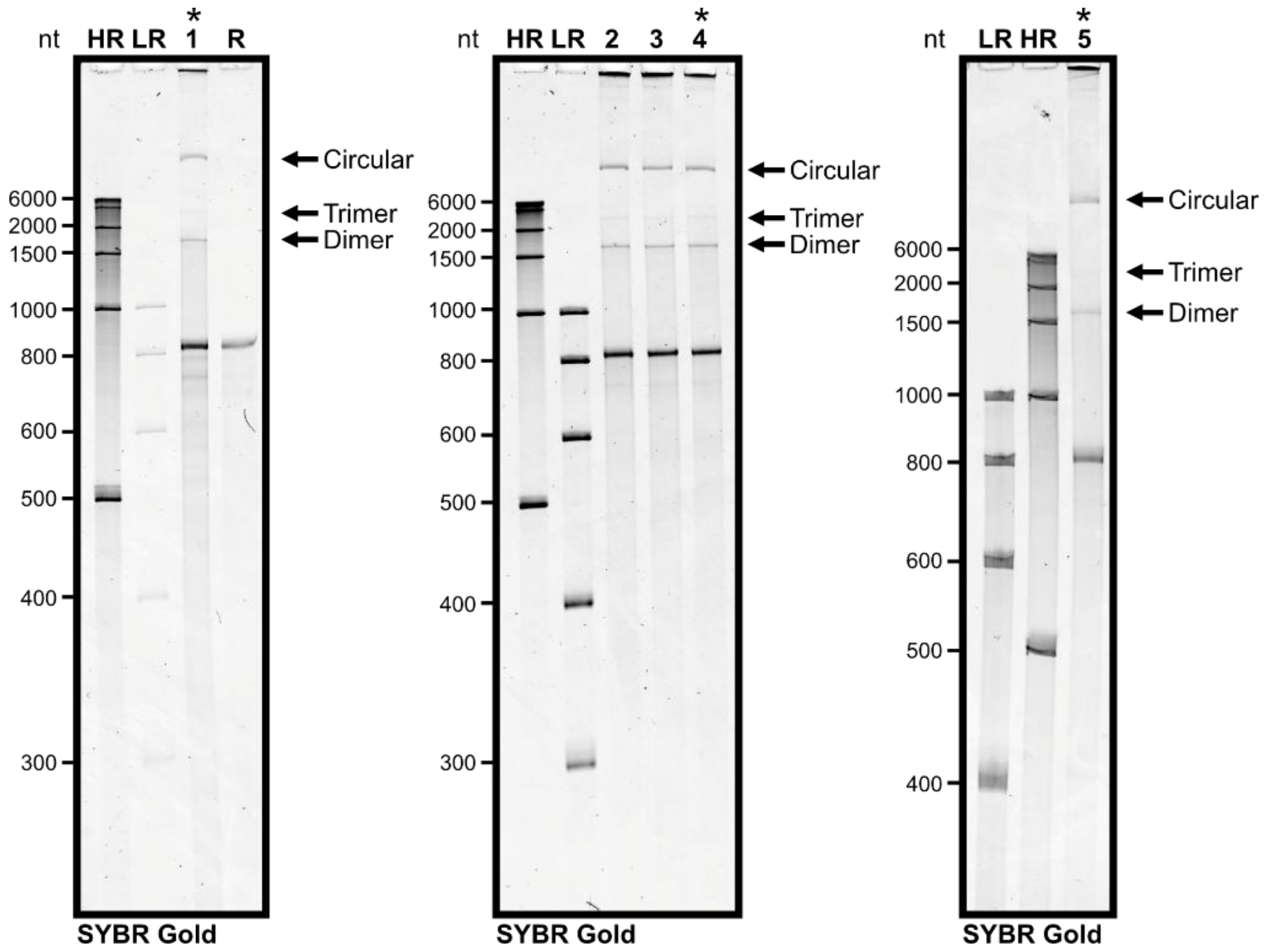

Figure S6 Analysis of crude circular GLuc mRNA on denaturing polyacrylamide gels. The lanes indicated with a * were used for quantification of the composition of the crude click mixture using Image J. HR: RiboRuler High Range RNA ladder. LR: RiboRuler Low Range RNA ladder. Reference R: azido-modified linear CyCliCap-GLuc-mRNA. See Table S4 for the ImageJ analysis.

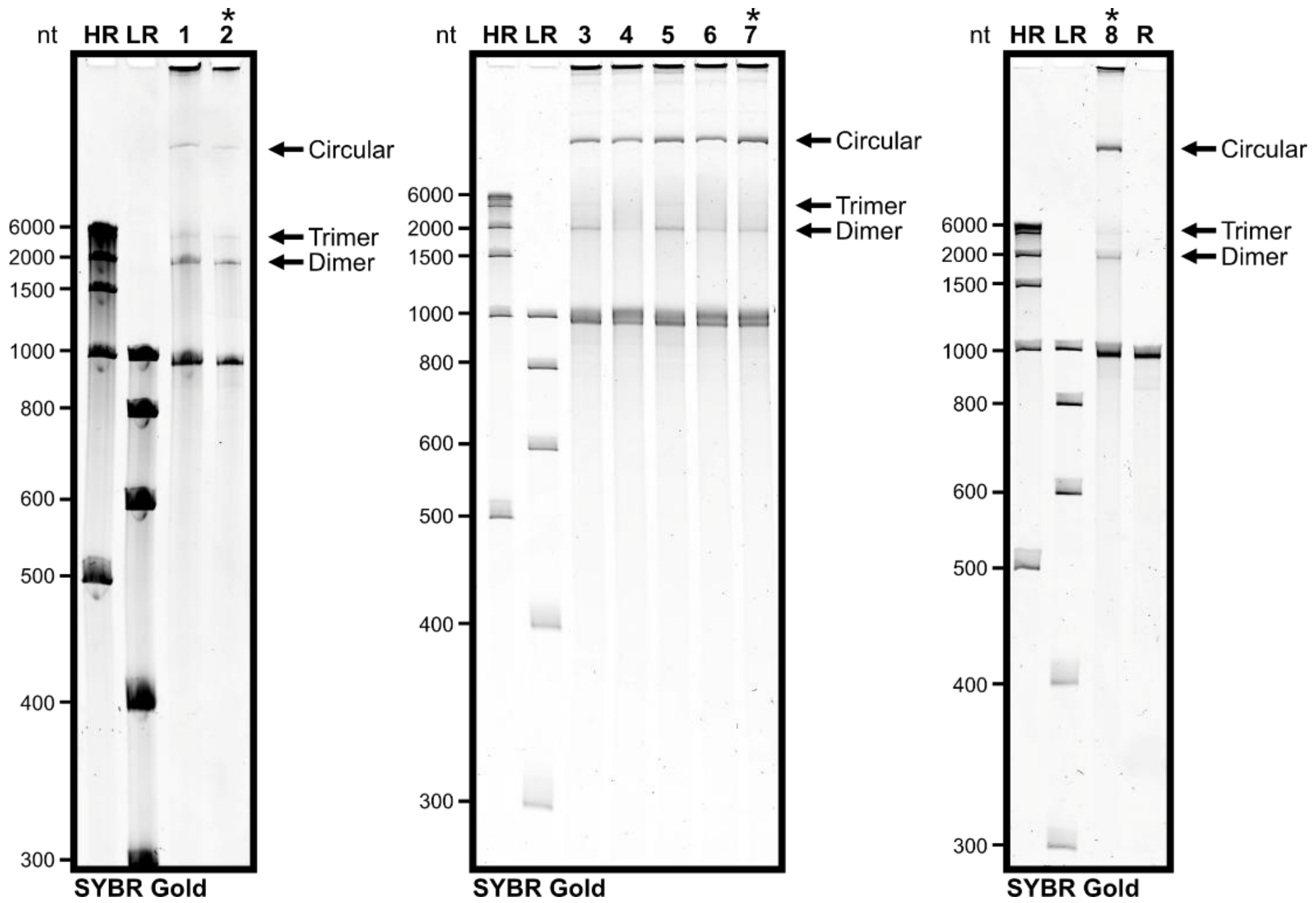

Figure S7 Analysis of crude circular GLuc-mRNA on denaturing polyacrylamide gels. The lanes indicated with a * were used for quantification of the composition of the crude click mixture using Image J. HR: RiboRuler High Range RNA ladder. LR: RiboRuler Low Range RNA ladder. Reference R: azido-modified linear CyCliCap-eGFP-mRNA. See Table S5 for the ImageJ analysis.

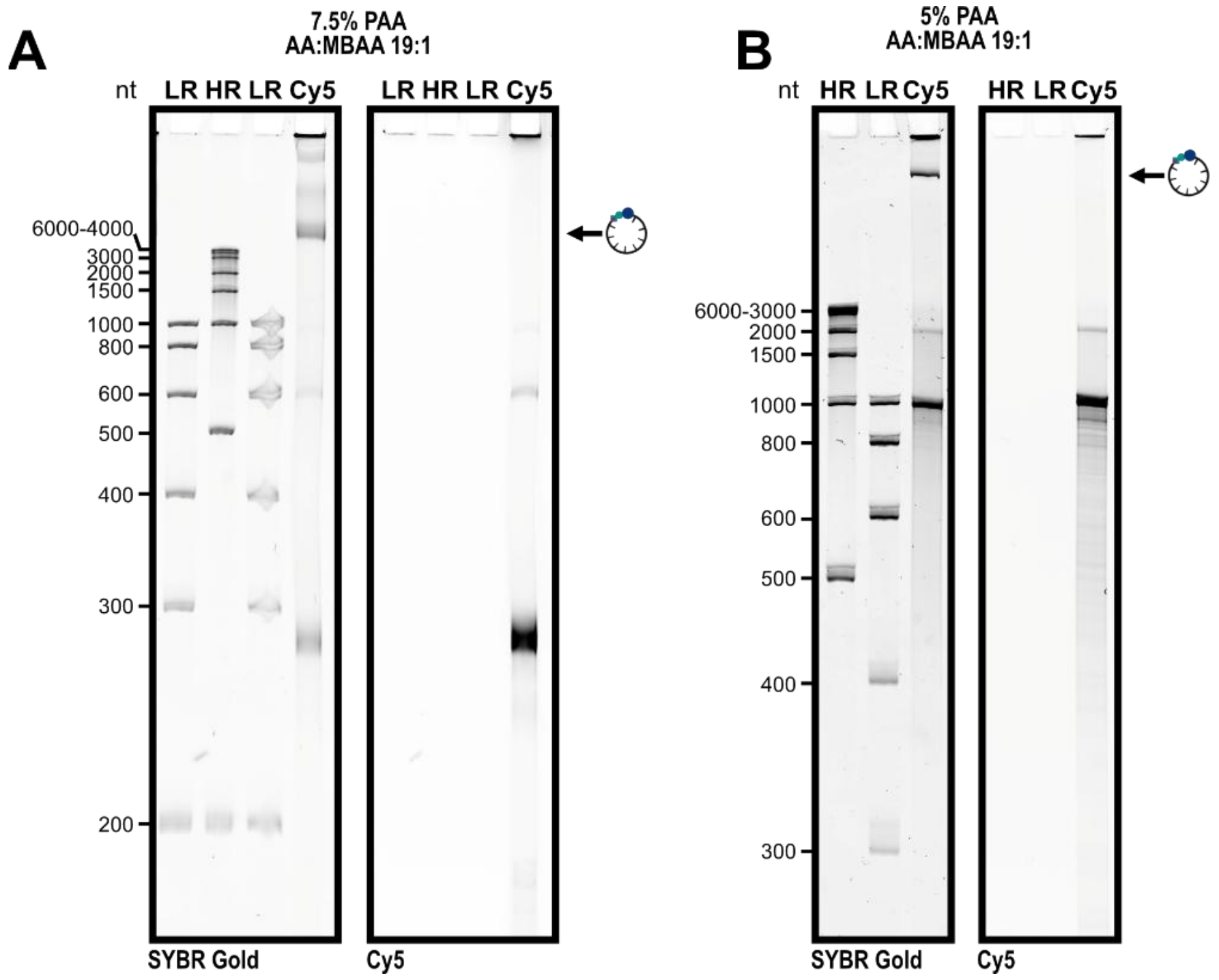

Figure S8 Labeling of crude circular mRNA mixture with azido-sulfo-Cy5. Successful Cy5 labeling seen in Cy5 channel indicates the presence of linear CyCliCap mRNA species. The lack hereof confirms circularity of the corresponding band seen in SYBR Gold channel (A) HiBiT-mRNA. (B) eGFP-mRNA.

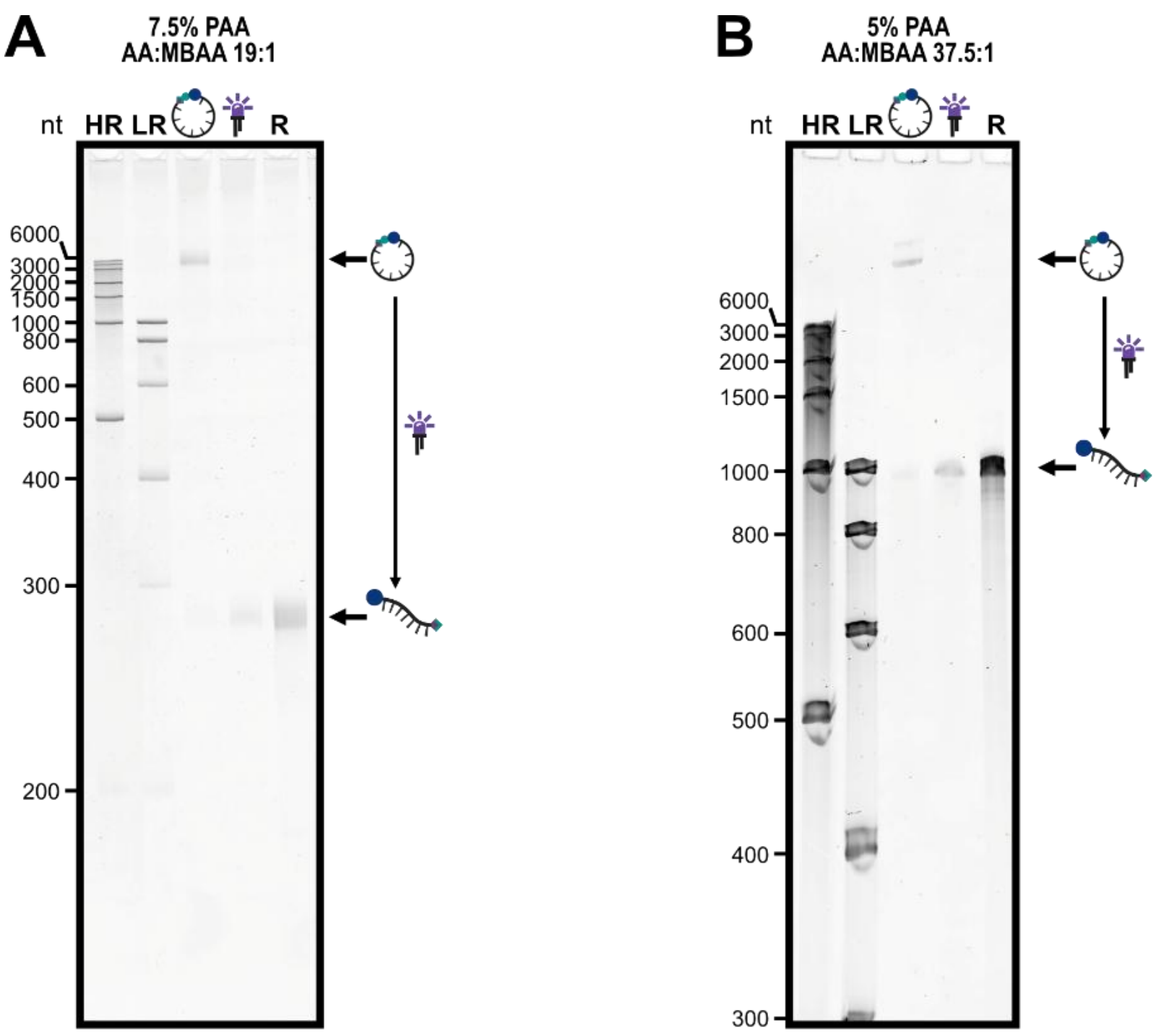

Figure S9 In vitro linearization of circularized mRNA. (A) Isolated circular HiBiT-mRNA was irradiated at 365 nm (460 mW/cm^2) for 30 s. Linear, azido-modified HiBiT-mRNA used for circularization was loaded as reference (R). (B) Isolated circular eGFP-mRNA was irradiated at 365 nm (460 mW/cm^2) for 30 s. Linear, azido-modified eGFP-mRNA used for circularization was loaded as reference (R).

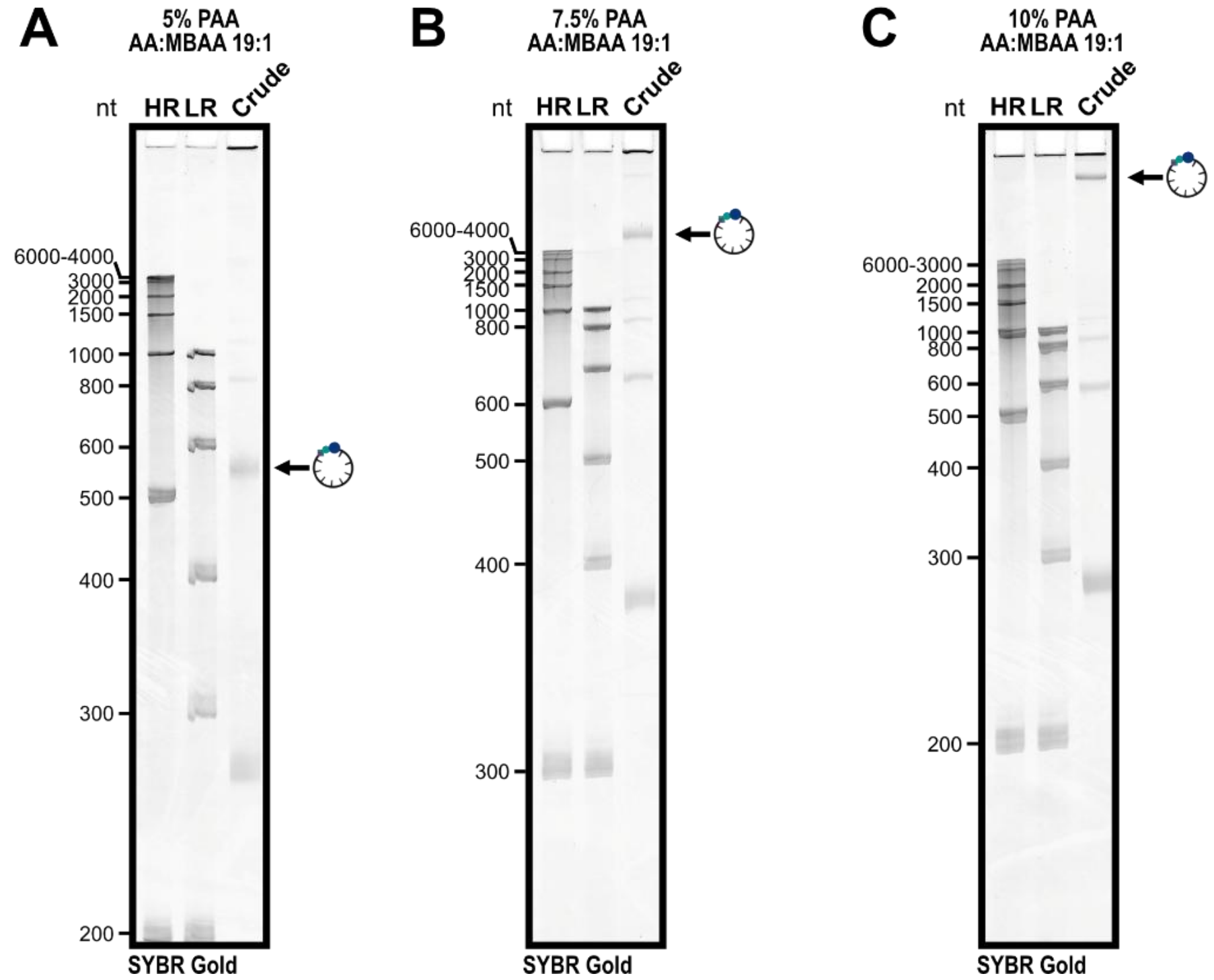

Figure S10 Gel shift assay with crude circular HiBiT-mRNA on denaturing polyacrylamide gels with 19:1 acrylamide to methylenebisacrylamide ratio. The arrow indicates the circular mRNA. (A) Analysis on 5 % gel. Separation of linear and circular HiBiT-mRNA is not obtained. (B) Analysis on 7.5 % gel. (C) Analysis on 10 % gel.

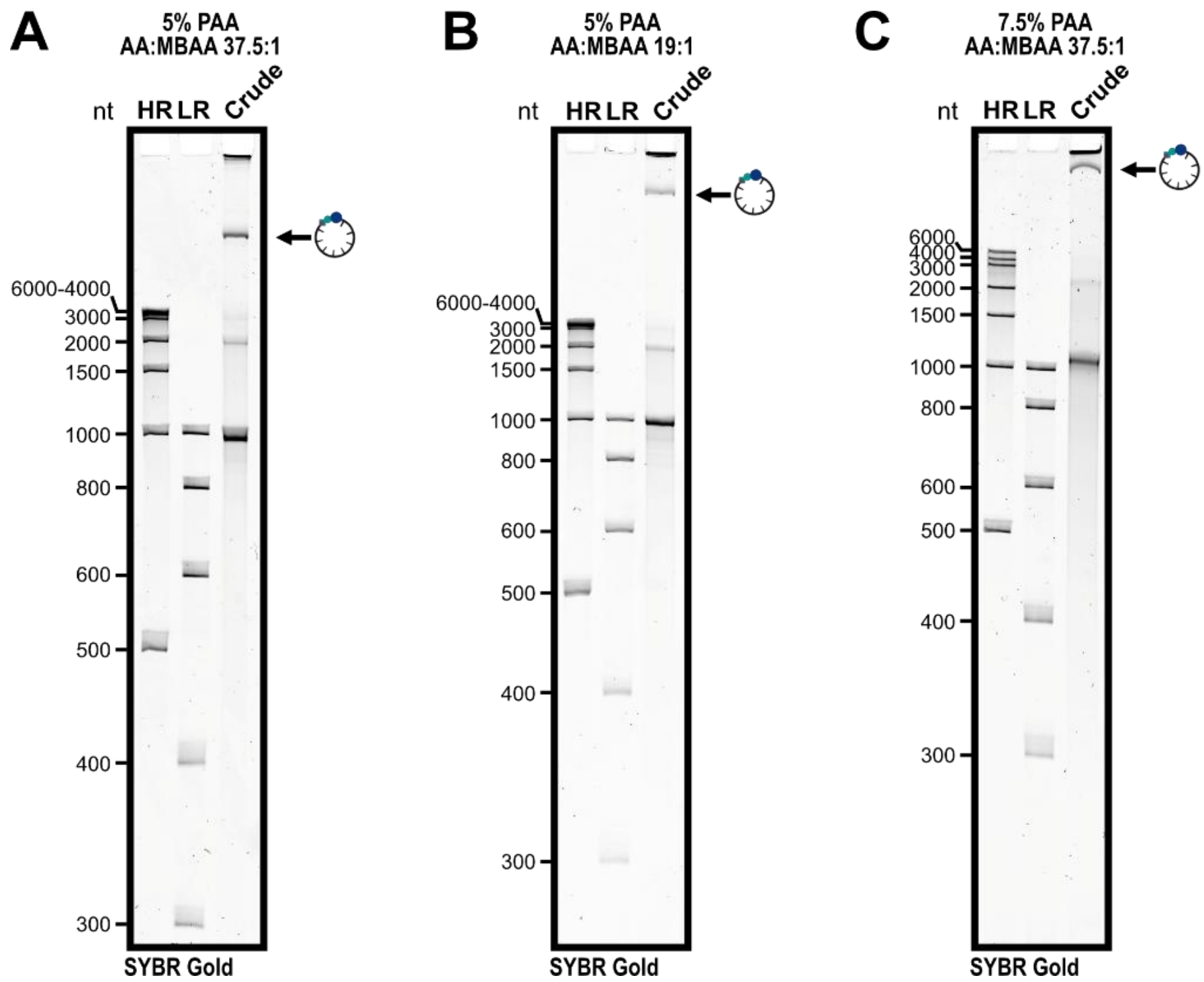

Figure S11 Gel shift assay with crude circular eGFP-mRNA. The arrow indicates the circular mRNA. (A) Analysis on 5 % dPAA gel with 37.5:1 acrylamide to methylenebisacrylamide ratio. (B) Analysis on 5 % dPAA gel with 19:1 acrylamide to methylenebisacrylamide ratio. (C) Analysis on 7.5 % gel with 37.5:1 acrylamide to methylenebisacrylamide ratio.

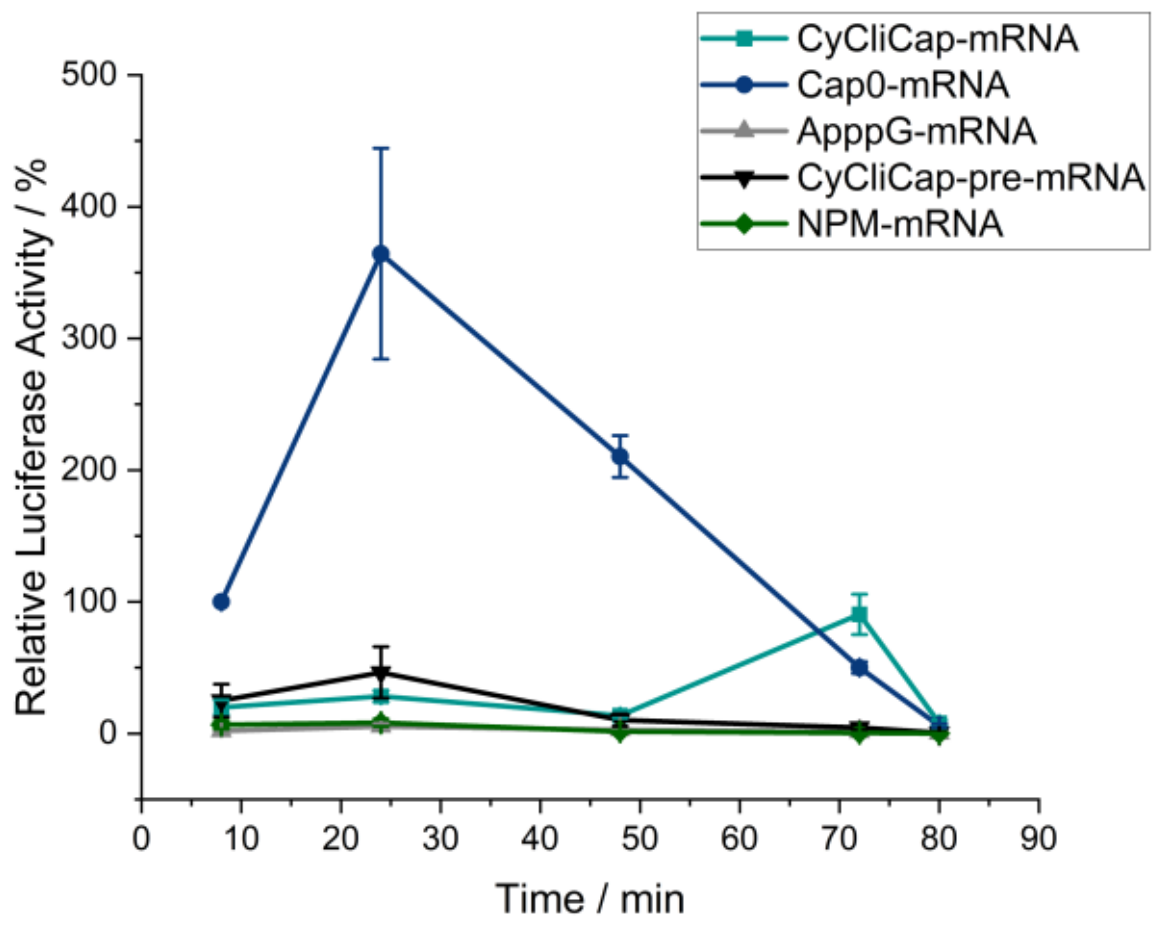

Figure S12 Relative protein output from HeLa cells transfected with differently capped GLuc-mRNAs. Cells were irradiated (365 nm, 10 s, 460 mW/cm^2) 56 h after transfection. Luciferase assay was performed at indicated timepoints until 80 h post transfection. Mean values and standard deviation of $n = 3$ independent experiments are shown. All values are normalized to unirradiated Cap0-GLuc-mRNA at 8h post transfection (=100%).

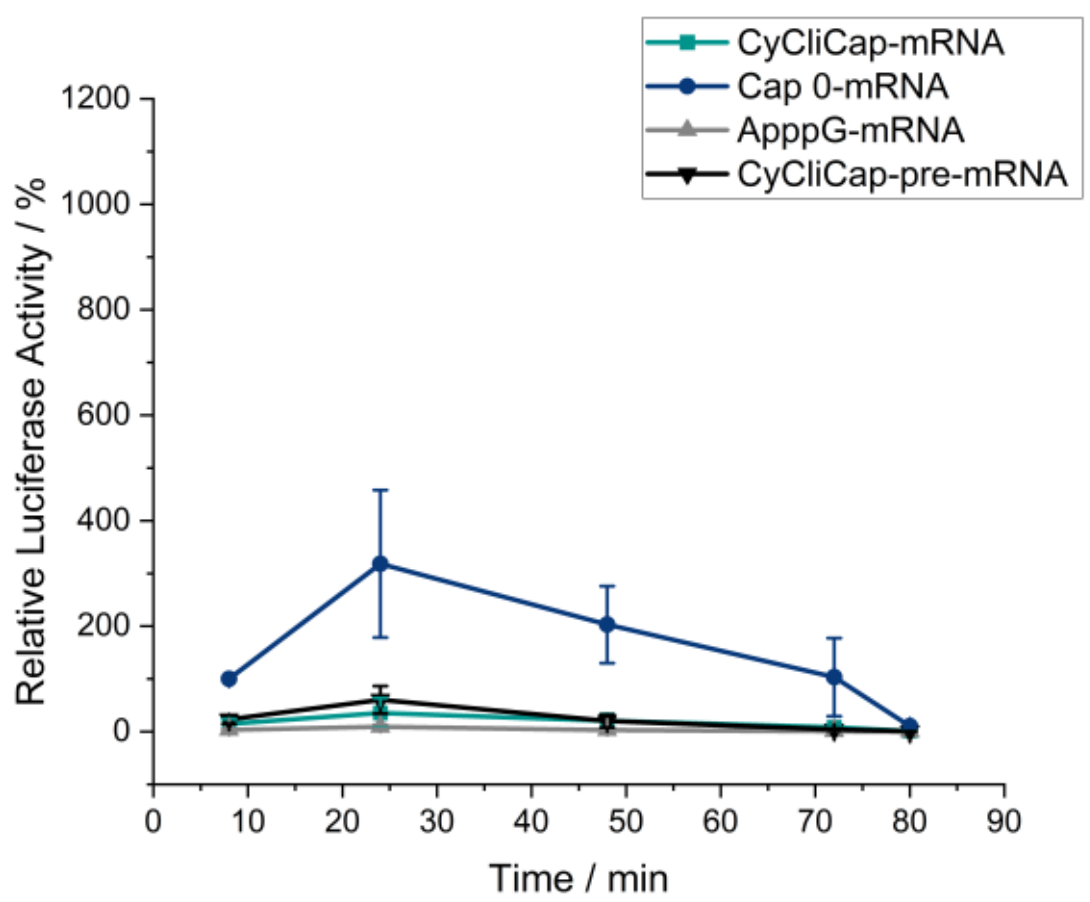

Figure S13 Relative protein output from HeLa cells transfected with differently capped GLuc-mRNAs. Cells were kept in dark for 80 h. Mean values and standard deviation of $n = 3$ independent experiments are shown. The 80 h time-point is $n = 2$ independent replicates. All values are normalized to unirradiated cap0-GLuc-mRNA at 8 h post transfection (=100%).

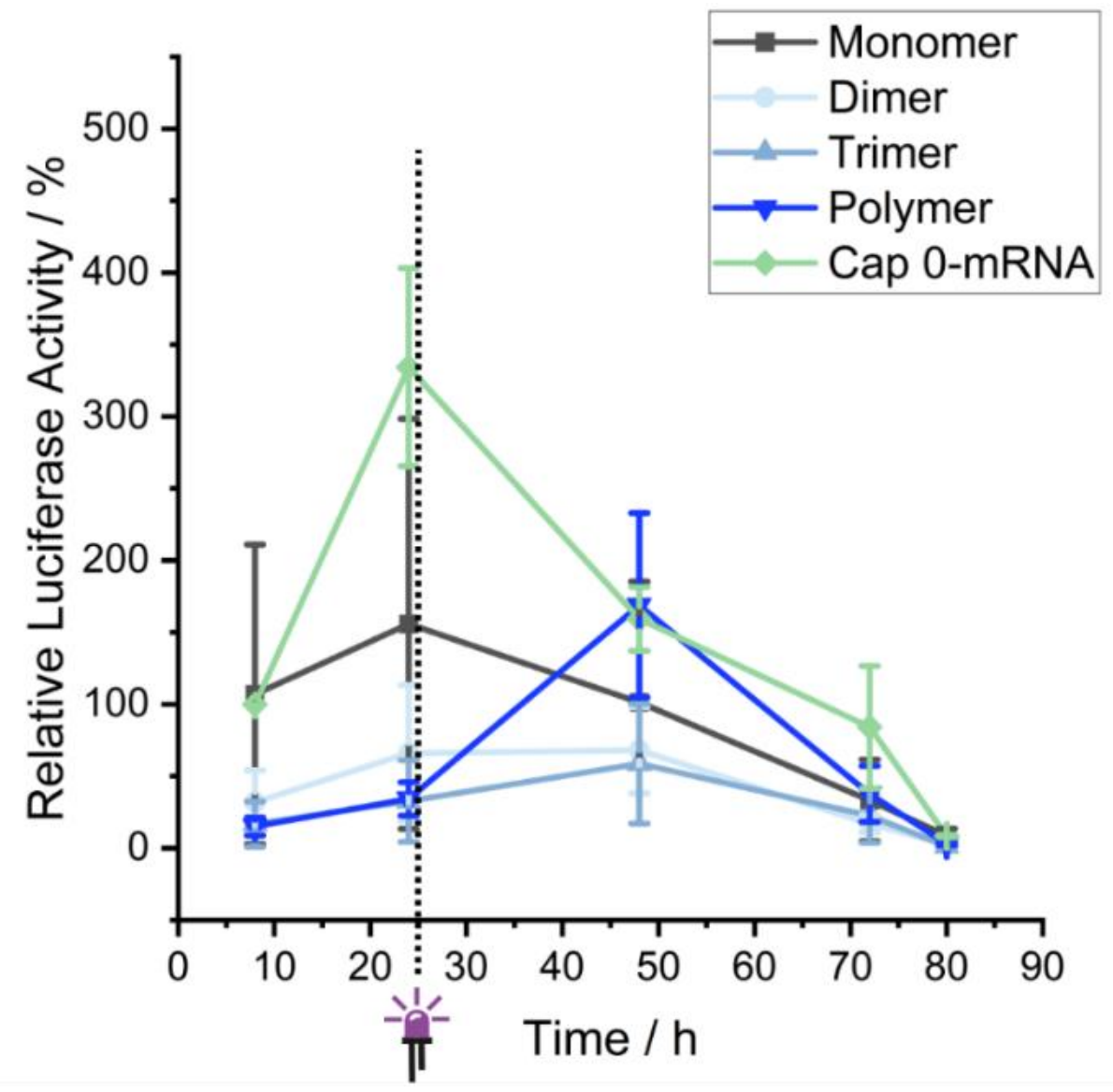

Figure S14 Relative protein output from HeLa cells transfected with differently capped GLuc-mRNAs. Cap 0-mRNA: with Cap0 and linear (=canonical); Monomer / Dimer / Trimer: Gel-purified side-products of the CuAAC with defined lengths; Polymer: Undefined mix of large gel-purified side-product of the CuAAC. Cells were irradiated (10 s, 365 nm, 460 mW/cm^2) at 24 h post transfection. Mean values and standard deviation of $n = 3$ independent experiments are shown. The 80 h time-point is n = 2 independent replicates.

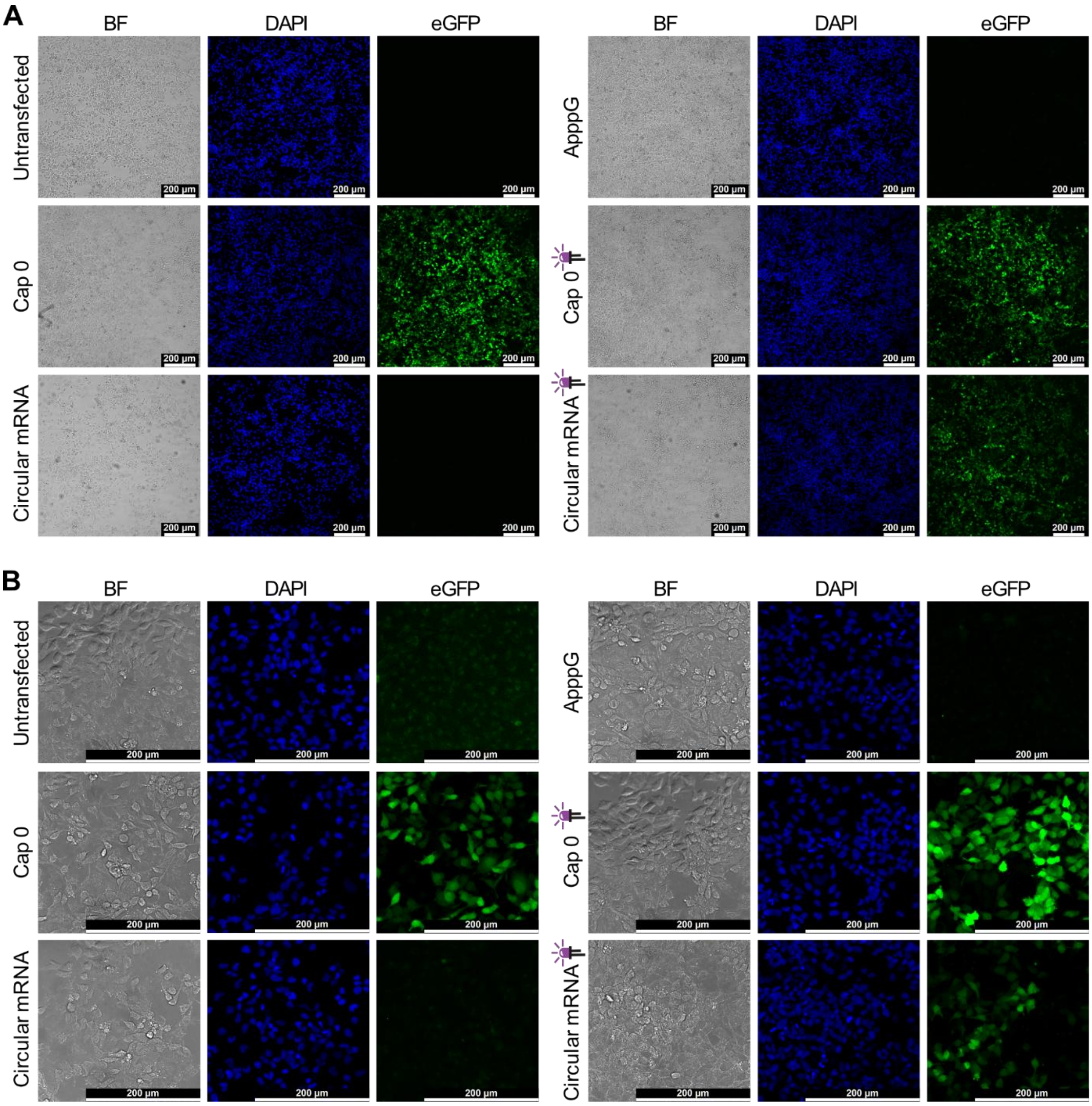

Figure S15 Replicate 1 of fluorescence confocal microscopy images of HeLa cells transfected with circular CyCliCap-eGFP mRNA or control mRNAs. Cells were either kept in dark or irradiated (365 nm, 10 s, 460 mW/cm^2) at 24 h post transfection (indicated by LED icon). Cap0-eGFP-mRNA serves as a positive control. Samples were imaged for eGFP fluorescence 48 h after transfection. (A) 10x air objective. (B) 40x oil immersion objective. BF: Bright-field. DAPI: 4′,6- diamidino-2-phenylindole.

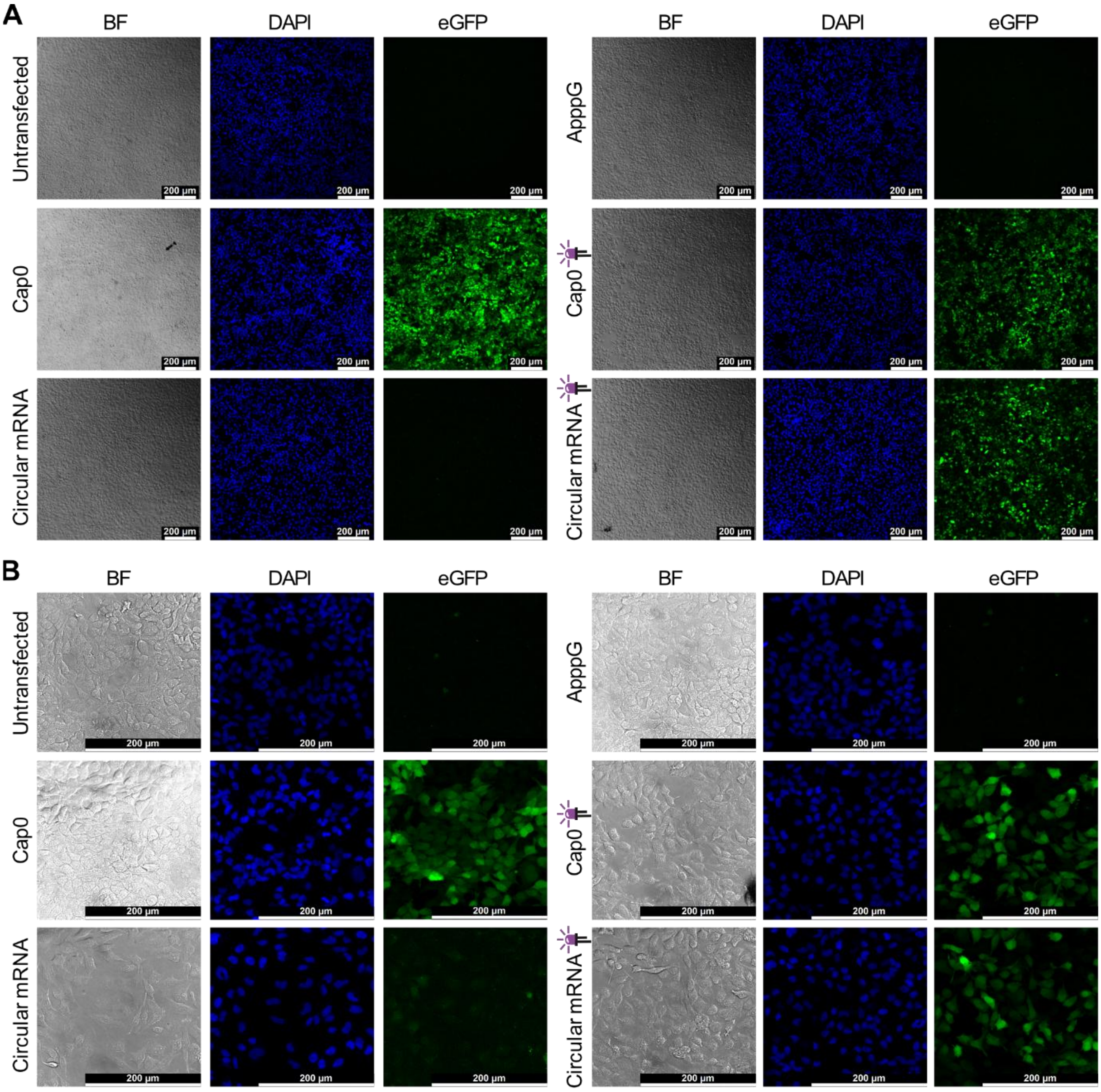

Figure S16 Replicate 2 of fluorescence confocal microscopy images of HeLa cells transfected with circular CyCliCap-eGFP mRNA or control mRNAs. Cells were either kept in the dark or irradiated (365 nm, 10 s, 460 mW/cm^2) at 24 h after transfection (indicated by LED icon). Cap0-eGFP-mRNA serves as a positive control. Samples were imaged for eGFP fluorescence 48 h after transfection. (A) 10x air objective. (B) 40x oil immersion objective. BF: Bright-field. DAPI: 4′,6- diamidino-2-phenylindole.

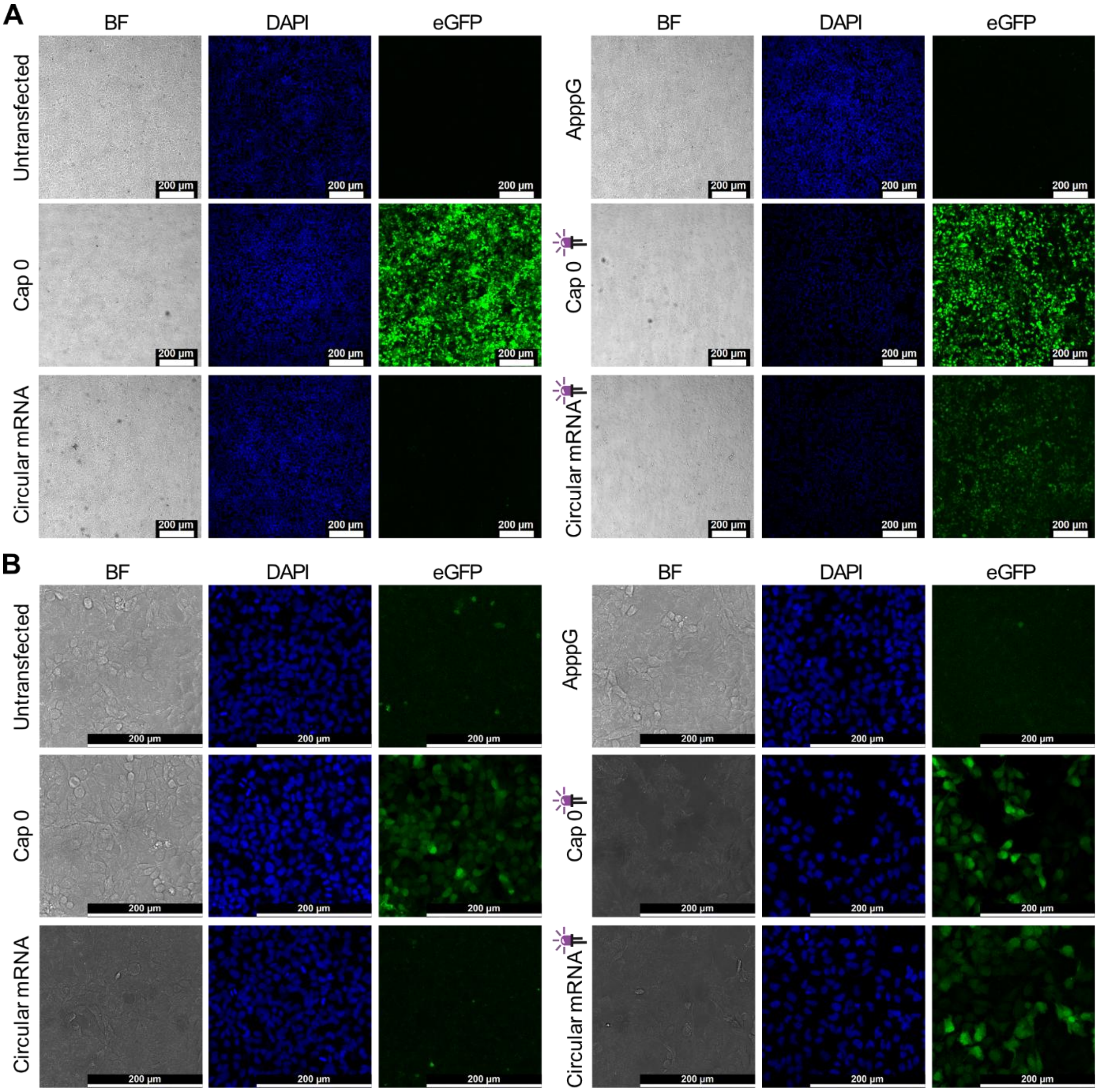

Figure S17 Replicate 3 of fluorescence confocal microscopy images of HeLa cells transfected with circular CyCliCap-eGFP mRNA or control mRNAs. Cells were either kept in the dark or irradiated (365 nm, 10 s, 460 mW/cm^2) at 24 h after transfection (indicated by LED icon). Cap0-eGFP-mRNA serves as a positive control. Samples were imaged for eGFP fluorescence 48 h after transfection. (A) 10x air objective. (B) 40x oil immersion objective. BF: Bright-field. DAPI: 4′,6- diamidino-2-phenylindole.

4. Chemical Synthesis

All synthesis steps were performed under dark lab conditions using red light as sole light source.

1-(6-Nitrobenzo[*d*][1,3]dioxol-5-yl)ethan-1-ol (3)

The synthesis of compound **3** was adapted from Teague et al.[42] A solution of trimethylaluminium (2 M in toluene; 25 mL, 50 mmol, 2.00 eq.) was added dropwise to an ice cold solution of 6-nitropiperonal (5.03 g, 25.8 mmol, 1.00 eq.) in dry dichloromethane (200 mL). The reaction mixture was stirred for 2 h until full conversion and the reaction was carefully quenched with NaOH (1 M, 200 mL). The aqueous phase was extracted with dichloromethane (2 x 150 mL). The combined organic phases were washed with ddH_2O (2 x 200 mL). After removal of the solvent under reduced pressure, the raw material was purified via dry load on PuriFlash XS530 (PF-50SIHP-F0330 column, eluent 80 : 20 cyclohexane : dichloromethane to 55 : 40 : 5 cyclohexane : dichloromethane : methanol in 4 CV). The solvent was evaporated under reduced pressure to obtain compound **3** as orange solid (4.36 g, 20.6 mmol, 80% yield).

^{1}H-NMR (400 MHz, DMSO-*d*$_6$): δ 7.52 (s, 1H, H-6), 7.27 (s, 1H, H-3), 6.21 (dd, *J* = 11.0, 0.7 Hz, 2H, H-1), 5.49 (d, *J* = 4.4 Hz, 1H, 8-OH), 5.18 – 5.11 (m, 1H, H-8), 1.34 (d, *J* = 6.3 Hz, 3H, H-9).

^{13}C-NMR (101 MHz, DMSO-*d*$_6$): δ 151.87 (C-2), 146.35 (C-7), 140.65 (C-5), 140.25 (C-4), 106.08 (C-3), 104.28 (C-6), 103.17 (C-1), 64.00 (C-8), 25.11 (C-9).

HRMS (m/z): $[M+Na]^+$ calculated for $C_9H_9N_1O_5Na$ 234.03729, found 234.03722.

4-(1-Hydroxyethyl)-2-methoxy-5-nitrophenol (4)

A published procedure was adapted for the synthesis of compound **4**.[1] Sodium methoxide (4.70 g, 87.0 mmol, 6.00 eq.) was added to compound **2** (3.06 g, 14.5 mmol, 1.00 eq.) in dry dimethyl sulfoxide (43 mL) and dry methanol (22 mL) and stirred overnight at 100 °C until full conversion. The reaction mixture was diluted with ddH_2O (150 mL), neutralized with HCl and extracted with ethyl acetate (4 x 200 mL). The organic solvents were removed under reduced pressure and the raw material was purified via dry load on PuriFlash XS530 (PF-50SIHP-F0330 column, eluent 60 : 40 cyclohexane : ethyl acetate to 55 : 36 : 9 cyclohexane : ethyl acetate : methanol in 4 CV). The solvent was evaporated under reduced pressure to obtain compound **4** as a pale yellow solid (2.17 g, 10.2 mmol, 75% yield).

^{1}H-NMR (400 MHz, DMSO-*d*$_6$): δ 9.93 (s, 1H, 7-OH), 7.40 (s, 1H, H-6), 7.31 (s, 1H, H-3), 5.41 (d, *J* = 4.2 Hz, 1H, 8-OH), 5.27 – 5.20 (m, 1H, 1H, H-8), 3.89 (s, 3H, H-1), 1.35 (d, *J* = 6.2 Hz, 3H, H-9).

^{13}C-NMR (101 MHz, DMSO-*d$_6$*): δ 152.61 (C-2), 145.12 (C-7), 138.87 (C-5), 136.52 (C-4), 110.66 (C-3), 109.27 (C-6), 63.91 (C-8), 55.97 (C-1), 25.26 (C-9).

HRMS (m/z): $[M+Na]^+$ calculated for $C_9H_{11}N_1O_5Na$ 236.05294, found 236.05287.

1-(4-((Propargyl)oxy)-5-methoxy-2-nitrophenyl)ethan-1-ol (5)

Potassium carbonate (2.46 g, 17.8 mmol, 1.1 eq.) and propargyl bromide (1.73 mL, 2.31 g, 19.4 mmol, 1.2 eq.) were added to a solution of compound **4** (3.44 g, 16.2 mmol, 1.0 eq.) in DMF (20 mL) and stirred for 5 h at room temperature. Subsequently, the reaction mixture was diluted with ddH_2O (100 mL) and saturated NH_4Cl solution (100 mL) and extracted with ethyl acetate (3 x 200 mL). The organic phases were combined and washed with HCl (aq., 0.25 M, 200 mL), brine (2x 100 mL) and ddH_2O (2 x 200 mL). The solvent was removed under reduced pressure and the raw material was purified via dry load on PuriFlash XS530 (PF-50SIHP-F0330 column, eluent 60 : 40 cyclohexane : ethyl acetate). The solvent was evaporated under reduced pressure to obtain compound **5** as a yellow solid (3.67 g, 14.6 mmol, 90% yield).

^{1}H-NMR (599 MHz, DMSO-*d$_6$*): δ 7.67 (s, 1H, H-5), 7.39 (s, 1H, H-8), 5.49 (d, *J*(12,12-OH) = 4.4 Hz, 1H, 12-O<u>H</u>), 5.27 (dd, *J* (11,12) = 6.2, *J* (11,12-OH) = 4.3 Hz, 1H, H-12), 4.92 (d, *J* (1,3)= 2.4 Hz, 2H, H-3), 3.91 (s, 3H, H-10), 3.63 (t, *J* (1,3)= 2.4 Hz, 1H, H-1), 1.38 (d, *J* (11,12)= 6.2 Hz, 3H, H-11).

^{13}C-NMR (151 MHz, DMSO-*d$_6$*): δ 153.62 (C-9), 144.62 (C-4), 138.94 (C-7), 138.65 (C-6), 109.58 (C-5), 109.36 (C-8), 79.04 (C-1), 78.50 (C-2), 63.94 (C-12), 56.44 (C-3), 56.10 (C-10), 25.09 (C-11).

HRMS (m/z): $[M+Na]^+$ calculated for $C_{12}H_{13}N_1O_5Na$ 274.06859, found 274.06836.

N^2-(1-(4-((Propargyl)oxy)-5-methoxy-2-nitrophenyl)ethyl)oxycarbonylguanosine (7)

Guanosine (850 mg; 3.00 mmol, 1.00 eq.) and 4-dimethylaminopyridine (40 mg, 0.33 mmol, 0.11 eq.) were suspended in dry dichloromethane (60 mL) and cooled by an ice-water bath. Dry pyridine (10 mL) and trimethylsilyl chloride (2.4 mL, 18.9 mmol, 6.3 eq) were added and the reaction mixture was stirred for 2 h. The ice-water bath was renewed and a solution of phosgene in toluene (3.0 mL of 15 wt% solution, ~4.2 mmol, 1.4 eq.) was added dropwise. The newly formed yellow precipitate was stirred for 30 min before adding compound **5** (2.44 g, 9.71 mmol, 3.24 eq.) dissolved in dry tetrahydrofuran (20 mL). The reaction mixture was stirred overnight. Afterwards, the reaction mixture was diluted with chloroform (100 mL) and washed with ddH_2O (3x100 mL). The solvent was removed under reduced pressure until an oil was obtained. The oil was dissolved in 1,4 dioxane (50 mL). Subsequently, aqueous ammonia (2.5 mL of 25 % solution) was added and the mixture was vigorously stirred overnight at room temperature. Silica gel (25 mL) was added to the mixture and the

obtained slush was lyophilized to obtain a dry load for purification on PuriFlash XS530 (PF-50SIHP-F0120 column, eluent 95 : 5 to 70 : 30 chloroform : methanol in 7 CV). The solvent was removed under reduced pressure to obtain the product as a yellow-white solid (763 mg, 1.36 mmol, 45 % yield).

^{1}H-NMR (599 MHz, DMSO-*d*$_6$): δ 8.23 (d, *J* = 1.6 Hz, 1H, H-8), 7.77 (s, 1H, H-5″), 7.28 (d, *J* = 4.1 Hz, 1H, H-8″), 6.39 (q, *J* (11″,12″) = 6.3 Hz, 1H, H-12″), 5.79 (dd, *J* = 6.0, 2.6 Hz, 1H, H-1′), 5.45 (t, *J* = 5.3 Hz, 1H, 2′-OH), 5.16 (dd, *J* (3′,3′-OH) = 4.6, *J* = 2.7 Hz, 1H, 3′-OH), 5.00 (q, *J* = 5.4 Hz, 1H, 5′-OH), 4.95 (d, *J* (1″,3″) = 2.3 Hz, 2H, H-3″), 4.48 – 4.41 (m, 1H, H-2′), 4.13 (q, *J* = 4.7 Hz, 1H, H-3′), 3.98 (d, *J* = 1.8 Hz, 3H, H-10″), 3.92 – 3.88 (m, 1H, H-4′), 3.66 (t, *J* (1″,3″) = 2.4 Hz, 1H, H-1″), 3.56 (s, 2H, H-5′), 1.65 (d, *J* (11″,12″) = 6.4 Hz, 3H, H-11″).

^{13}C-NMR (151 MHz, DMSO-*d*$_6$): δ 155.04 (C-9″), 153.94 (C-13″), 149.09 (C-4), 147.25 (C-2), 145.48 (C-4″), 138.80 (C-6″), 137.62 (C-8), 132.27 (C-7″), 119.89 (C-5), 109.71 (C-5″), 109.19 (C-8″), 86.48 (C-1′), 85.42 (C-4′), 79.28 (C-1″), 78.33 (C-2″), 73.98 (C-2′), 70.32 (C-3′), 69.79 (C-12″), 66,35 (C-5′), 61.25, 56.49 (C-3″), 56.40 (C-10″), 21.76 (C-11″).

HRMS (m/z): $[M+Na]^+$ calculated for $C_{23}H_{24}N_6O_{11}Na$ 583.13953, found 583.13936.

N^2-(1-(4-((Propargyl)oxy)-5-methoxy-2-nitrophenyl)ethyl)oxycarbonyl-7-methyl-guanosine (8)

Iodomethane (570 µL, 505 mg, 20 eq.) was added to a solution of compound **7** (256 mg, 0.457 mmol, 1.0 eq.) in dry dimethyl sulfoxide (3 mL) and the reaction mixture was stirred for 5 h at room temperature. After careful quenching with cold triethylammonium bicarbonate buffer (1 M, 3 mL), the crude mixture was directly purified on PrepChrom C-700 V5 (Teledyne C18-aq 150 g column, eluent 100 % triethylammonium bicarbonate buffer (0.1 M) to 60 % acetonitrile in 10 CV). Pure fractions were lyophilized to obtain the product as white fluffy solid (182 mg, 0.259 mmol, 57 % yield as iodide).

^{1}H-NMR (599 MHz, DMSO-*d*$_6$): δ 9.14 (s, 1H, H-8), 7.75 (d, *J* = 1.6 Hz, 1H, H-5″), 7.42 (s, 1H, H-8″), 6.27 (m, 1H, H-12″), 5.95 (s, 1H, 2′-OH), 5.82 (dd, *J* = 10.9, 5.2 Hz, 1H, H-1′), 5.22 (s, 1H, 3′-OH), 4.98 (s, 1H, 5′-OH), 4.94 (d, *J* = 2.4 Hz, 2H, H-3″), 4.53 – 4.47 (m, 1H, H-2′), 4.16 (d, *J* = 5.9 Hz, 1H, H-3′), 4.05 (s, 3H, N7-CH_3), 4.04 (s, 1H, H-4′), 3.98 (s, 3H, H-10″), 3.65 (t, *J* = 2.3 Hz, 1H, H-1″), 3.64 – 3.57 (m, 1H, H-5′), 3.56 – 3.51 (m, 1H, H-5′), 1.55 (d, *J* = 6.3 Hz, 3H, H-11″).

^{13}C-NMR (151 MHz, DMSO-*d*$_6$): δ 154.09 (C-9″), 145.19 (C-4″), 138.57 (C-6″), 134.08 (C-7″), 109.69 (C-5″), 109.36 (C-8″), 90.01 (C-1′), 87.29 (C-4′), 79.23 (C-1″), 78.39 (C-2″), 74.69 (C-2′), 70.78 (C-3′), 67.46 (C-12″), 61.07 (C-5′), 56.48 (C-10″), 35.15 (N7-CH_3), 22.15 (C-11″).

HRMS (m/z): M^+ calculated for $C_{24}H_{26}N_6O_{11}$ 575.17323, found 575.17429.

N^2-(1-(4-((Propargyl)oxy)-5-methoxy-2-nitrophenyl)ethyl)oxycarbonyl-7-methyl-guanosine 5′-phosphate (9)

The iodide salt of compound **8** (134 mg, 0.191 mmol, 1.0 eq.) was dissolved in dry trimethyl phosphate (2 mL). Freshly distilled $POCl_3$ (100 µL, 164 mg, 1.07 mmol, 5.6 eq.) was added and the reaction mixture was stirred for 1.5 h in an ice-water bath. Subsequently, the reaction mixture was quenched with a cold solution of triethylammonium bicarbonate (1M, 1.5 mL) and directly purified via liquid injection on PrepChrom C-700 V5 (Teledyne C18-aq 100 g column, eluent 100 % triethylammonium bicarbonate buffer (0.1 M) to 60 % acetonitrile in 10 CV). Pure fractions were lyophilized to obtain the product as white fluffy solid (86 mg, 0.114 mmol, 60 % yield as triethylammonium salt).

^{1}H NMR (599 MHz, D_2O): δ 7.55 (d, *J* = 9.7 Hz, 1H, H-5″), 7.18 (s, 1H, H-8″), 6.27 (dd, *J* = 12.6, 6.3 Hz, 1H, H-12″), 6.06 (d, *J* = 3.8 Hz, 1H, H-1′), 4.70 (s, 2H, H-3″), 4.60 – 4.55 (m, 1H, H-2′), 4.45 (dt, *J* = 9.8, 4.9 Hz, 1H, H-3′), 4.36 (s, 1H, H-4′), 4.16 (s, 4H, N7-CH_3), 4.05 (s, 1H, H-5′), 3.90 (s, 2H, H-10″), 2.96 (d, *J* = 2.6 Hz, 1H, H-1″), 1.57 (dd, *J* = 6.5, 2.5 Hz, 3H, H-11″).

^{13}C NMR (151 MHz, D_2O): δ 153.87 (C-9″), 153.09, 147.89, 145.02 (C-4″), 138.70 (C-6″), 136.68, 133.32 (C-7″), 111.38, 109.63 (C-5″), 108.84 (C-8″), 89.69 (C-1′), 84.69 (C-4′), 77.28 (C-1″), 75.34 (C-2′), 70.33 (C-12″), 69.42 (C-3′), 62.81 (C-5′), 56.81 (C-3″), 56.30 (C-10″), 35.90 (C-7), 20.76 (C-11″).

^{31}P NMR (243 MHz, D_2O): δ 2.17.

HRMS (m/z): M^+ calculated for $C_{24}H_{28}N_6O_{14}P$ 655.13959, found 655.14001.

Guanosine 5′-diphosphate imidazolide (10)

Compound **10** was synthesized according to a method of Jemielity et al.[43] Tris(triethylammonium) salt of guanosine 5′-diphosphate (1.863 g, 2.49 mmol, 1.0 eq.), imidazole (848 mg, 12.5 mmol, 5.0 eq.), triphenylphosphine (1.306 g, 4.98 mmol, 2.0 eq.) and aldrithiol-2 (1.097 g, 4.98 mmol, 2.0 eq.) were disolved in dry dimethylformamide (23 mL). Subsequently, triethylamine (345 µL, 2.49 mmol, 1.0 eq.) was added and the reaction mixture was stirred overnight. The next day, the reaction mixture was mixed with anhydrous sodium perchlorate (1.8 g) dissolved in dry acetone (90 mL) to obtain the product as precipitate. The precipitate was washed thrice with dry cold acetone (15 mL) and lyophilized to obtain the product as yellow solid (quantitative yield).

CyCliCap (1)

The triethylammonium salt of compound **9** (71.5 mg, 0.095 mmol, 1.0 eq), the sodium salt of GDP-imidazolide **10** (139 mg, 0.259 mmol, 2.7 eq.) and dry $ZnCl_2$ (365 mg, 2.67 mmol, 28 eq.) were suspended in dry dimethylformamide (2.5 mL). The reaction

mixture was stirred for 66 h at room temperature. After quenching with cold triethylammonium bicoarbonate buffer (2 M, 1 mL) and EDTA (0.5 M pH 7.94, 1 mL), the crude mixture was directly purified on PrepChrom C-700 V5 (Teledyne C18-aq 100 g column, eluent 100 % triethylammonium bicarbonate buffer (0.1 M) to 60 % acetonitrile in 10 CV). The appropriate fractions were lyophilized, dissolved in ddH$_2$O (1 mL) and purified via ion exchange chromatography (POROS-50HQ column, eluent 100 % ddH$_2$O to 100 % triethylammonium bicarbonate (1 M), step-wise gradient). Pure fractions were combined and lyophilized to obtain the triethylammonium salt of the product, which was converted to the sodium salt by sodium loaded Dowex 50WX8 resin. The fractions containing product were lyophilized and the product was obtained as fluffy solid (17.5 mg, 0.017 mmol, 16% yield).

^{1}H NMR (599 MHz, D$_2$O): δ 7.98 (s, 1H, H-B8′), 7.72 (d, *J* = 20.6 Hz, 1H, H-5″), 7.27 (d, *J* = 17.4 Hz, 1H, H-8″), 6.33 (dq, *J* = 12.8, 6.1 Hz, 1H, H-12″), 6.02 (dd, *J* = 8.1, 3.3 Hz, 1H, H-A1′), 5.78 (d, *J* = 6.0 Hz, 1H, H-B1′), 4.84 (d, *J* = 2.3 Hz, 1H), 4.82 (d, *J* = 2.4 Hz, 2H, H-3″), 4.66 – 4.62 (m, 1H, H-B2′), 4.57 – 4.52 (m, 1H, H-A2′), 4.48 – 4.45 (m, 1H, H-B3′), 4.43 (dt, *J* = 10.6, 5.2 Hz, 1H, H-A3′), 4.38 (dd, *J* = 11.3, 3.3 Hz, 1H, H-A4′), 4.32 (t, *J* = 3.0 Hz, 1H, H-B4′), 4.29 – 4.22 (m, 1H, H-A5′/B5′), 4.11 (s, 3H, N7-CH$_3$), 3.94 (d, *J* = 13.2 Hz, 3H, H-10″, 3.01 – 2.99 (m, 1H, H-1″), 1.64 (d, *J* = 6.5 Hz, 2H, H-11″).

^{13}C NMR (151 MHz, D$_2$O): δ 158.64, 154.19, 153.94, 153.34 (C-9″), 151.48, 148.19, 145.06 (C-4″), 139.02 (C-6″), 137.14 (C-B8), 134.15 (C-7″), 115.88 (C-B5), 111.66 (C-A5), 110.19 (C-5″), 109.03 (C-8″), 89.95 (C-A1′), 86.67 (C-B1′), 83.97 (C-A4′), 83.73 (C-B4′), 77.58, 75.23 (C-A2′), 73.90 (C-B2′), 70.54 (C-B3′), 70.29, 69.21 (C-A3′), 65.54 (C-B5′), 64.41 (C-A5′), 57.07 (C-3″), 56.72, 56.58 (C-10″), 36.08 (C-A7), 20.91 (C-11″).

^{31}P NMR (243 MHz, D$_2$O): δ -11.43 (td, *J* = 19.8, 4.4 Hz), -22.89 (t, *J* = 18.9 Hz).

HRMS (m/z): M$^+$ calculated for $C_{34}H_{41}N_{11}O_{24}P_3$ 1080.15333, found 1080.15517.

5. Compound Characterization

NMR measurements were performed on the Bruker NEO 400 or Bruker Avance II 400. ^{1}H NMR chemical shifts, given relative to TMS, are referenced to the solvent signal. Spectra of ^{13}C and ^{31}P nuclides are referenced according to the proton resonance of TMS as the primary reference for the unified chemical shift scale.

Exact masses were measured on the Orbitrap Velos Pro, LTQ Orbitrap XL or Exploris 120 Electrospray Orbitrap (all by Thermo Fisher Scientific).

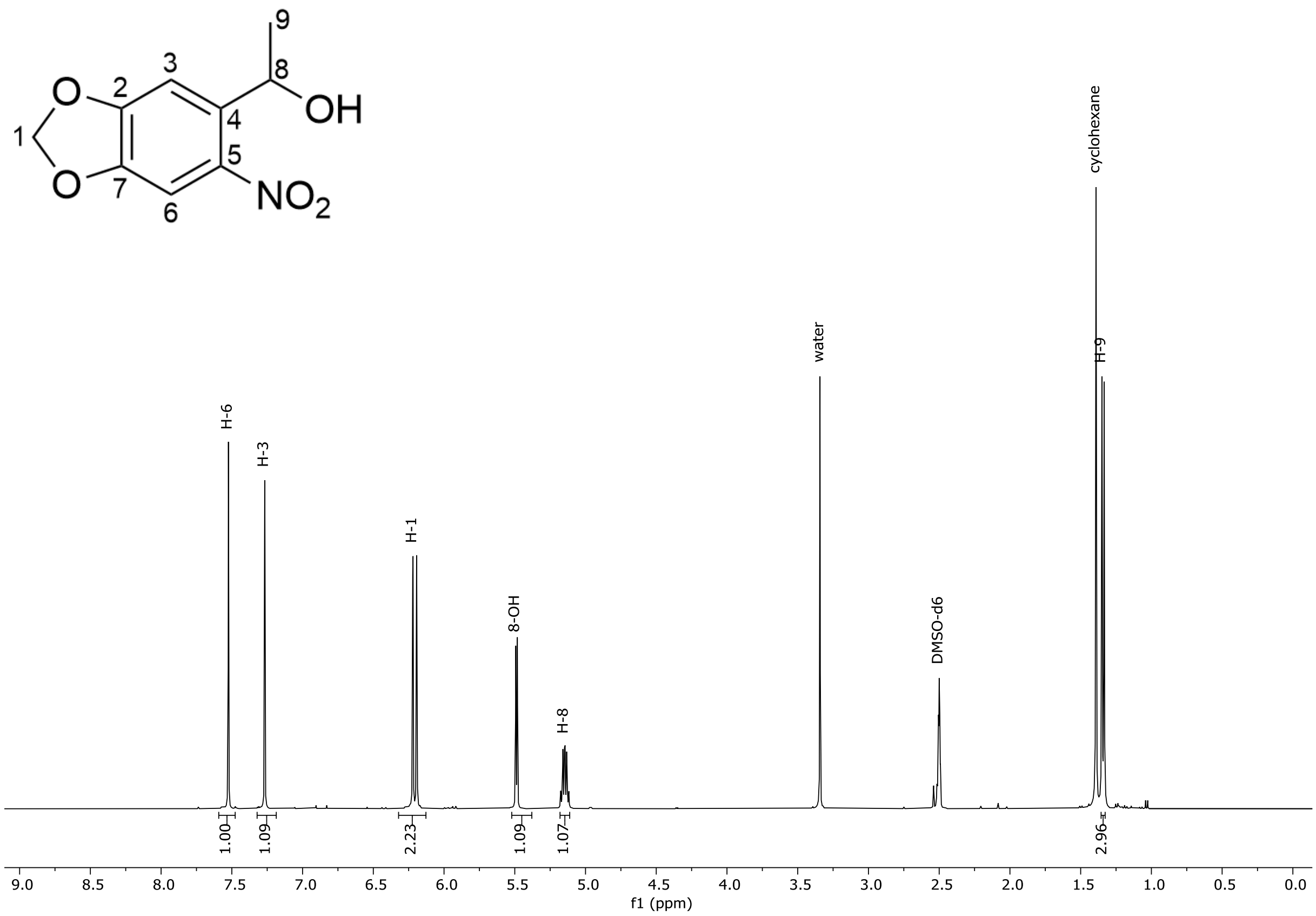

Figure S18 ^{1}H-NMR spectrum of compound **3**.

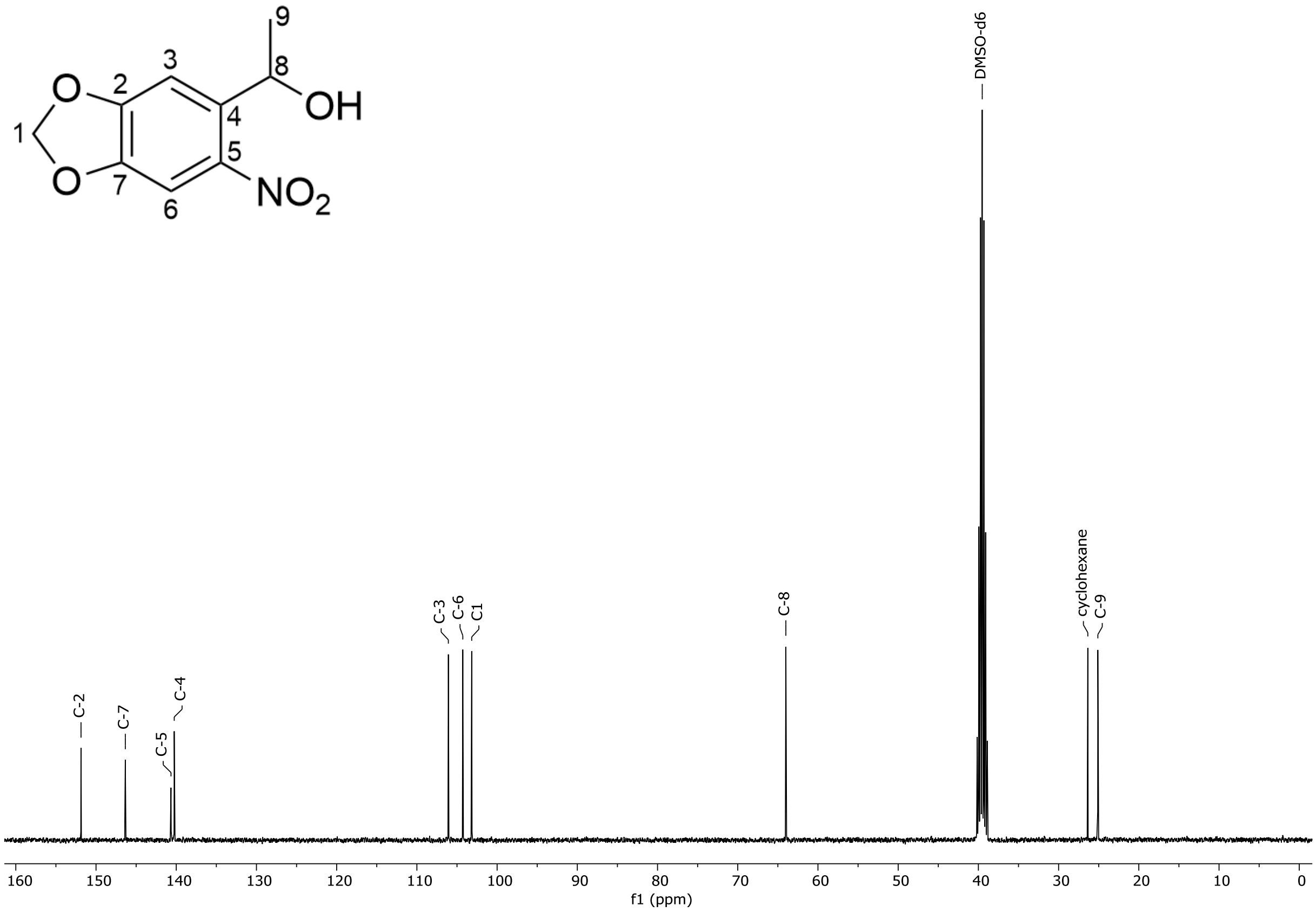

Figure S19 ^{13}C-NMR spectrum of compound **3.**

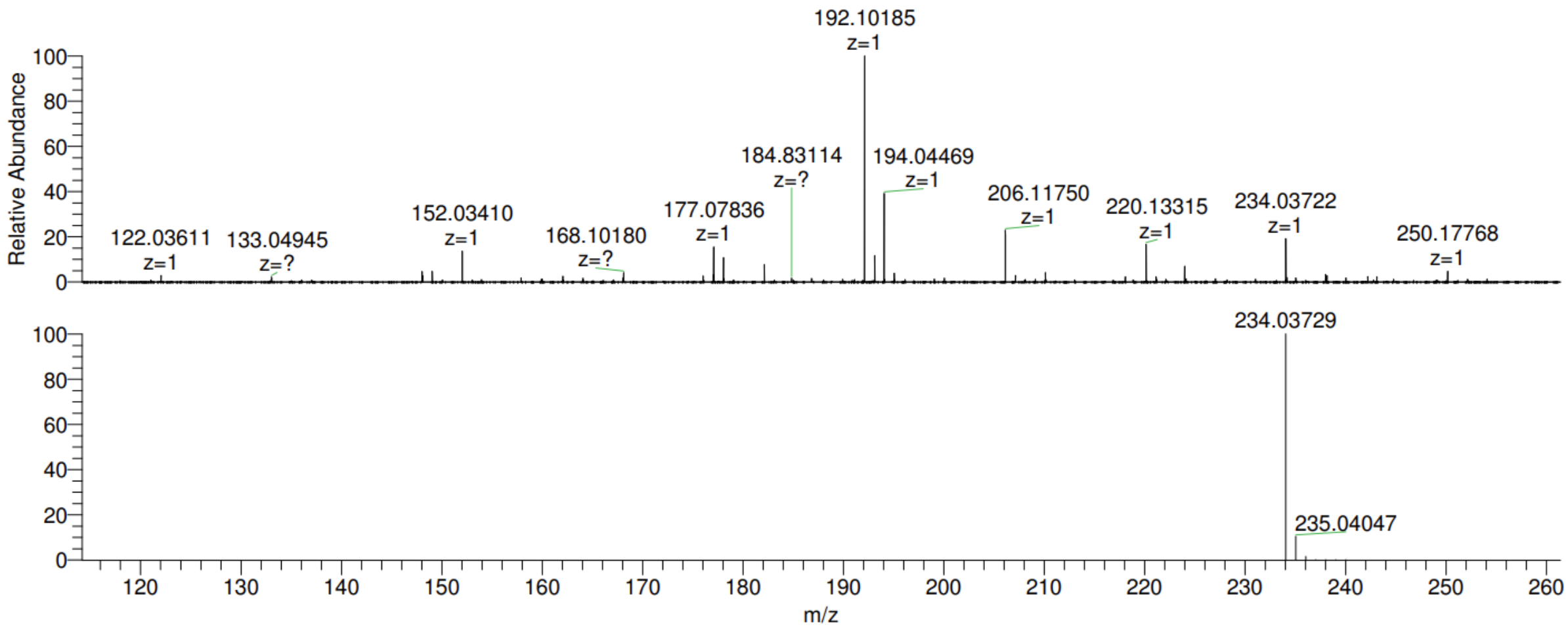

Figure S20 HRMS (m/z) of compound **3**: $[M+Na]^+$ calculated for $C_9H_9N_1O_5Na$ 234.03729, found 234.03722.

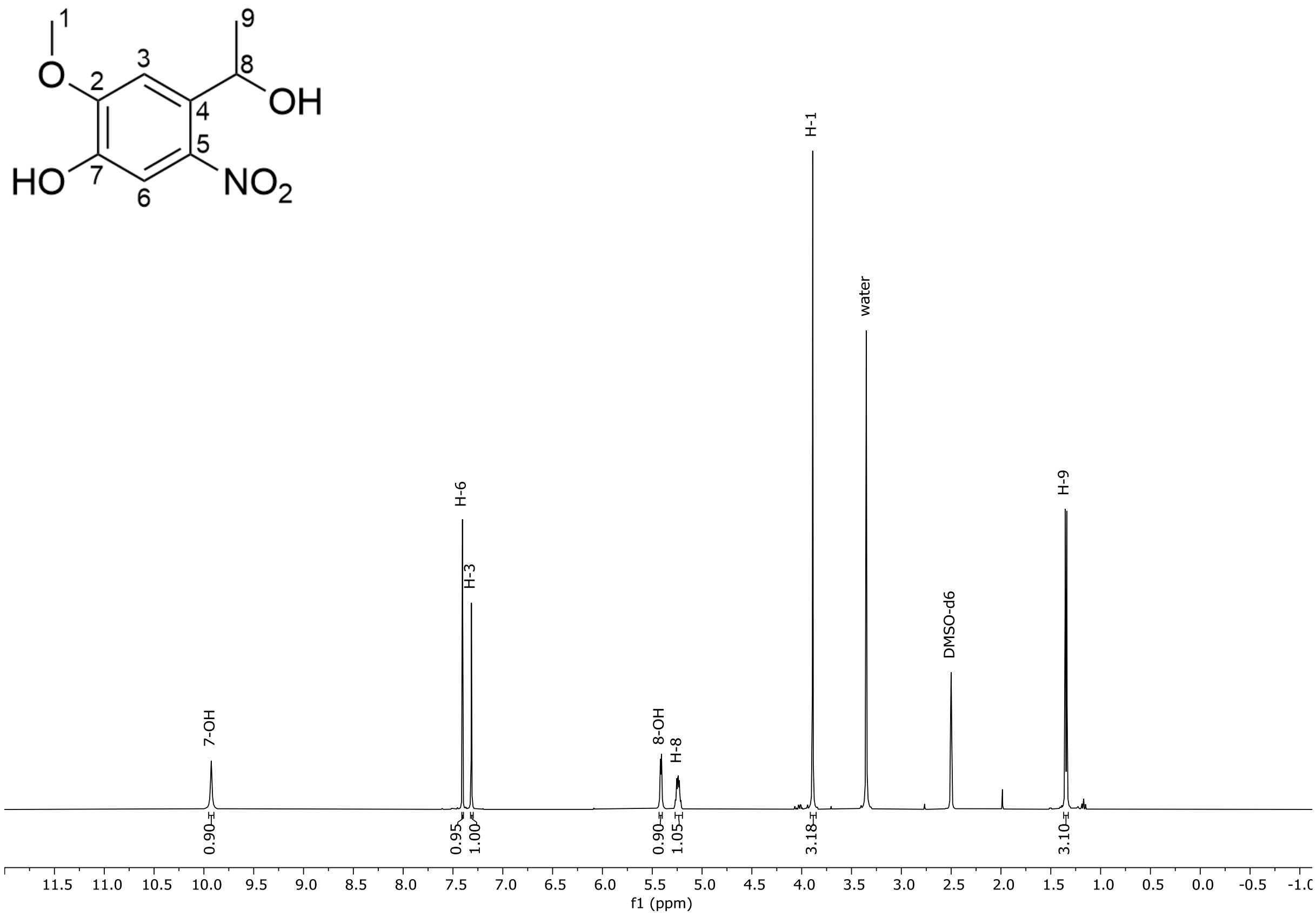

Figure S21 ^{1}H-NMR spectrum of compound **4**.

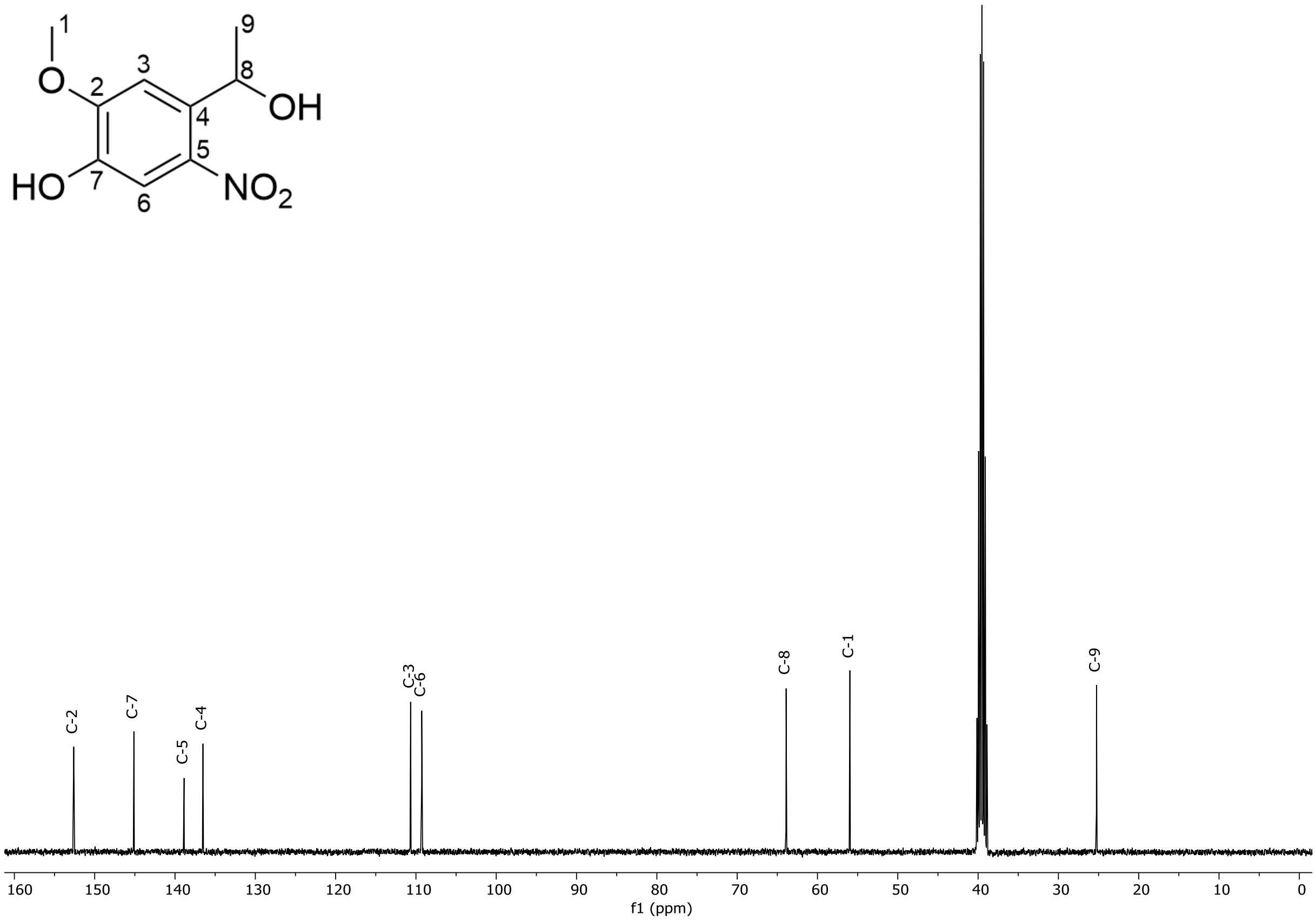

Figure S22 ^{13}C-NMR spectrum of compound **4**.

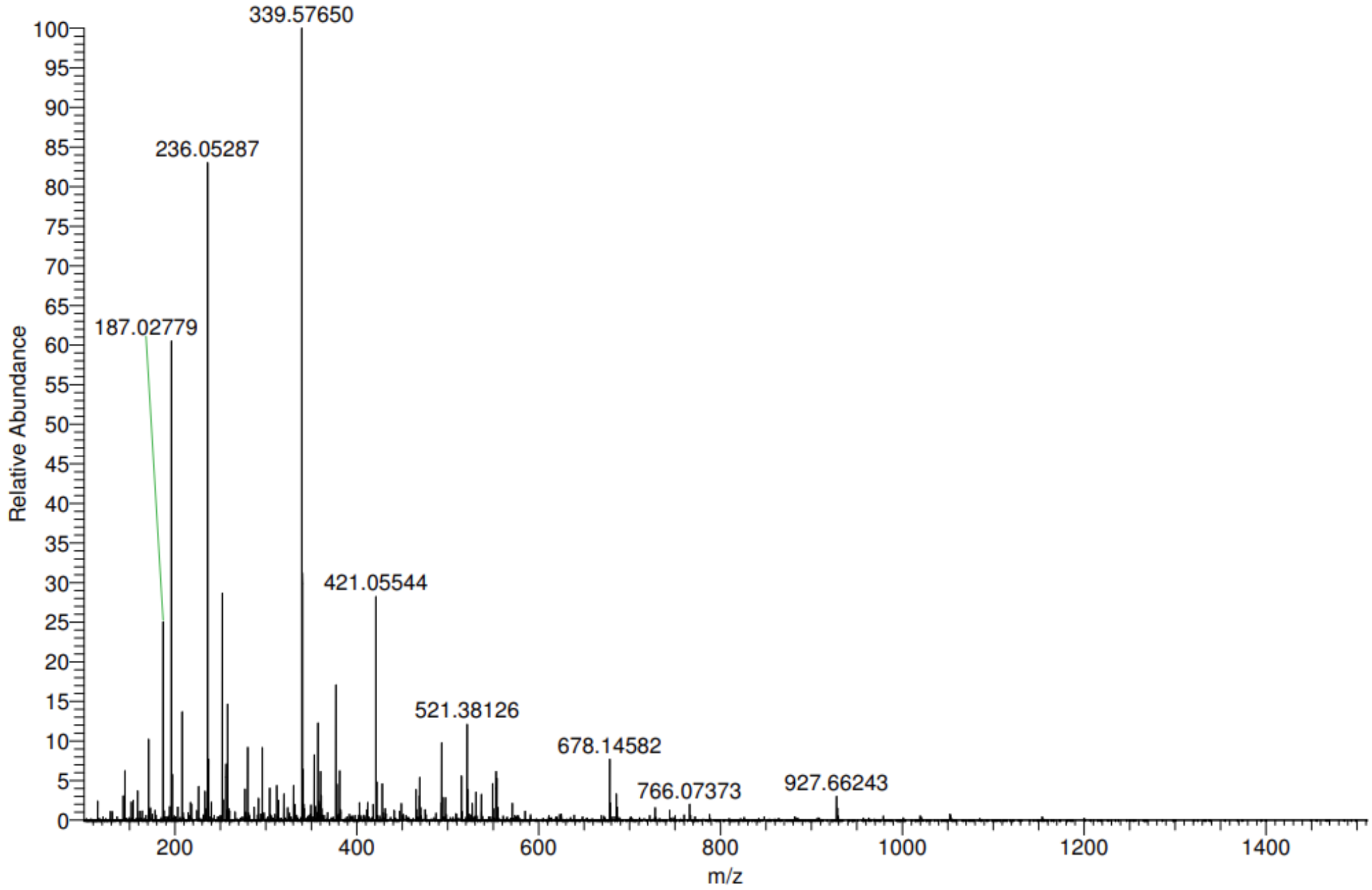

Figure S23 HRMS (m/z) of compound **4**: $[M+Na]^+$ calculated for $C_9H_{11}N_1O_5Na$ 236.05294, found 236.05287.

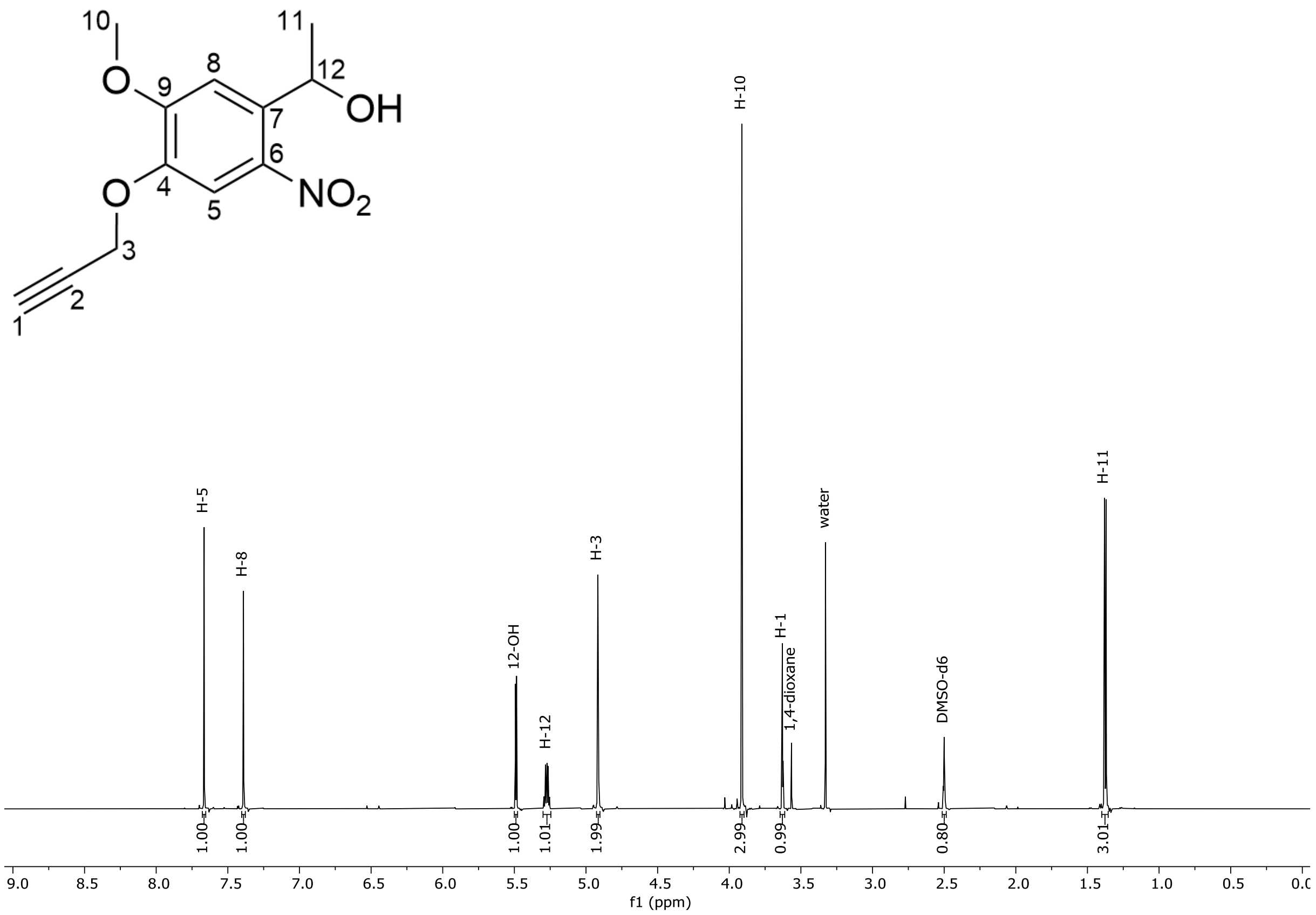

Figure S24 ^{1}H-NMR spectrum of compound **5**.

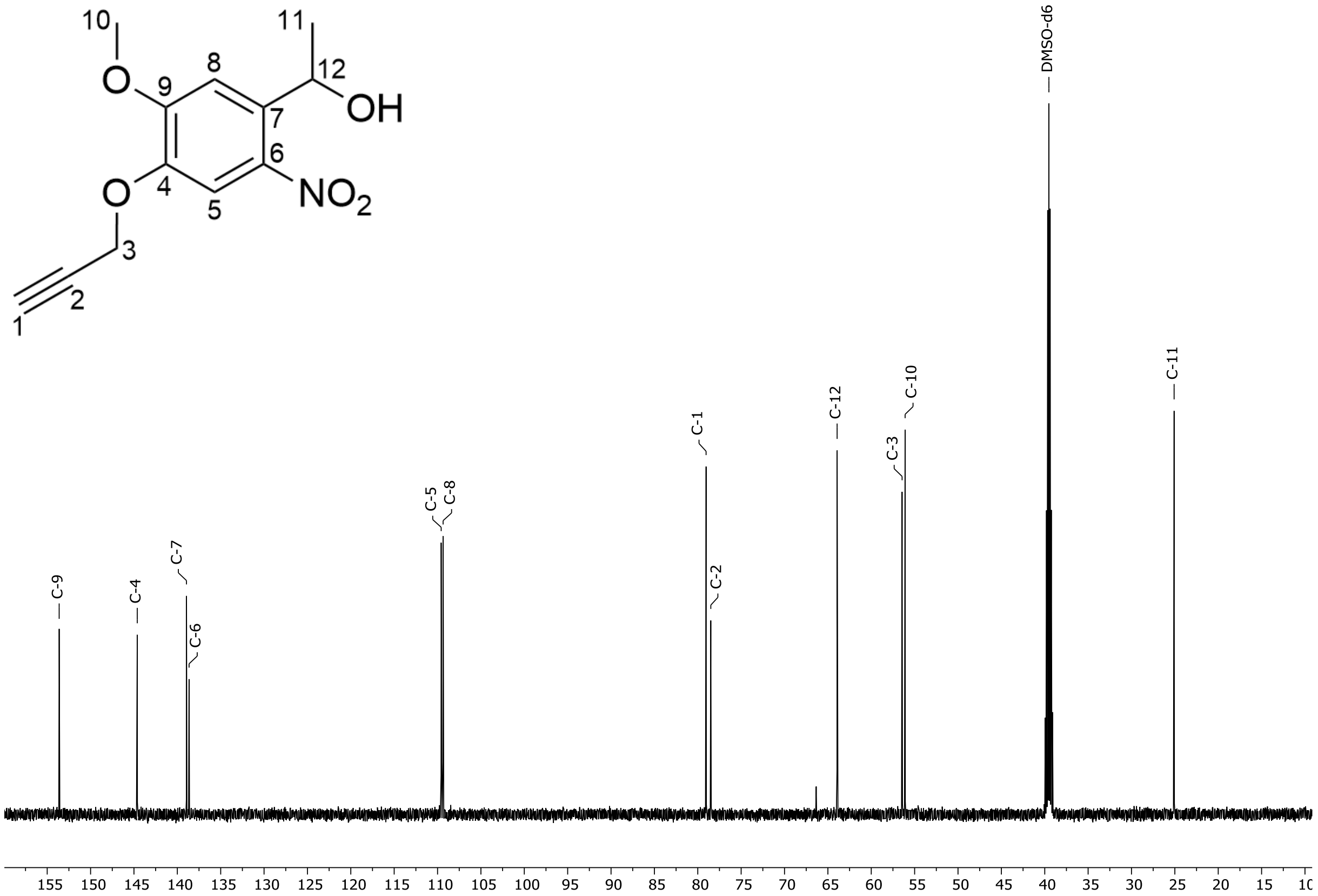

Figure S25 ^{13}C-NMR spectrum of compound **5**.

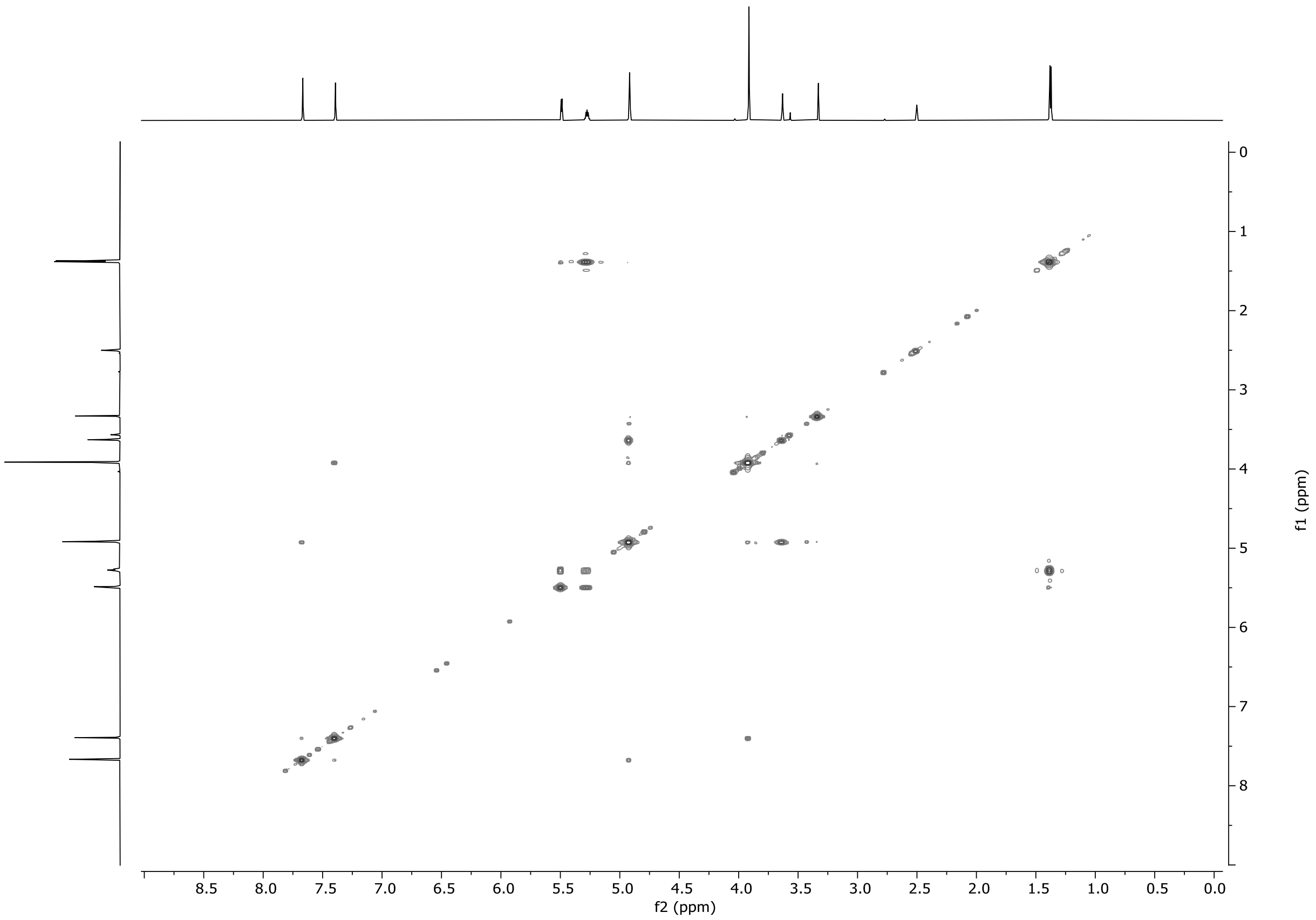

Figure S26 ^1H-^1H COSY spectrum of compound **5**.

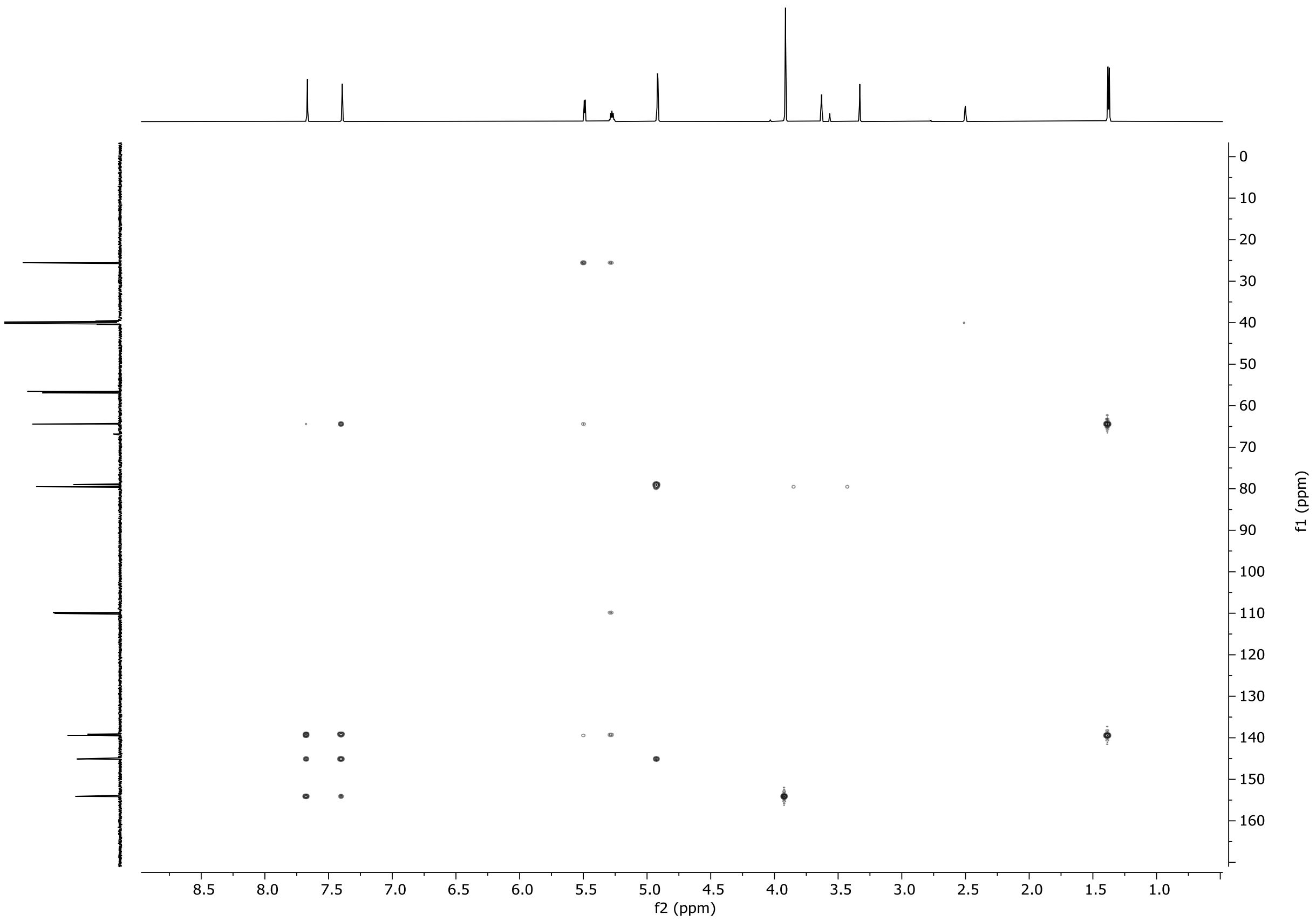

Figure S27 ^1H-^{13}C HMBC spectrum of compound **5**.

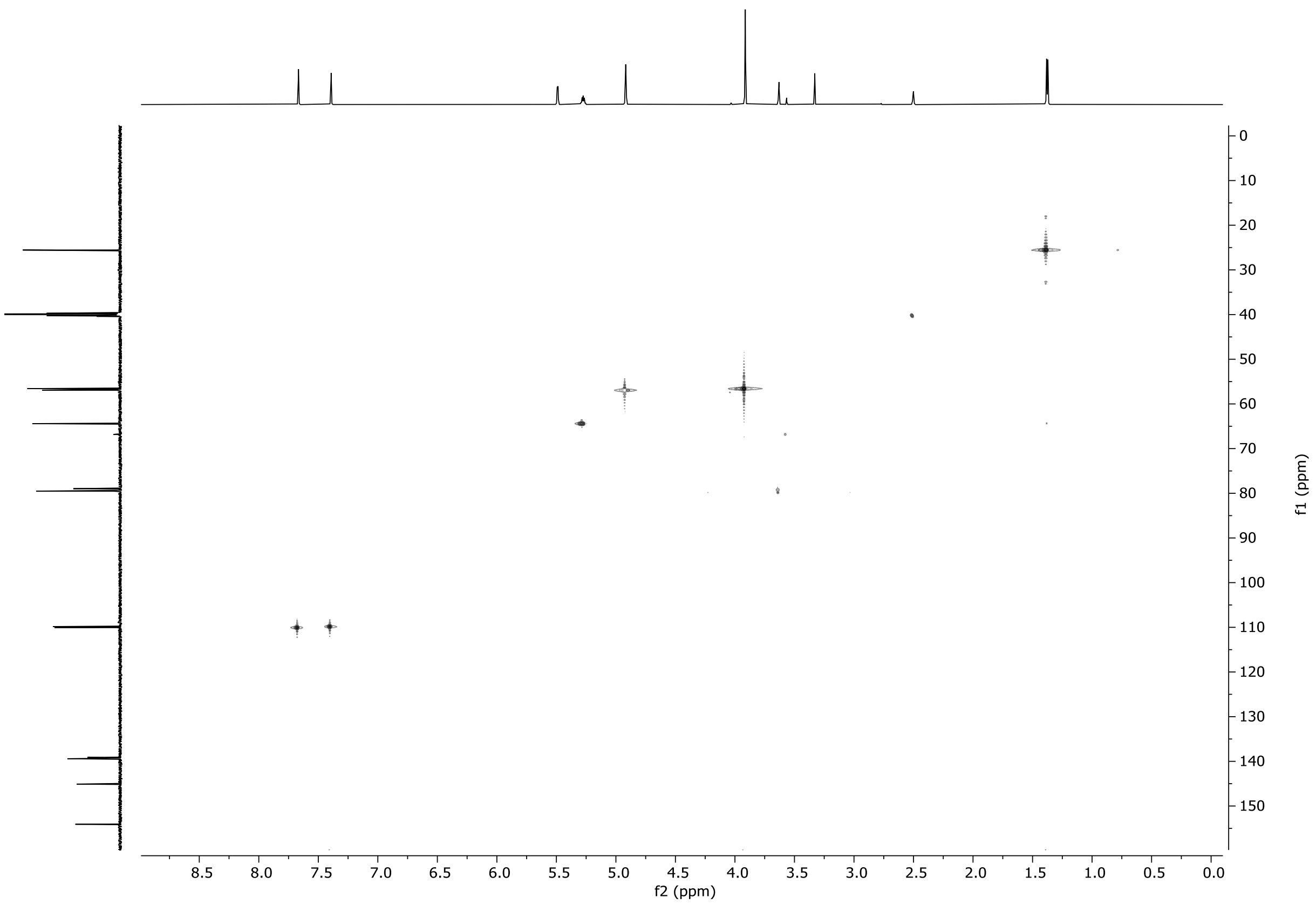

Figure S28 ^{1}H-^{13}C HSQC spectrum of compound **5**.

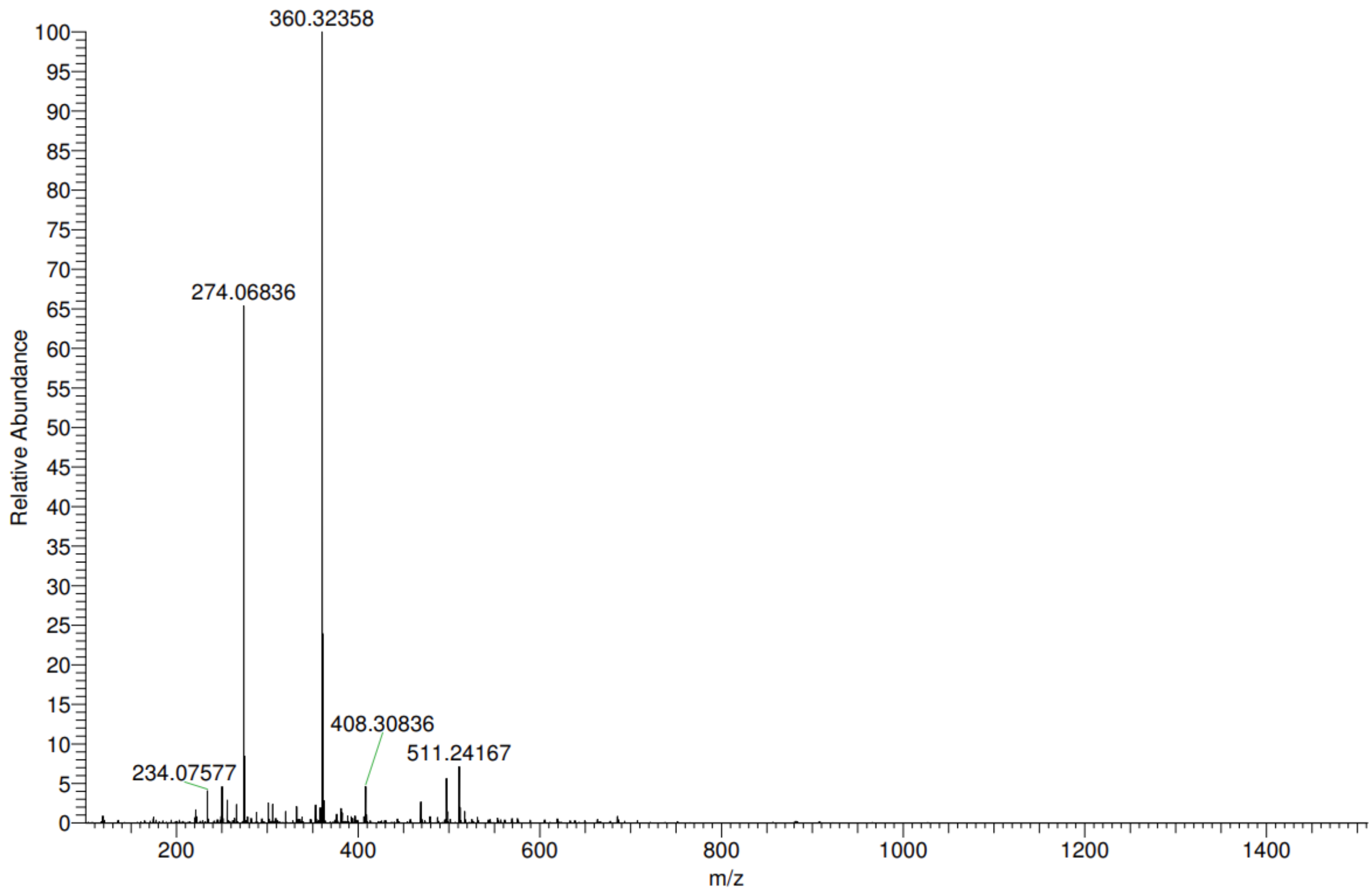

Figure S29 HRMS (m/z) of compound **5**: $[M+Na]^+$ calculated for $C_{12}H_{13}N_1O_5Na$ 274.06859, found 274.06836.

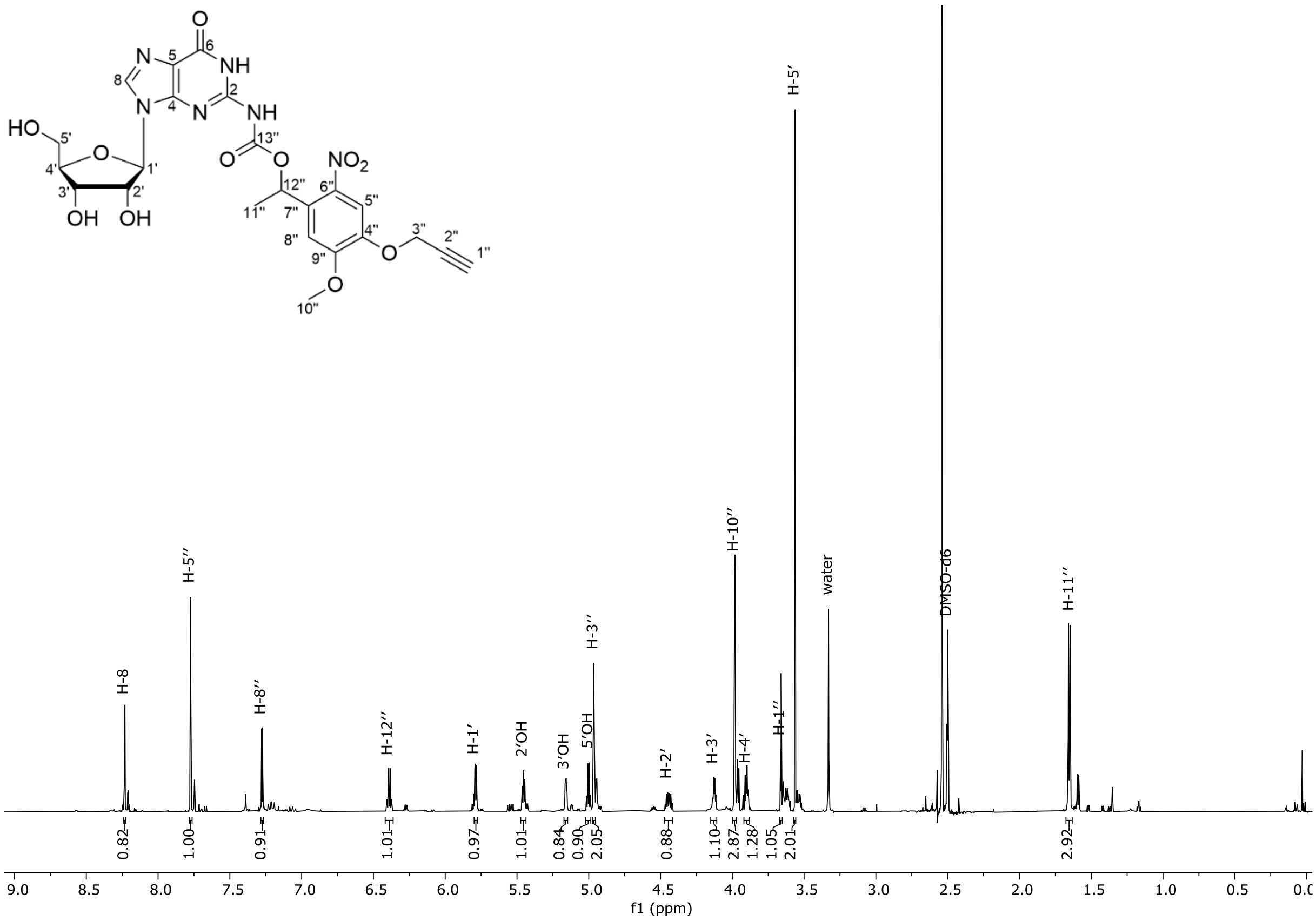

Figure S30 ^{1}H-NMR spectrum of compound **7**.

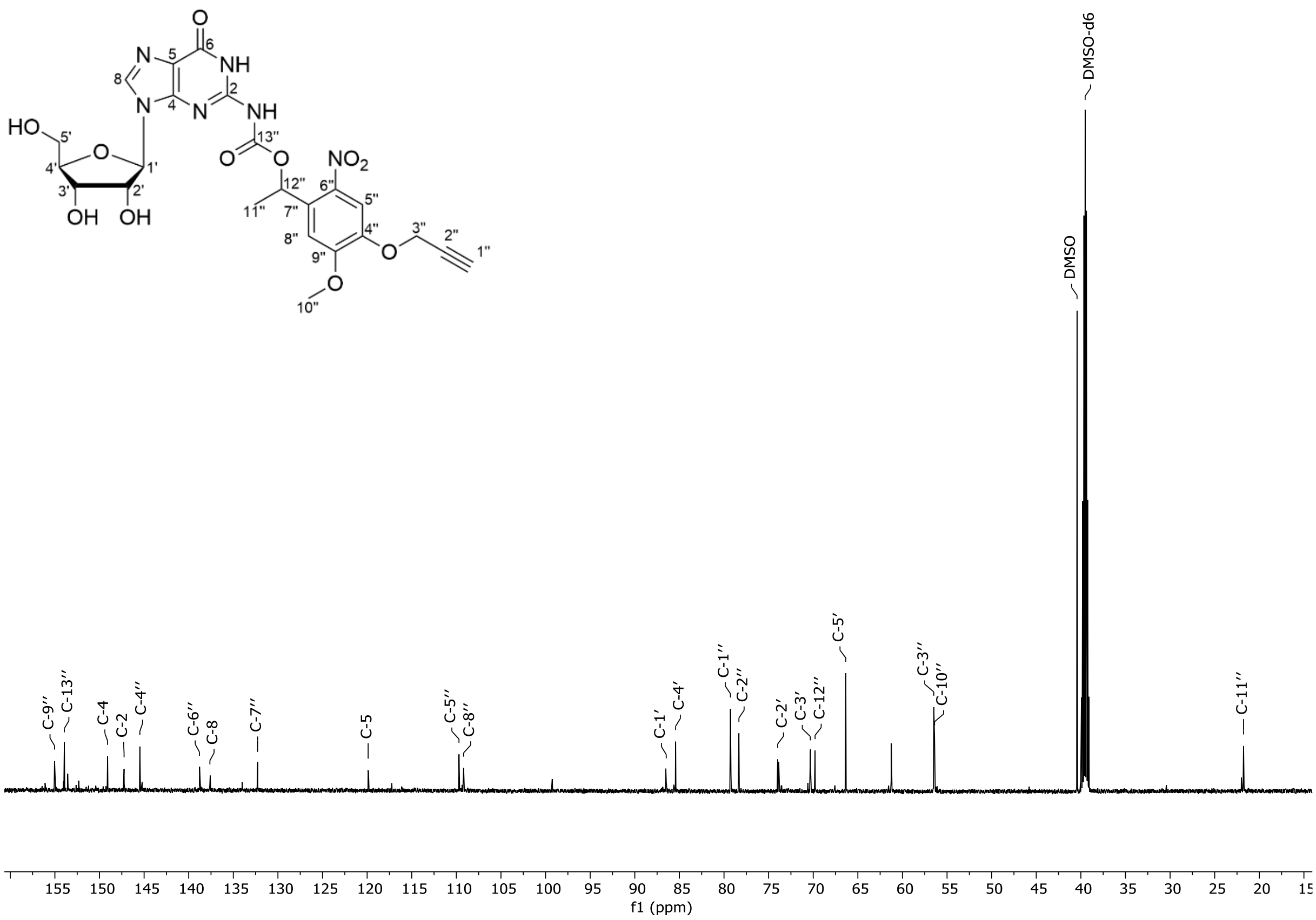

Figure S31 ^{13}C-NMR spectrum of compound **7**.

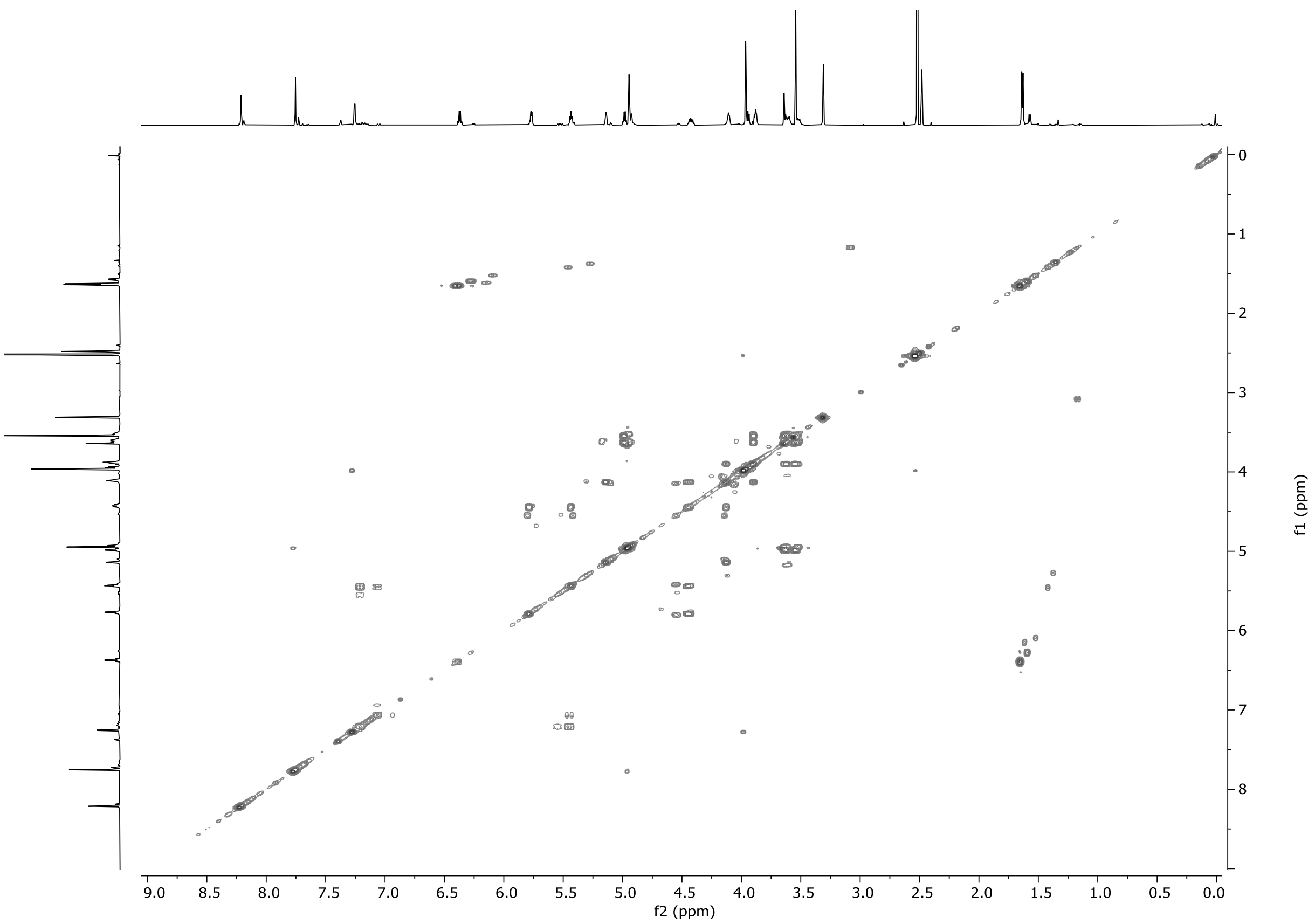

Figure S32 ^{1}H-^{1}H COSY spectrum of compound **7**.

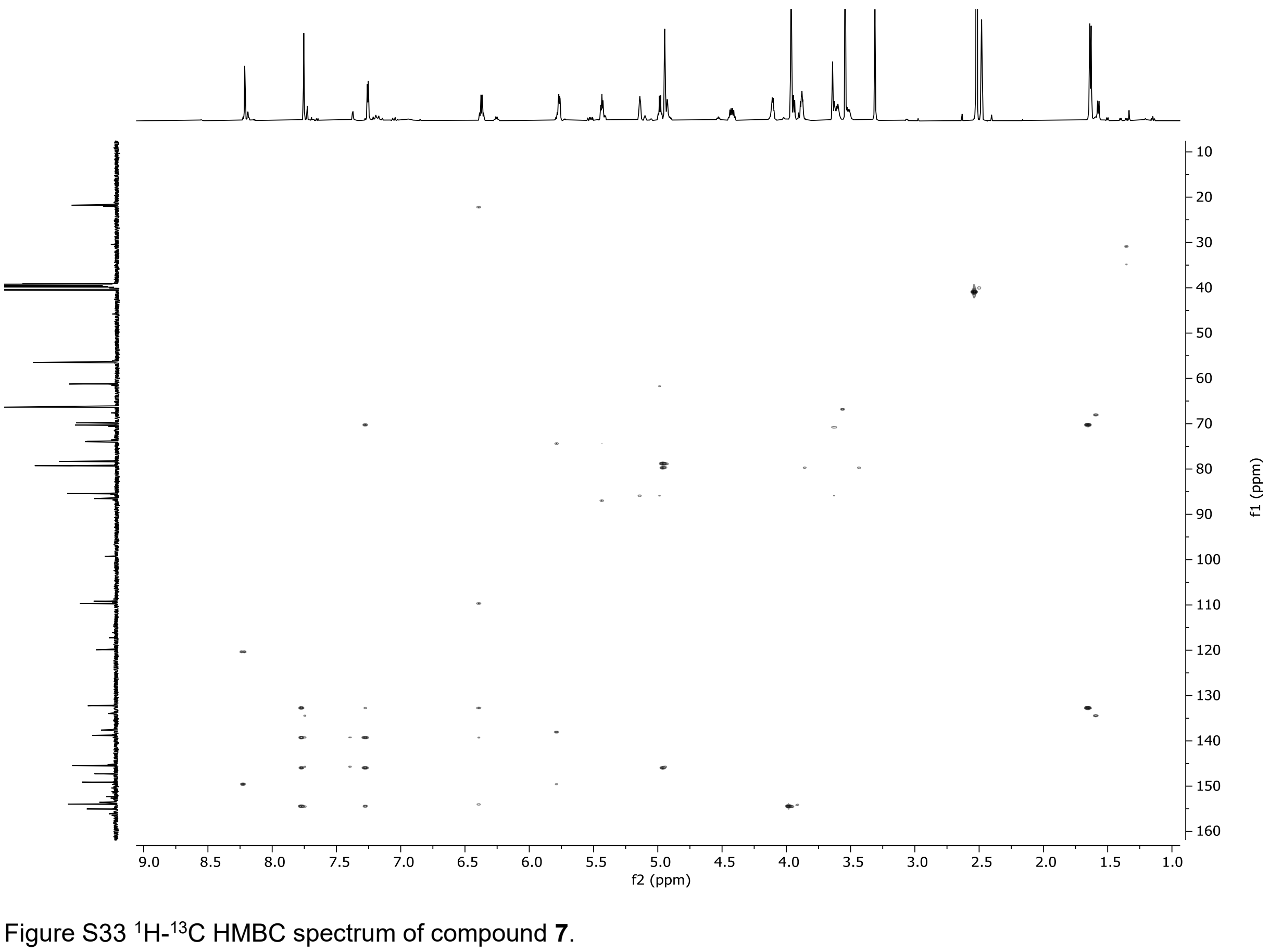

Figure S33 ^{1}H-^{13}C HMBC spectrum of compound **7**.

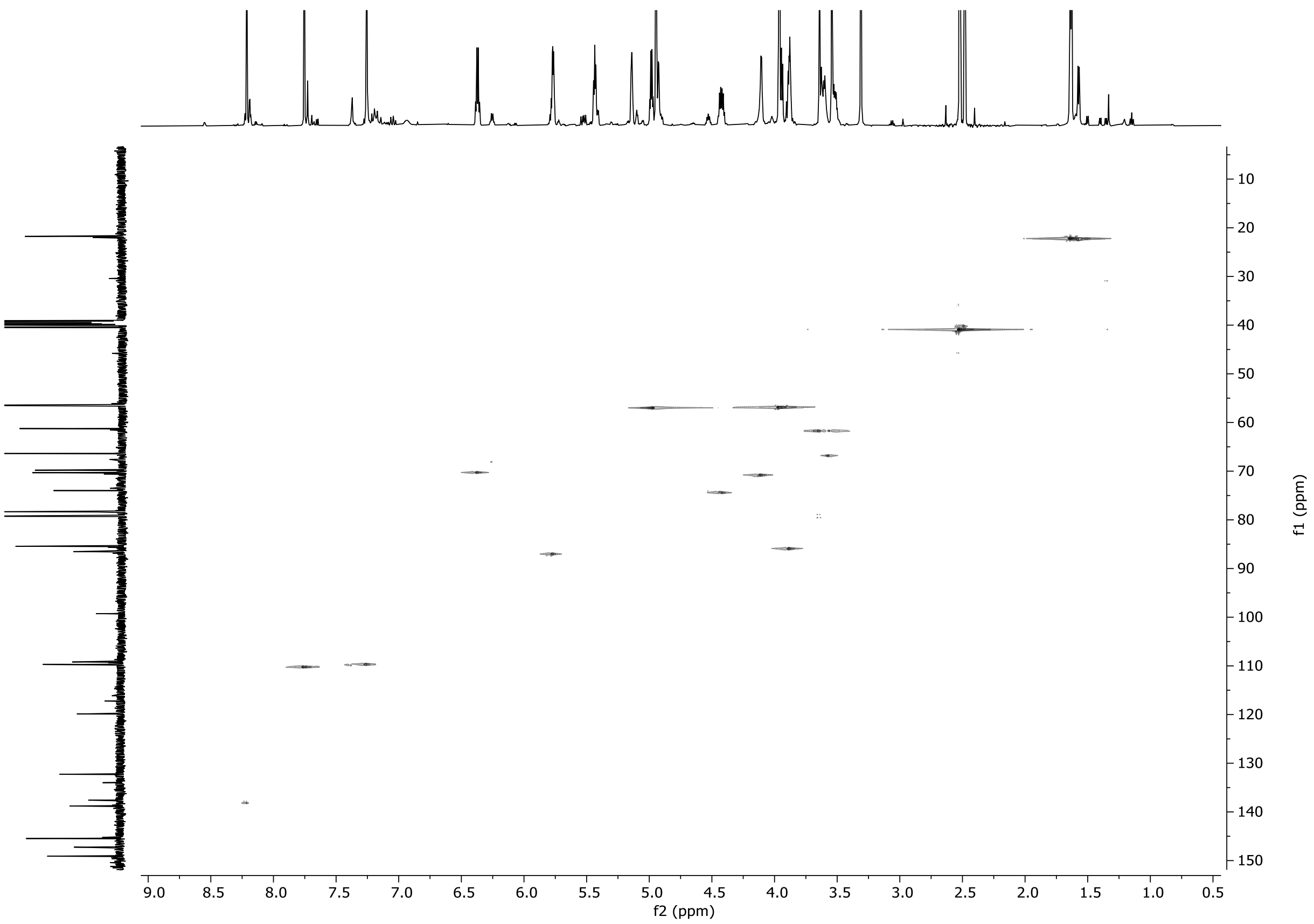

Figure S34 ^{1}H-^{13}C HSQC spectrum of compound **7**.

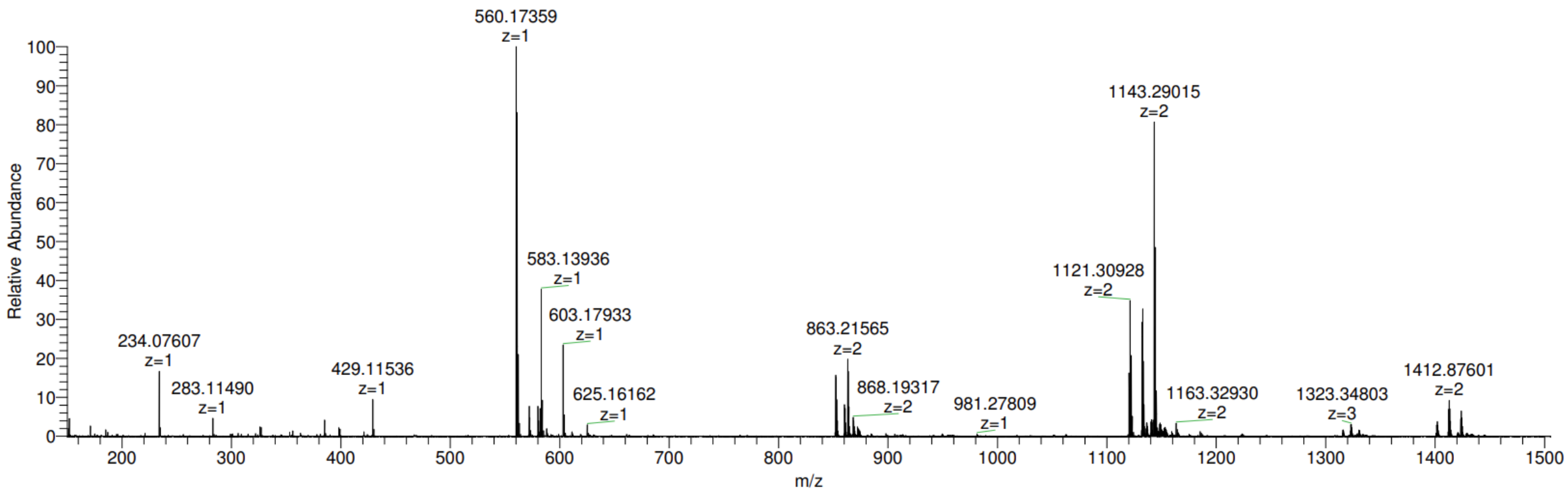

Figure S35 HRMS (m/z) of compound **7**: $[M+Na]^+$ calculated for $C_{23}H_{24}N_6O_{11}Na$ 583.13953, found 583.13936.

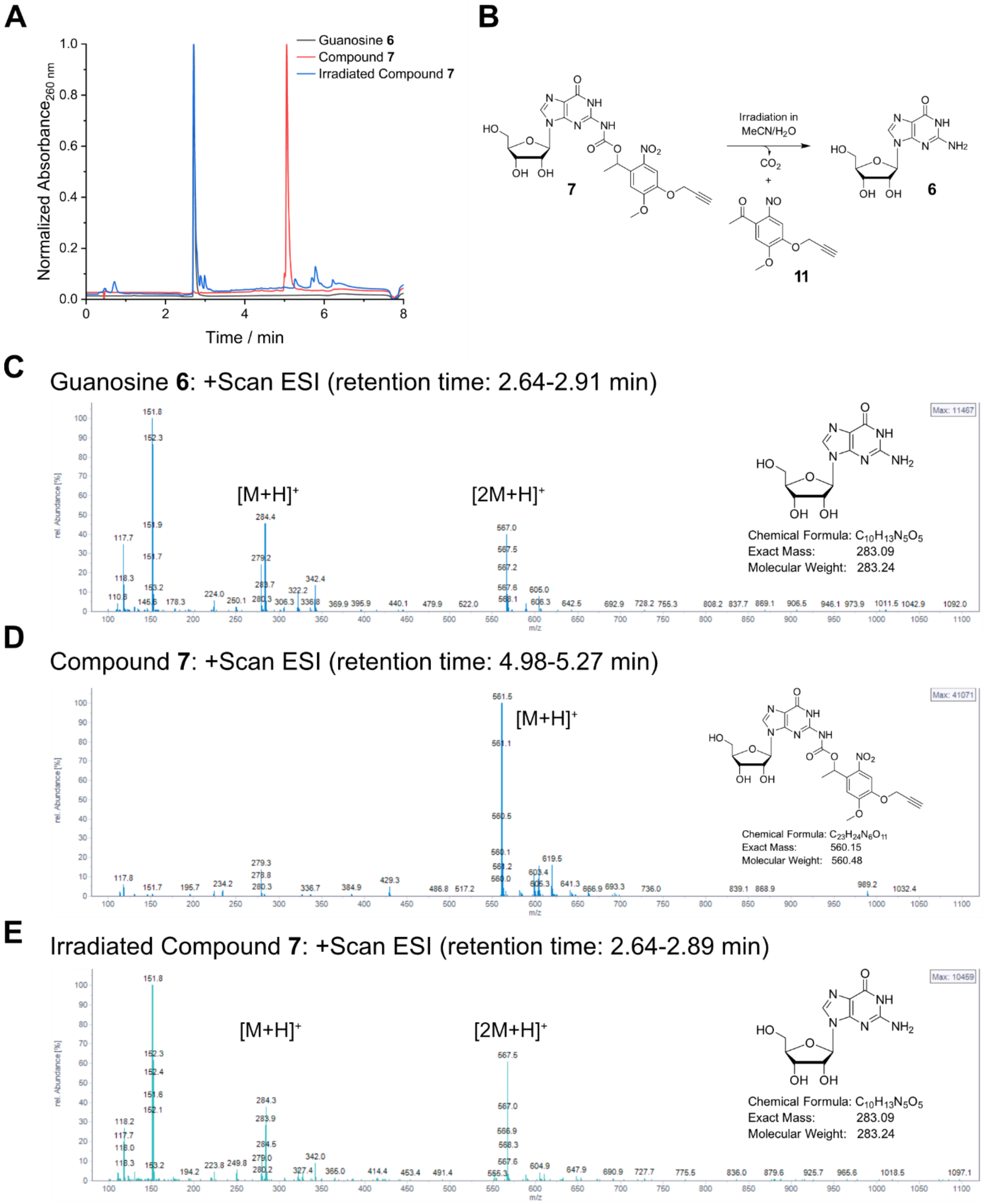

Figure S36 LC-MS analysis of the irradiation of compound 7 (A) HPLC chromatograms of unirradiated and irradiated compound **7** in acetonitrile/water (365 nm, 30 s, 460 mW/cm^2). Guanosine is shown as a reference. (B) Proposed Norrish type 2 photocleavage reaction. (C) Positive-mode ESI mass spectrum of the guanosine **6** reference peak shown in A. (D) Positive-mode ESI mass spectrum of the peak corresponding to compound **7** shown in A. (E) Positive-mode ESI mass spectra of irradiated compound **7** shown in A.

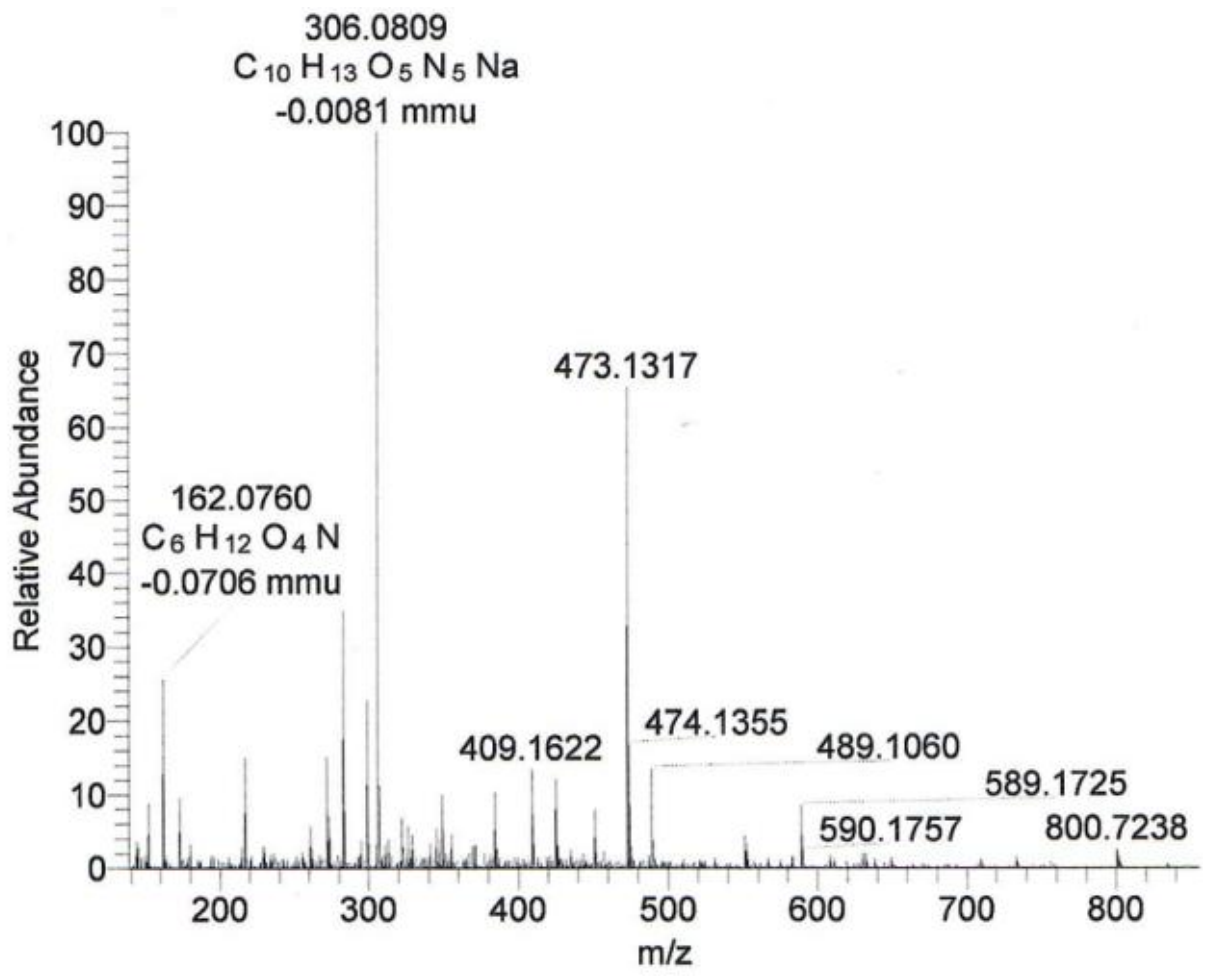

Figure S37 HRMS (m/z) of irradiated compound **7**. Uncaged Guanosine **6** was found: [M+Na]$^+$ calculated for $C_{10}H_{13}N_5O_5Na$ 306.0809, found 306.0809.

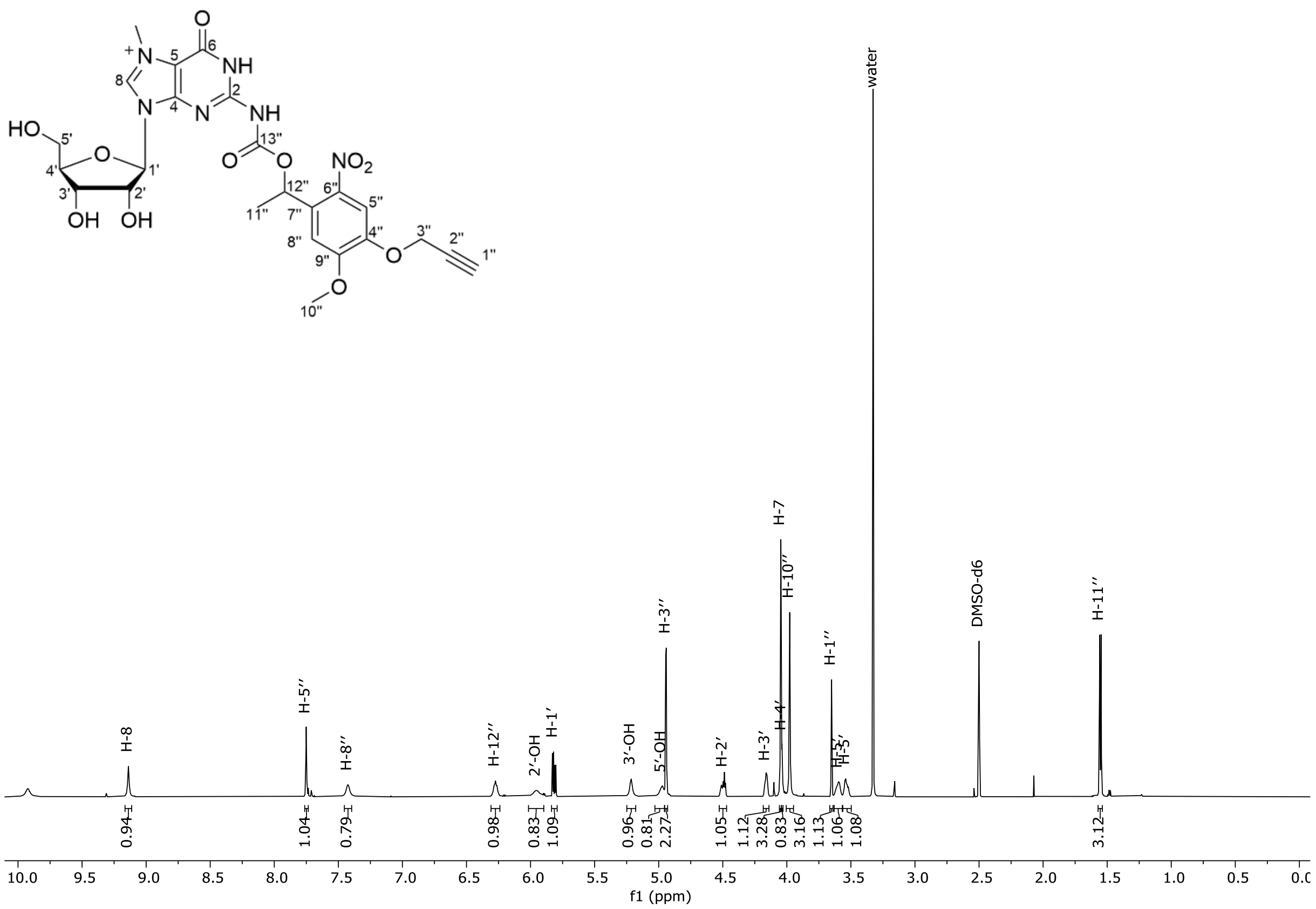

Figure S38 ^{1}H-NMR spectrum of compound **8**.

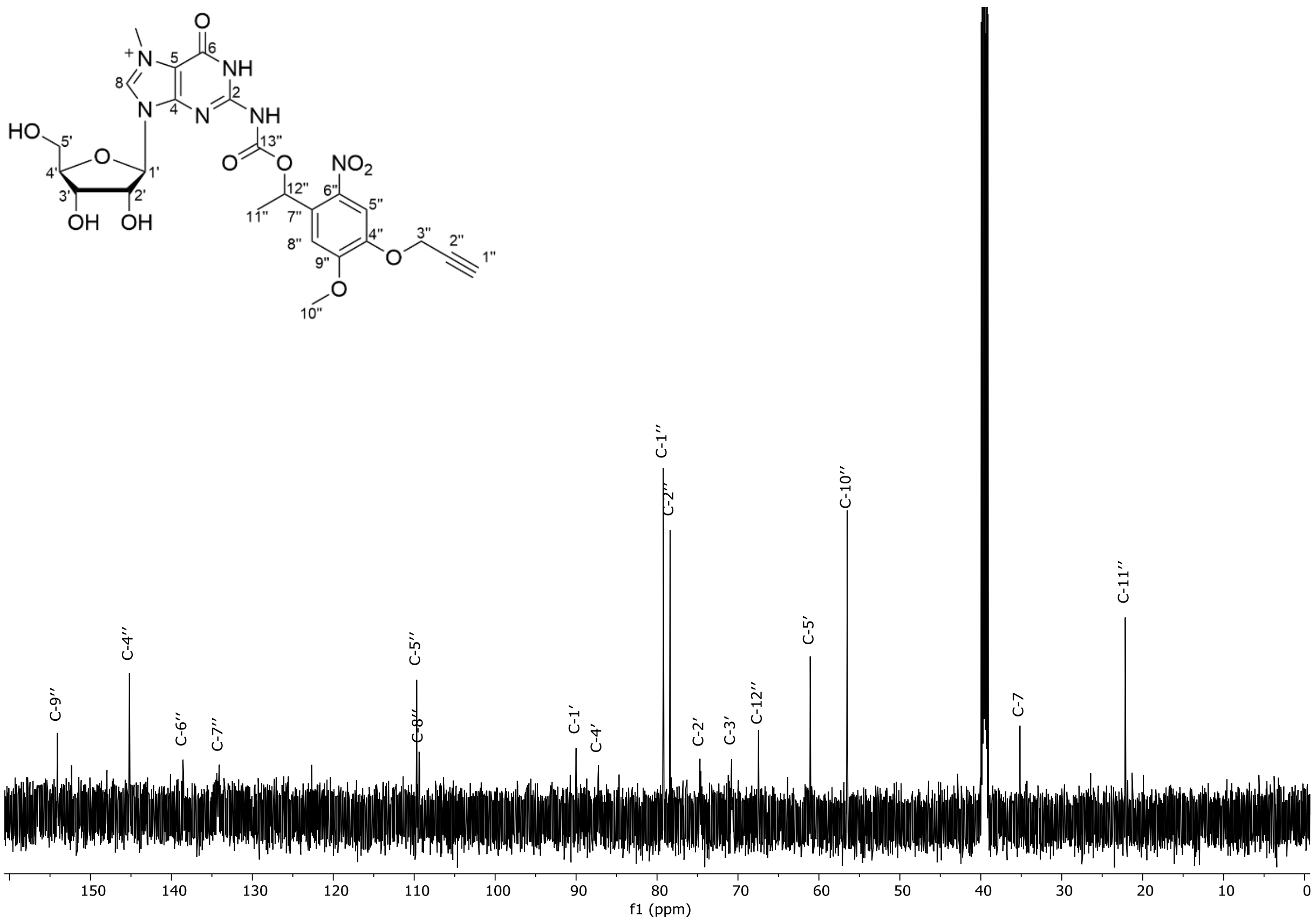

Figure S39 ^{13}C-NMR spectrum of compound **8**.

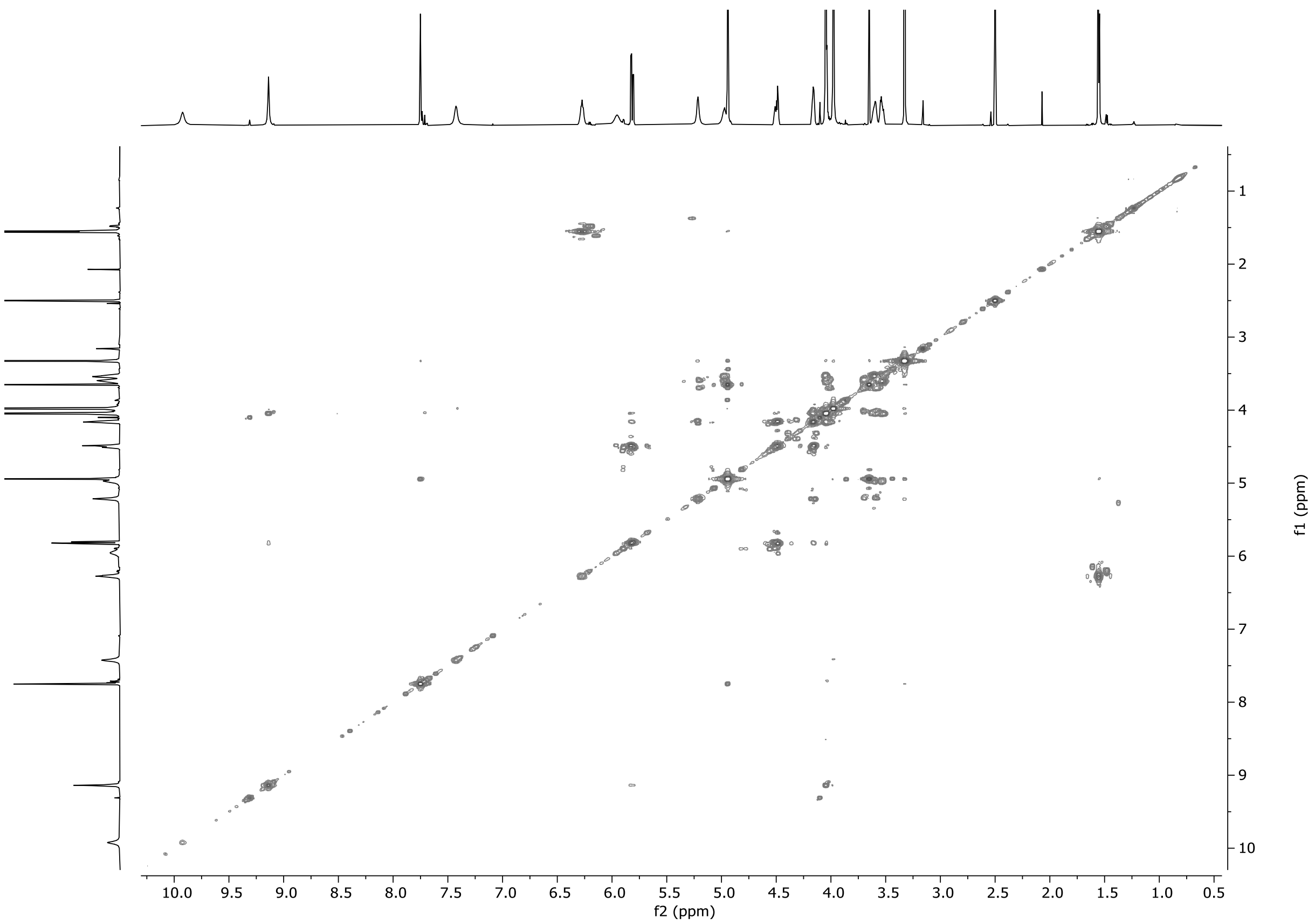

Figure S40 ^{1}H-^{1}H COSY spectrum of compound **8**.

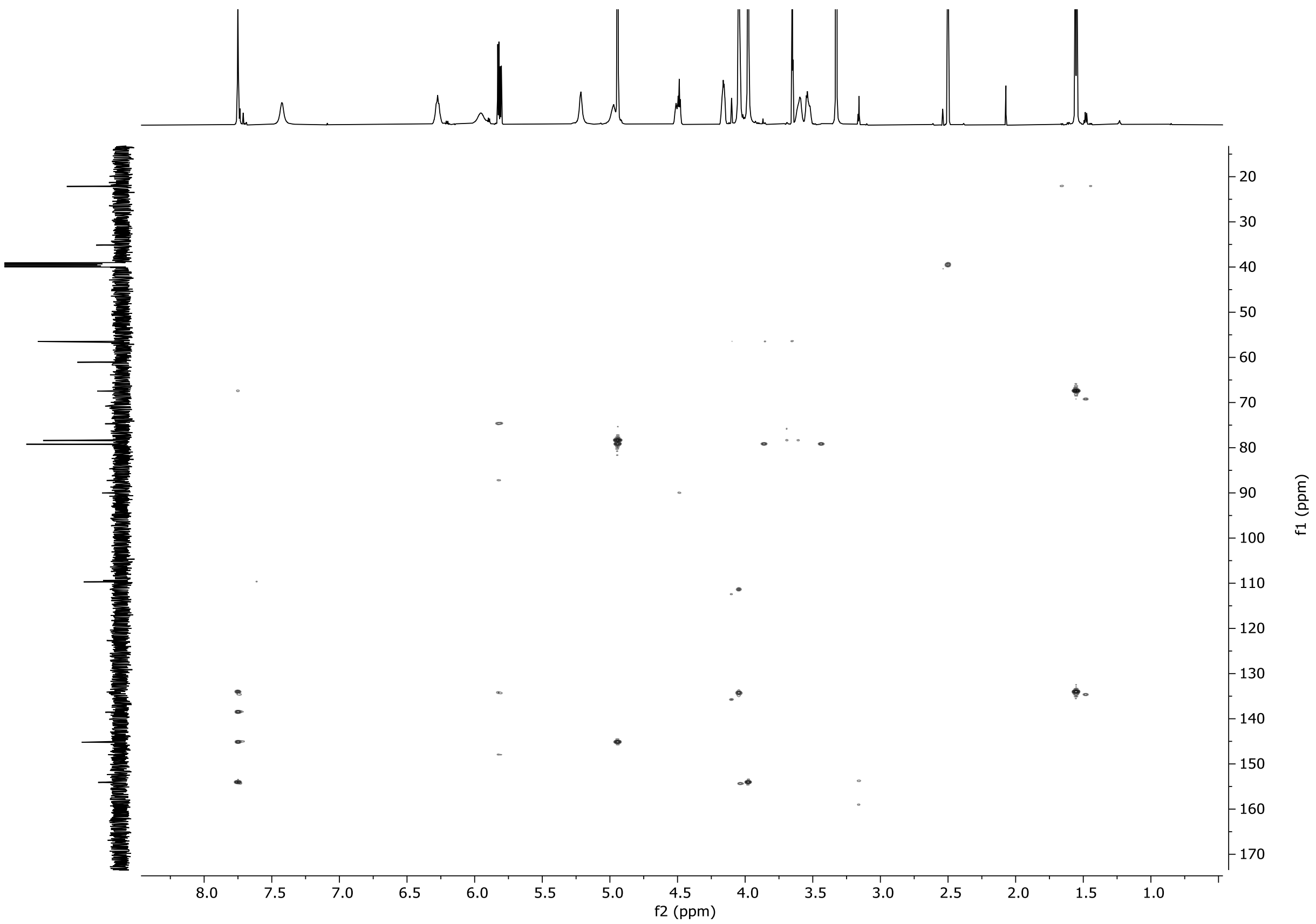

Figure S41 ^{1}H-^{13}C HMBC spectrum of compound **8**.

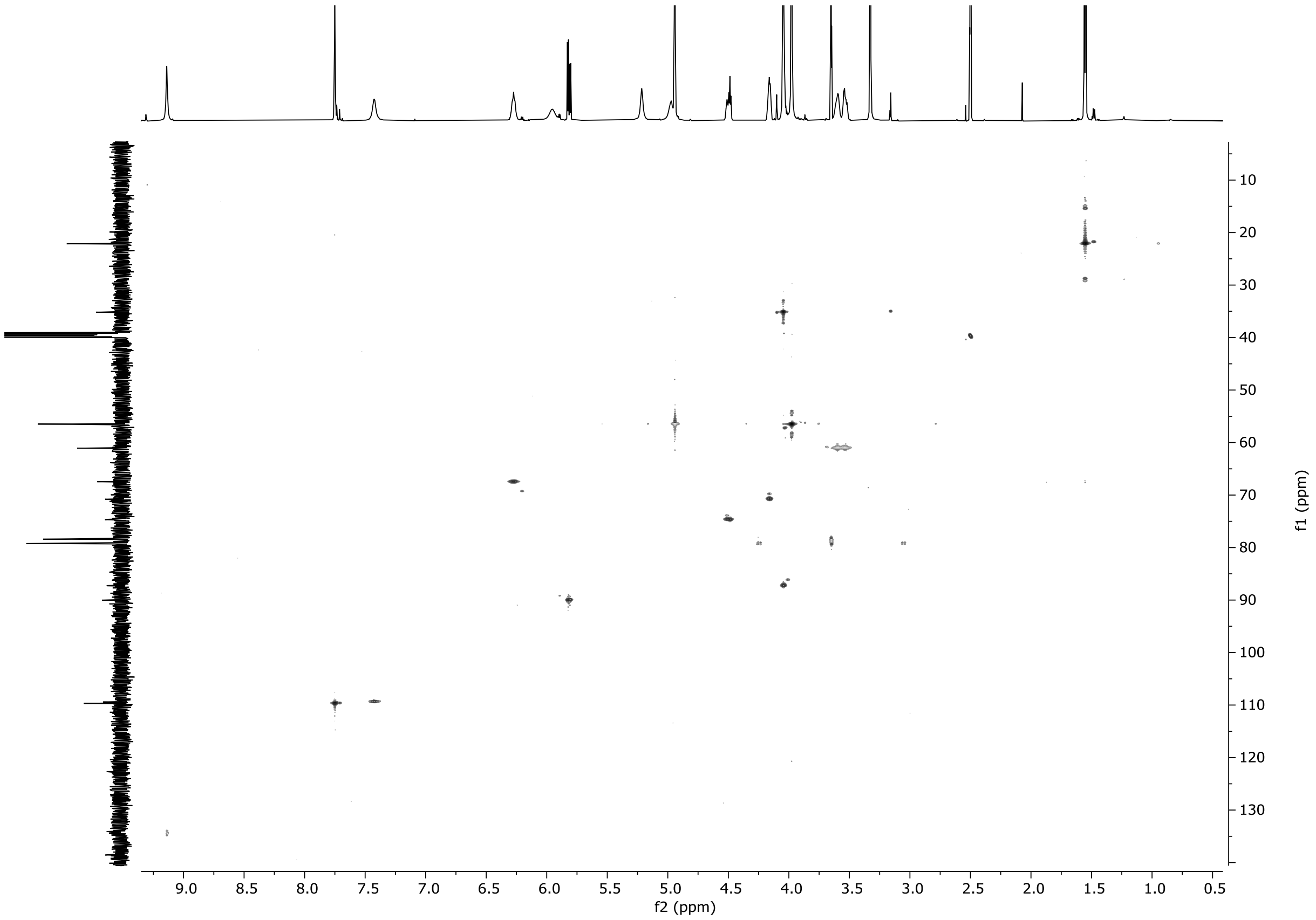

Figure S42 ^{1}H-^{13}C HSQC spectrum of compound **8**.

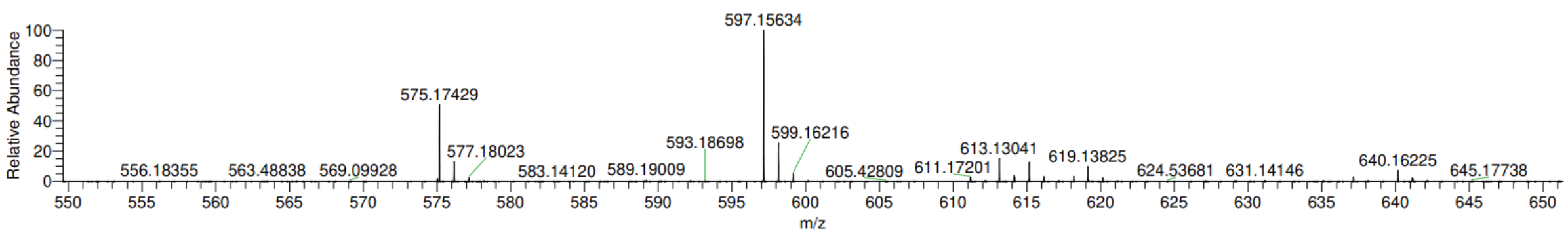

Figure S43 HRMS (m/z) of compound **8**: M^{+} calculated for $C_{24}H_{26}N_{6}O_{11}$ 575.17323, found 575.17429.

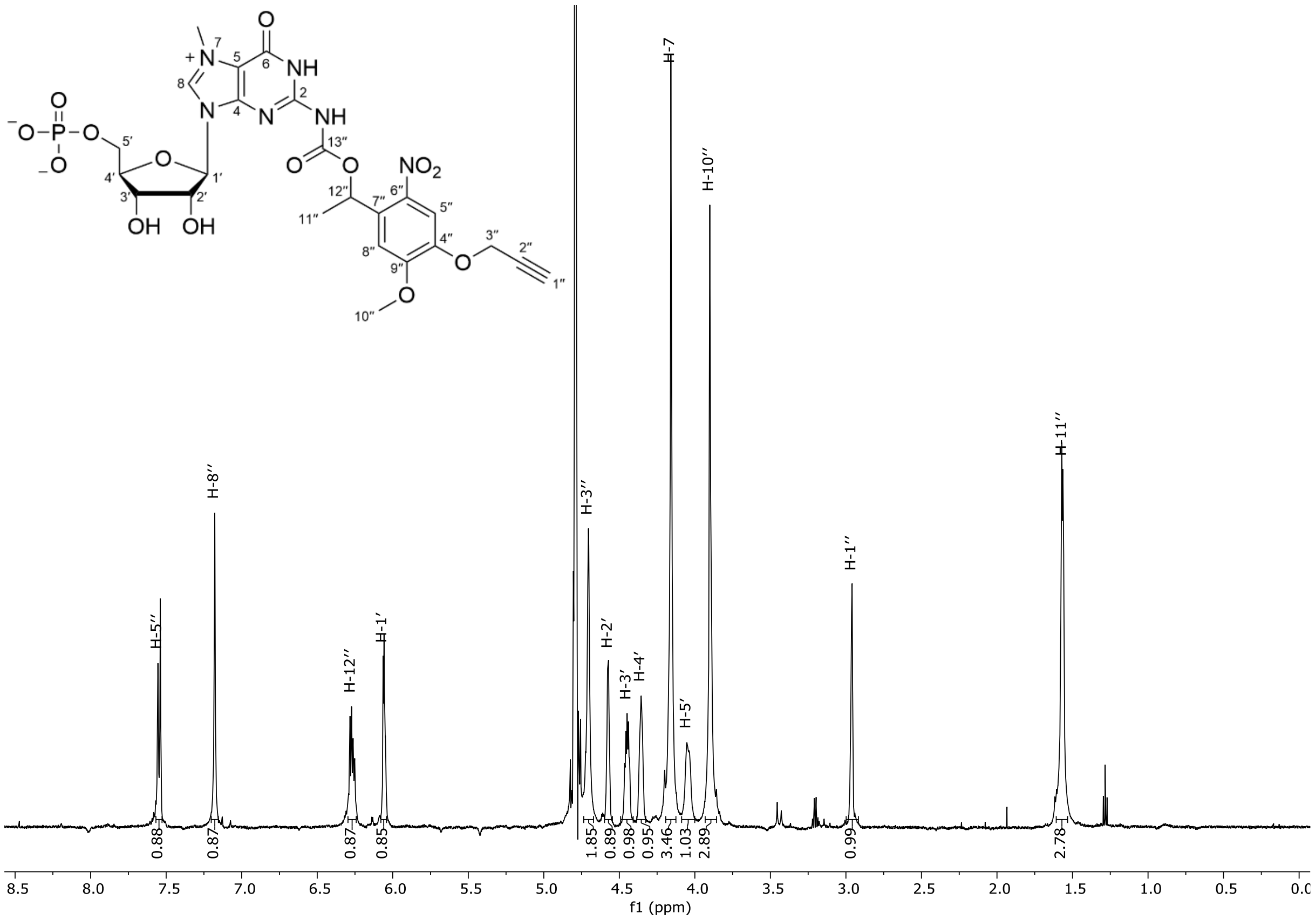

Figure S44 ^{1}H-NMR spectrum of compound **9**.

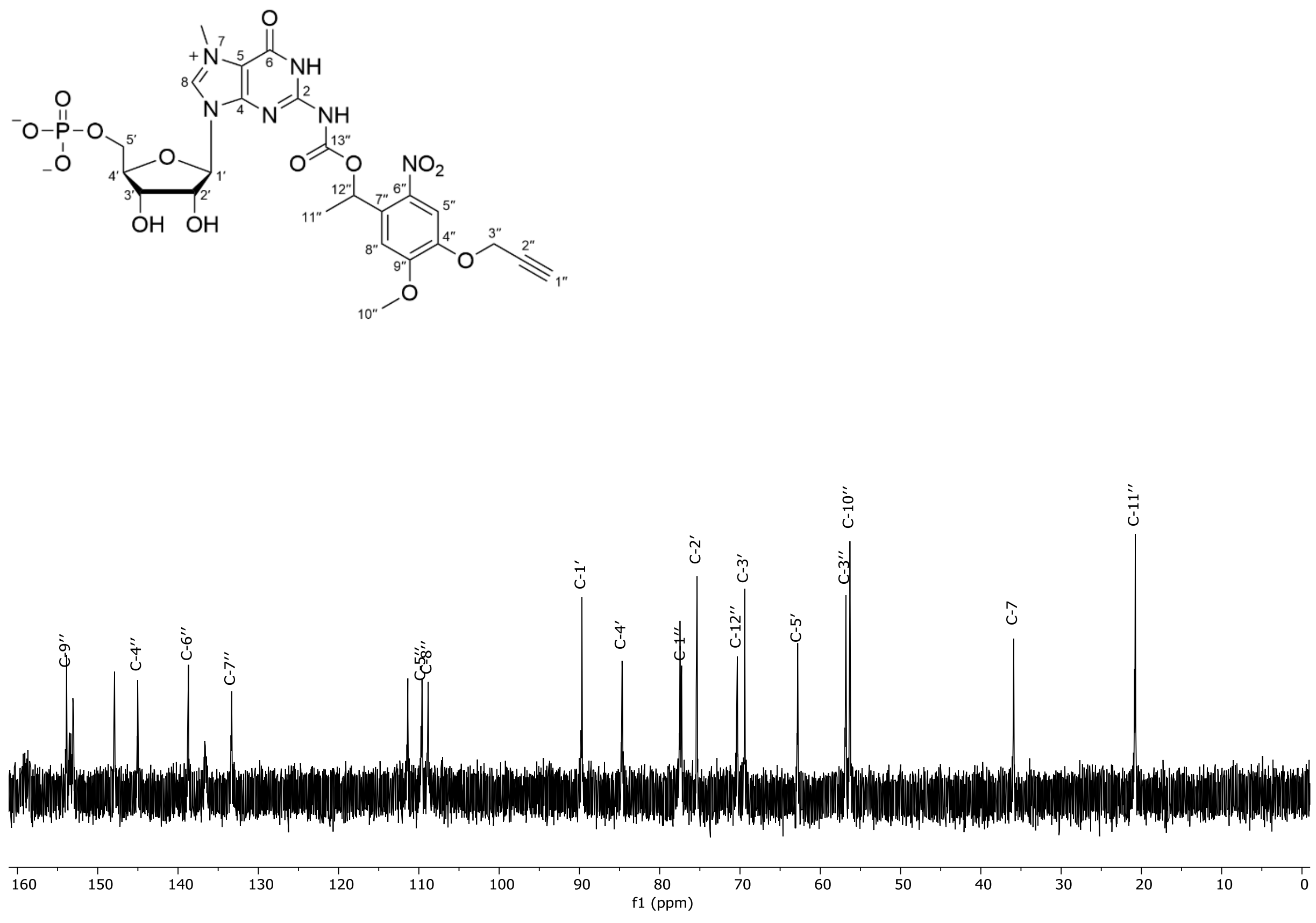

Figure S45 ^{13}C-NMR spectrum of compound **9**.

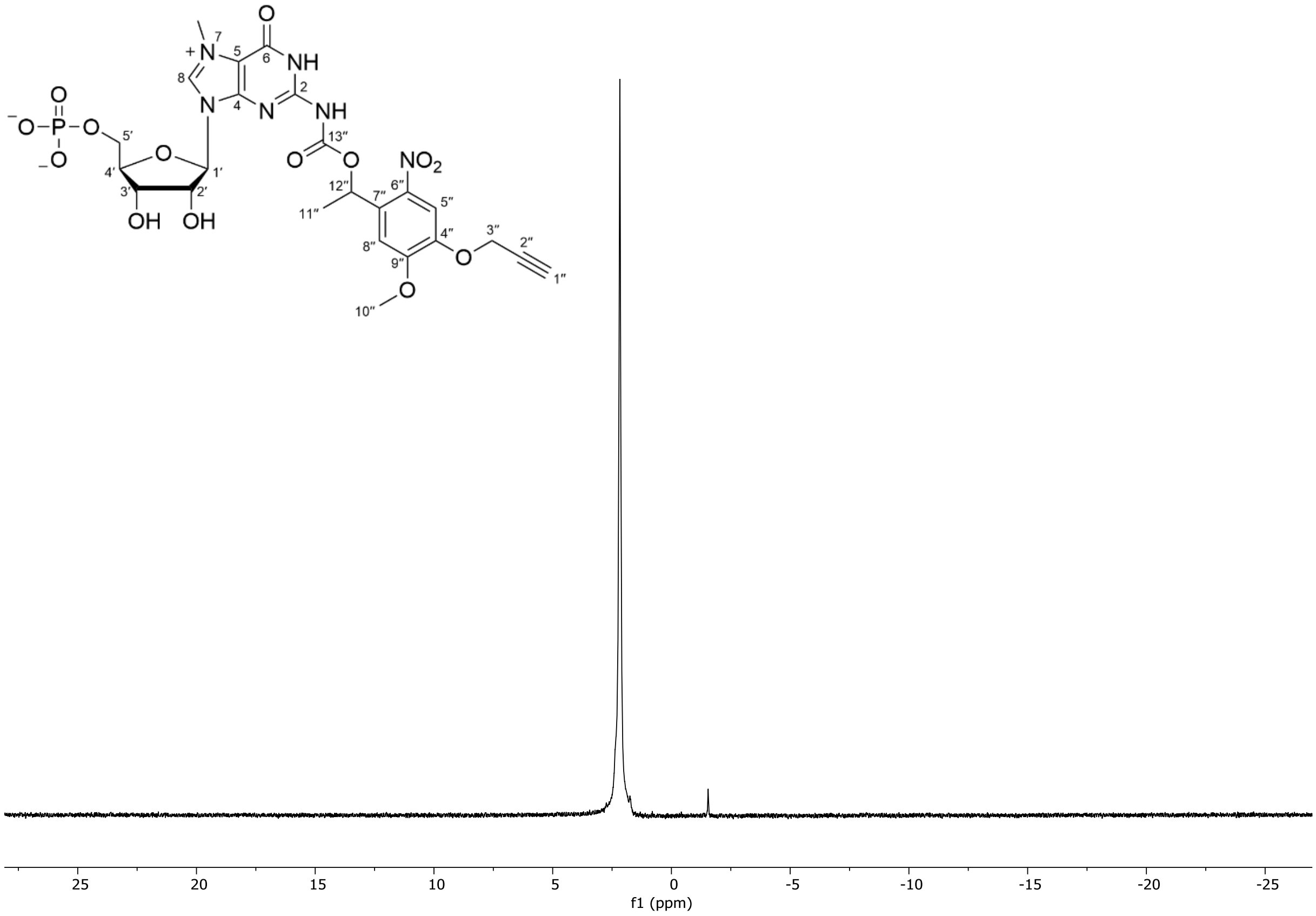

Figure S46 ^{31}P-NMR spectrum of compound **9**.

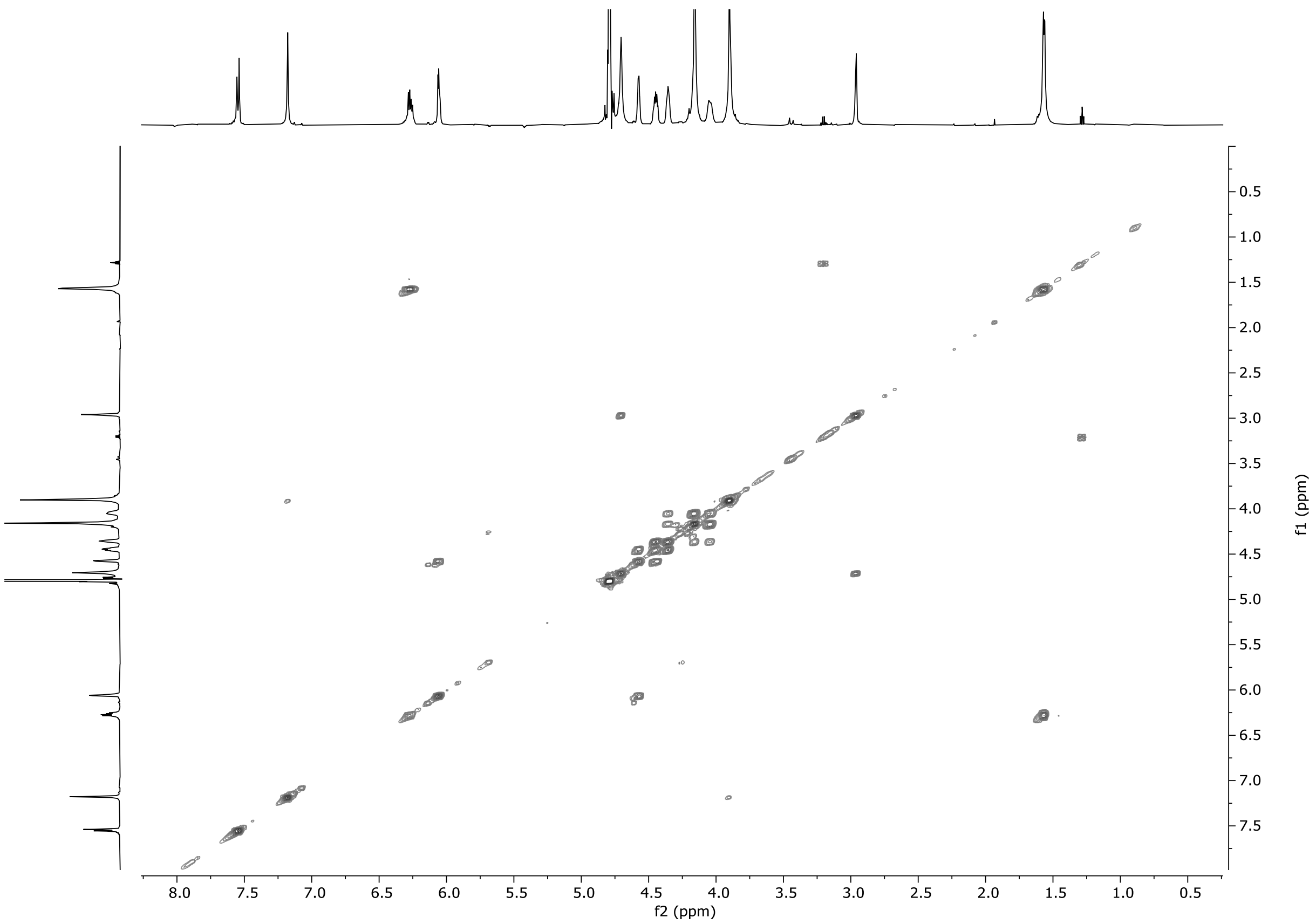

Figure S47 ^{1}H-^{1}H COSY spectrum of compound **9**.

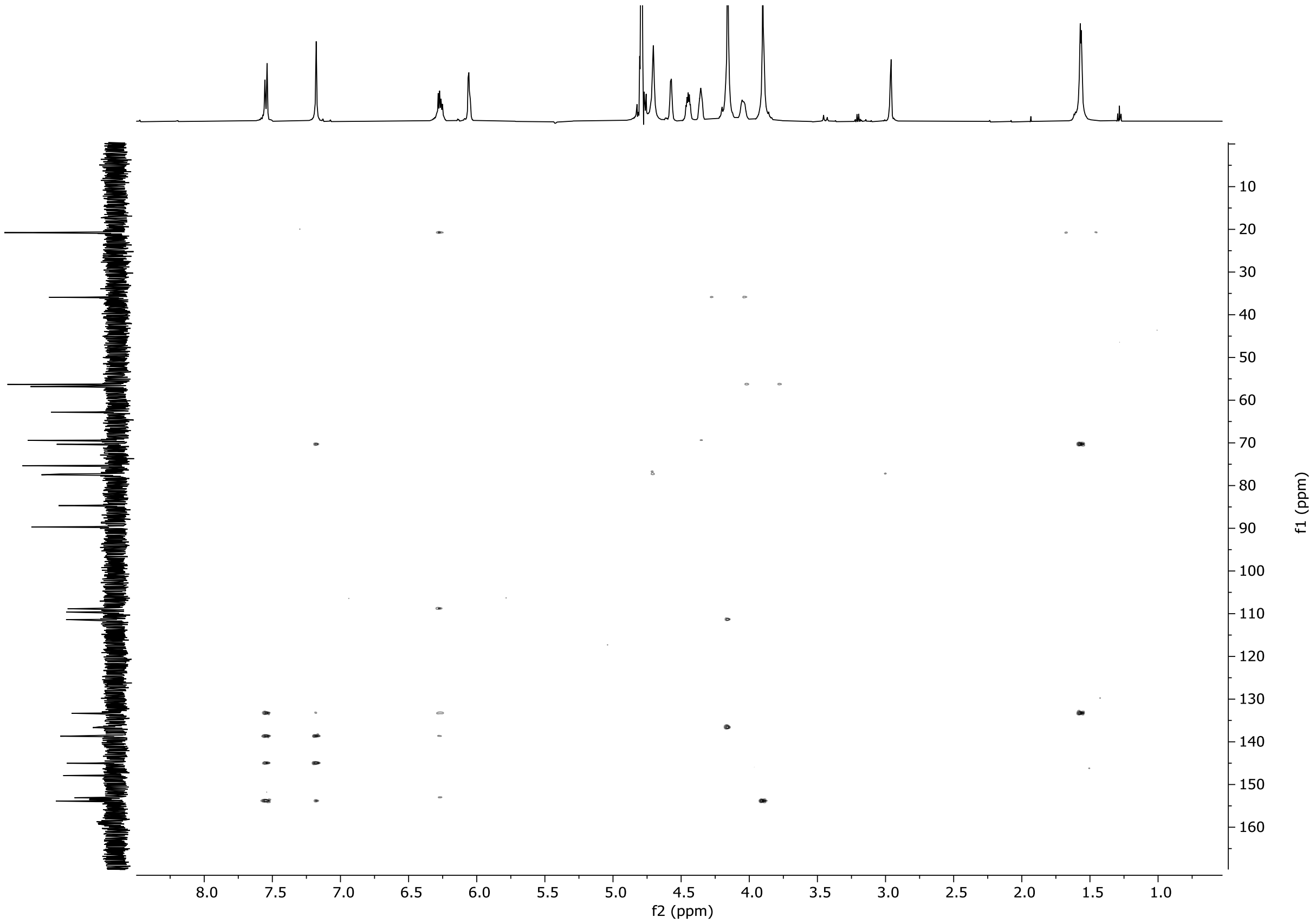

Figure S48 ^{1}H-^{13}C HMBC spectrum of compound **9**.

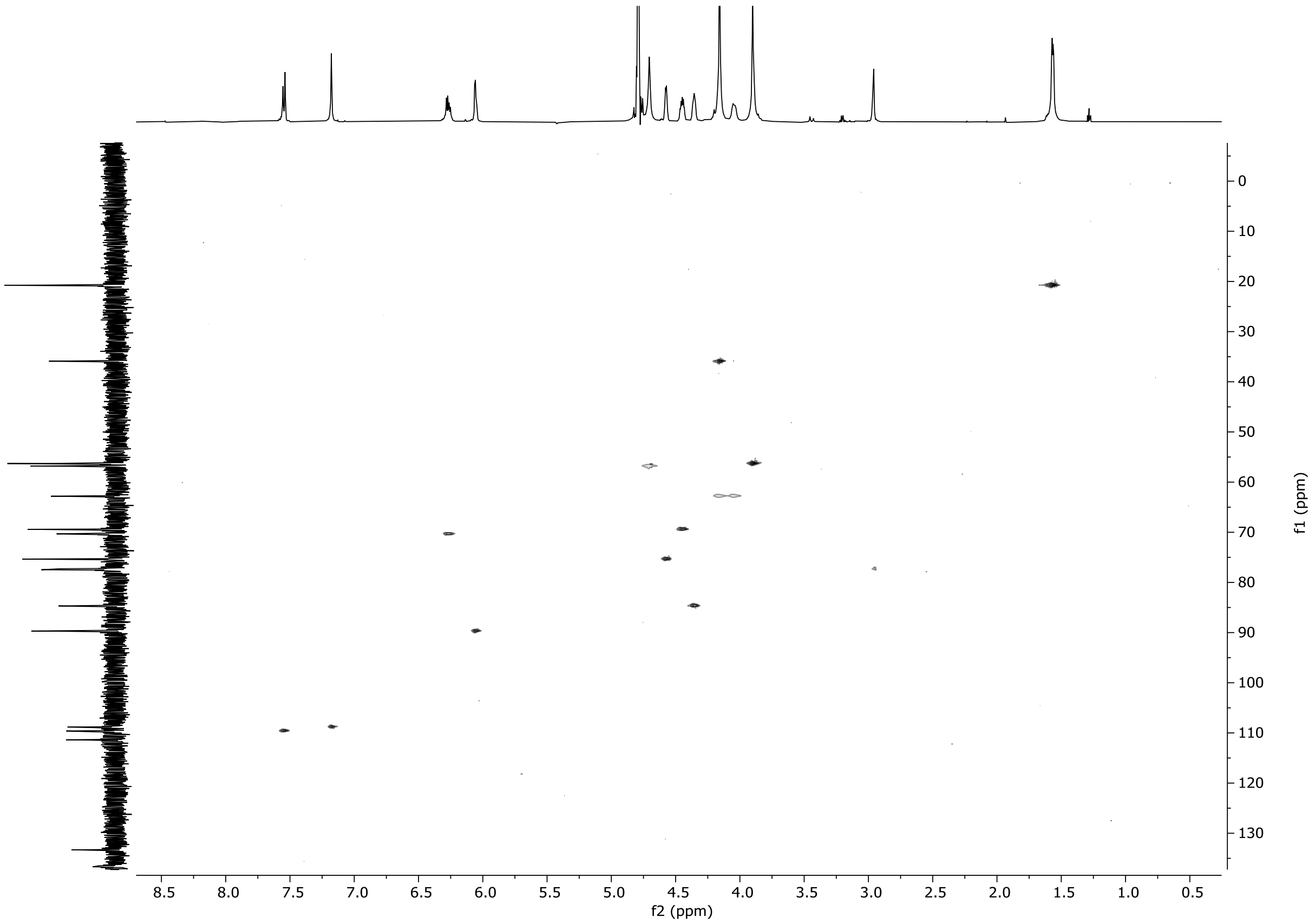

Figure S49 ^{1}H-^{13}C HSQC spectrum of compound **9**.

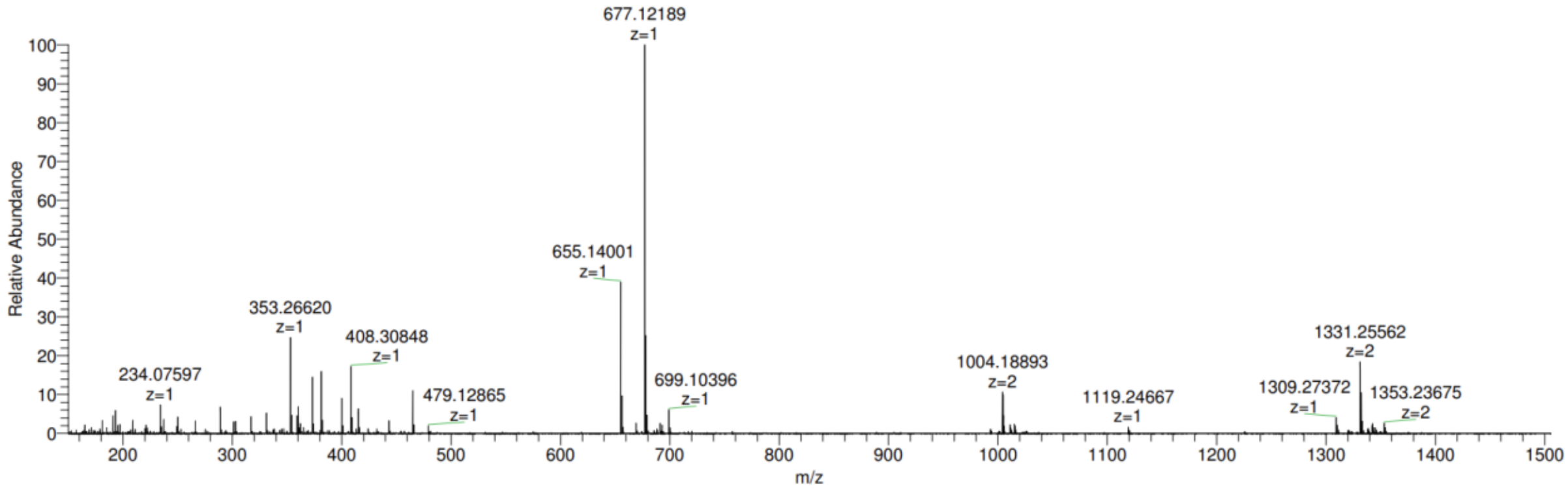

Figure S50 HRMS (m/z) of compound **9**: M+ calculated for $C_{24}H_{28}N_6O_{14}P$ 655.13959, found 655.14001.

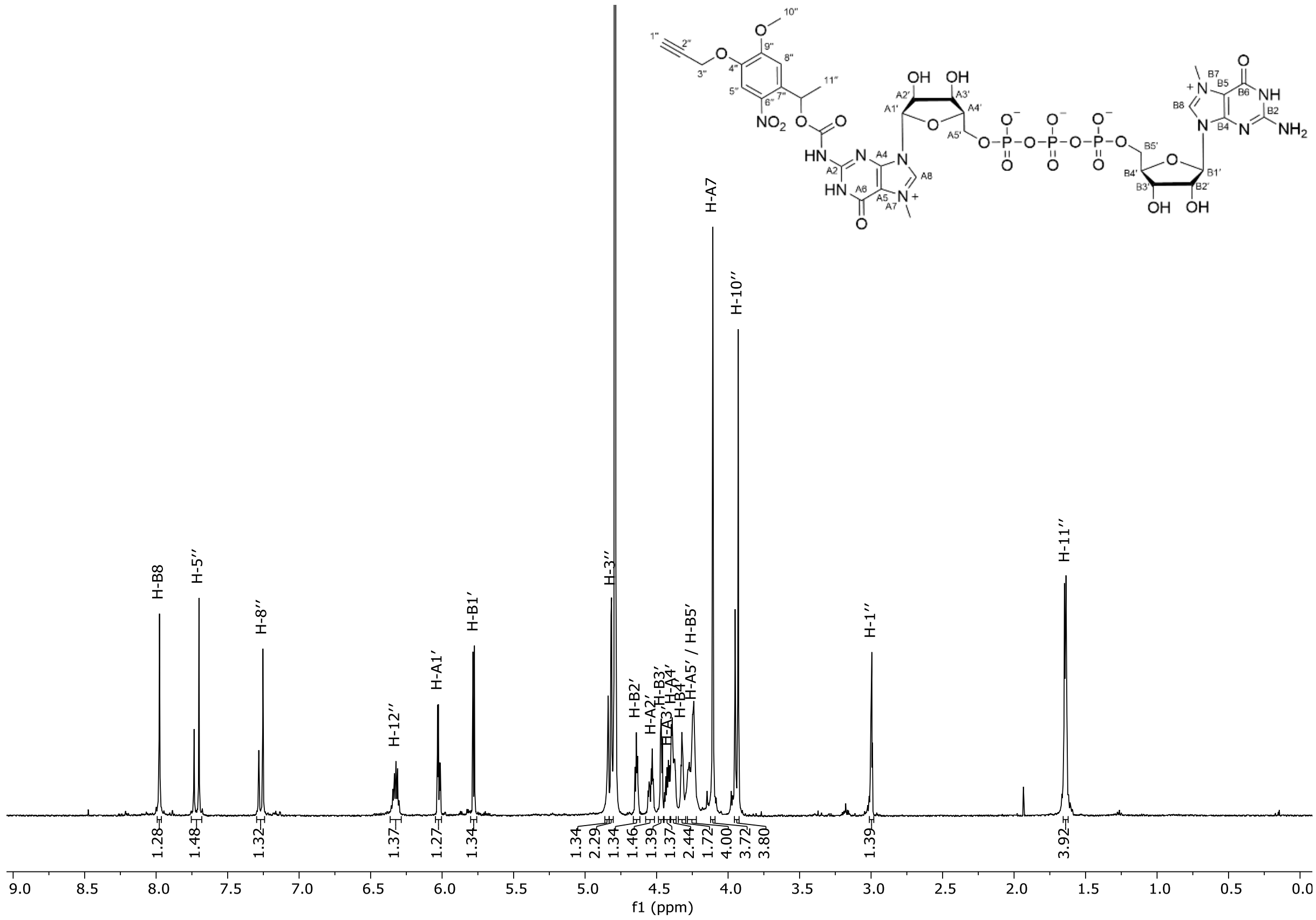

Figure S51 ^{1}H-NMR spectrum of compound **1**.

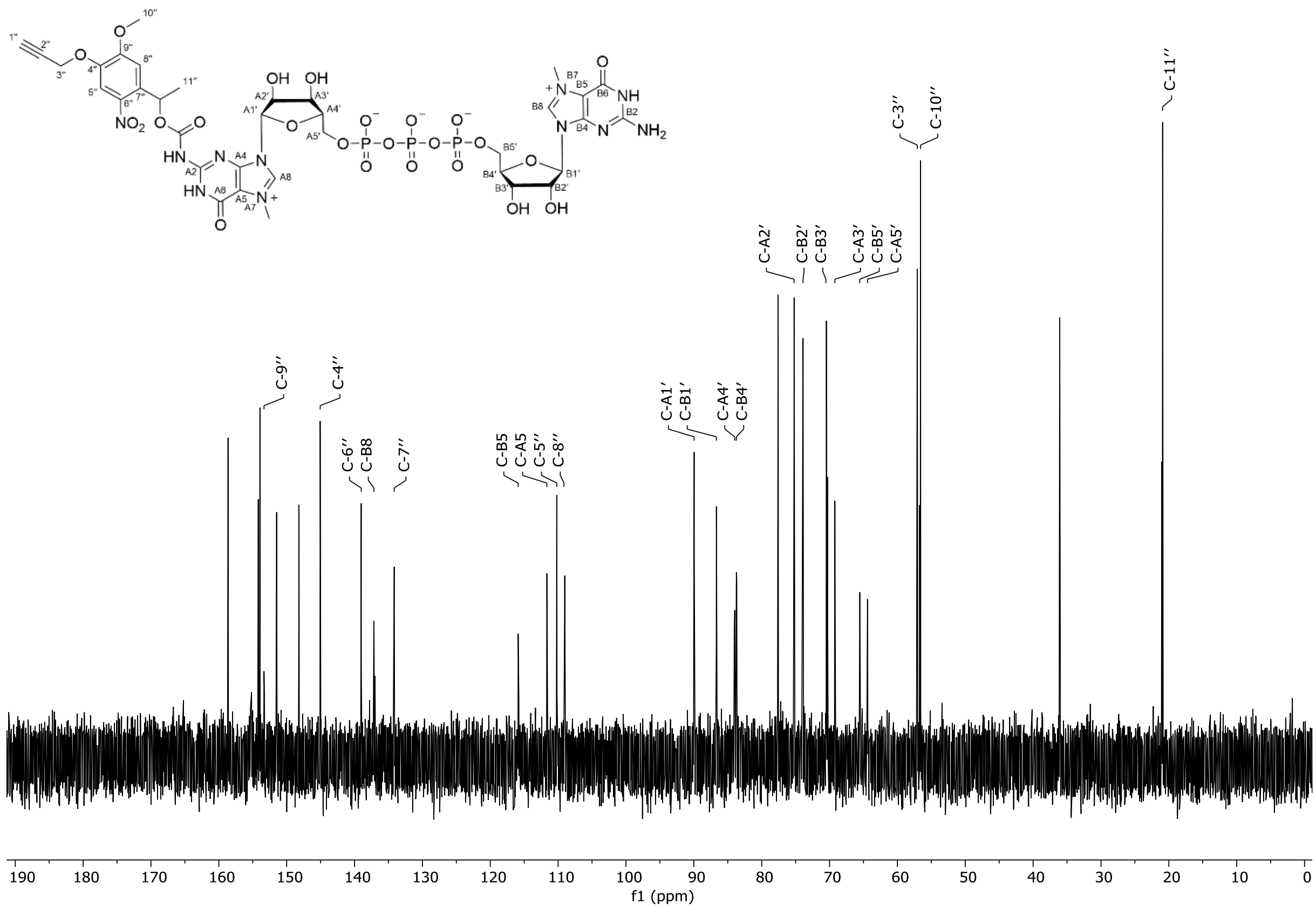

Figure S52 ^{13}C-NMR spectrum of compound **1**.

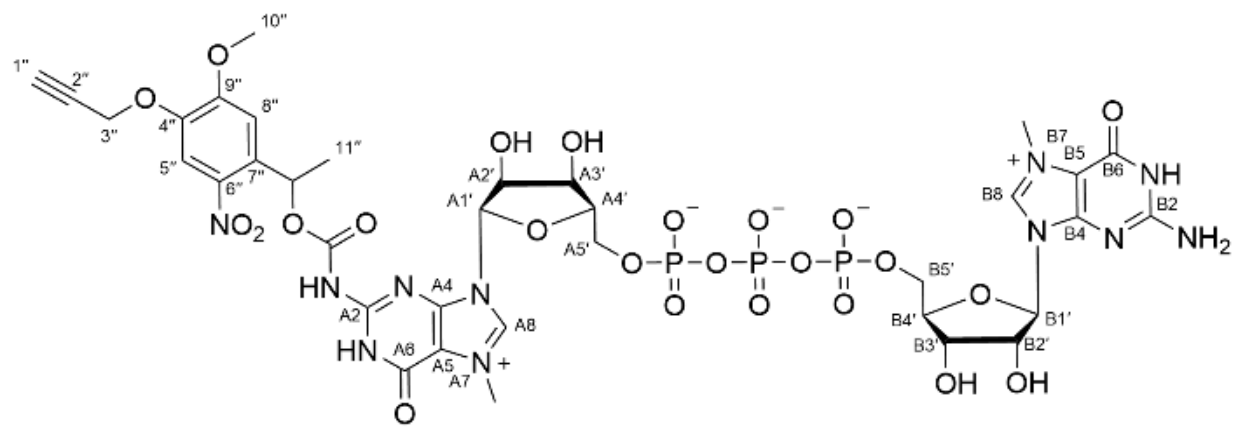

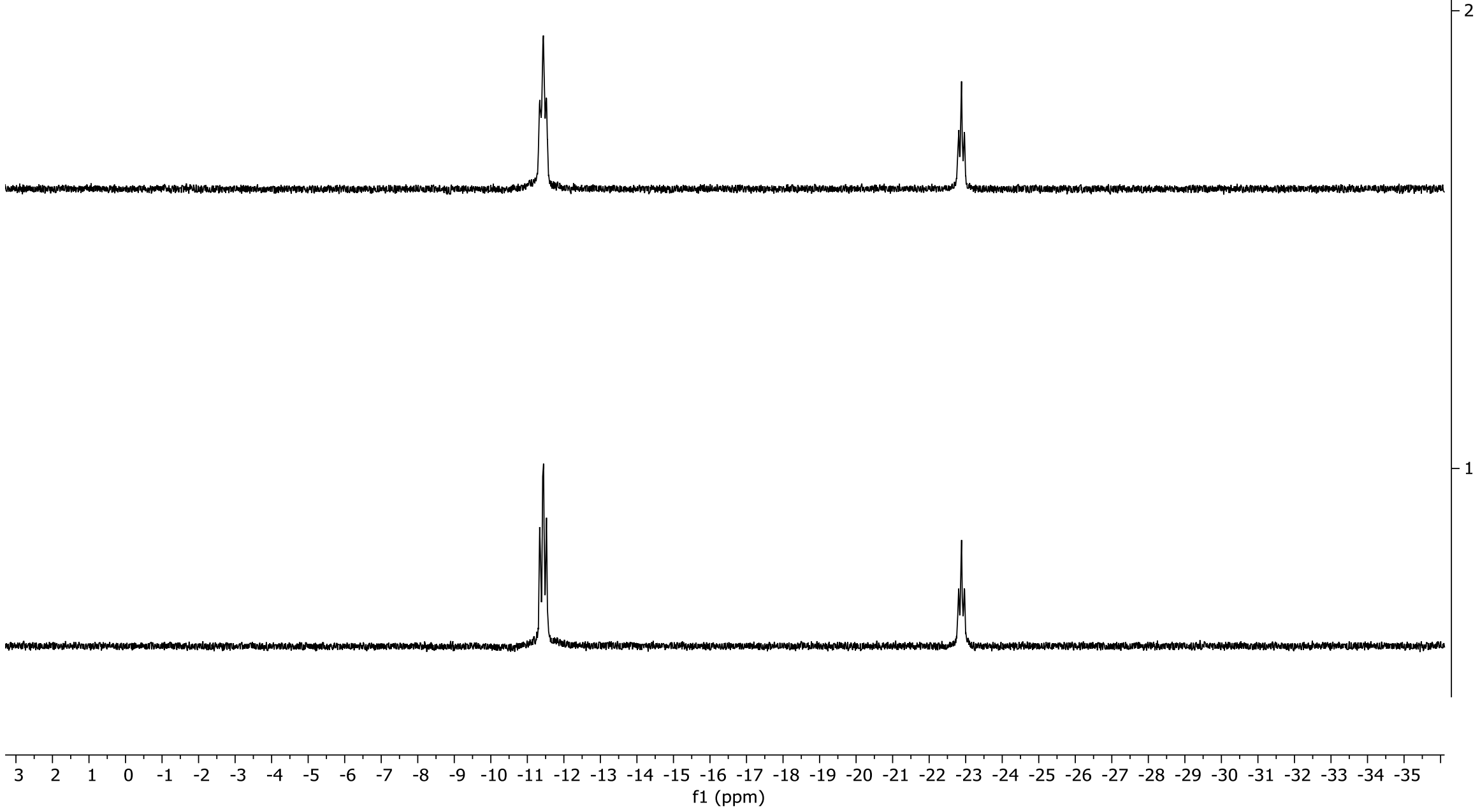

Figure S53 ^{31}P-NMR spectrum of compound **1**.

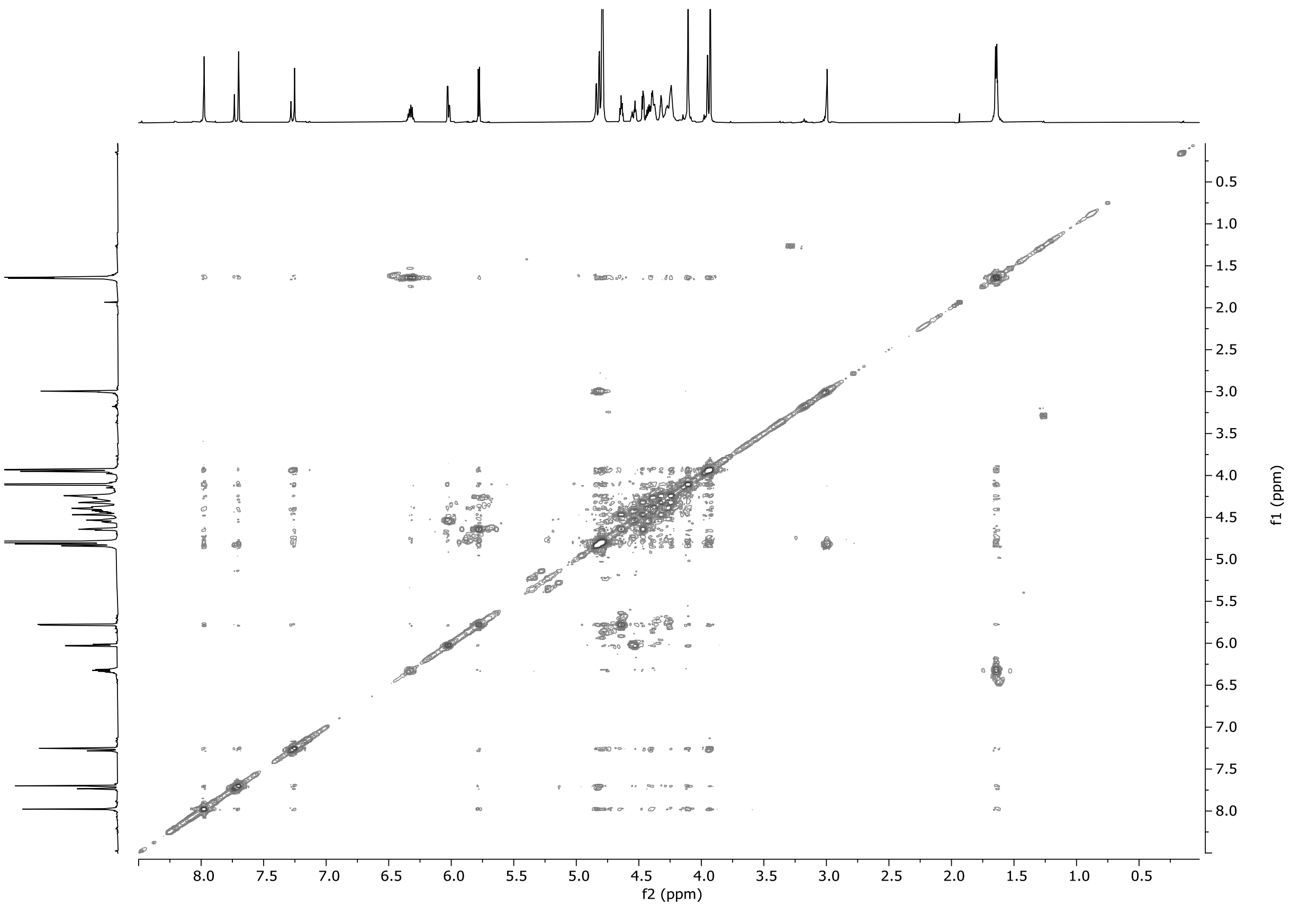

Figure S54 ^{1}H-^{1}H COSY spectrum of compound **1**.

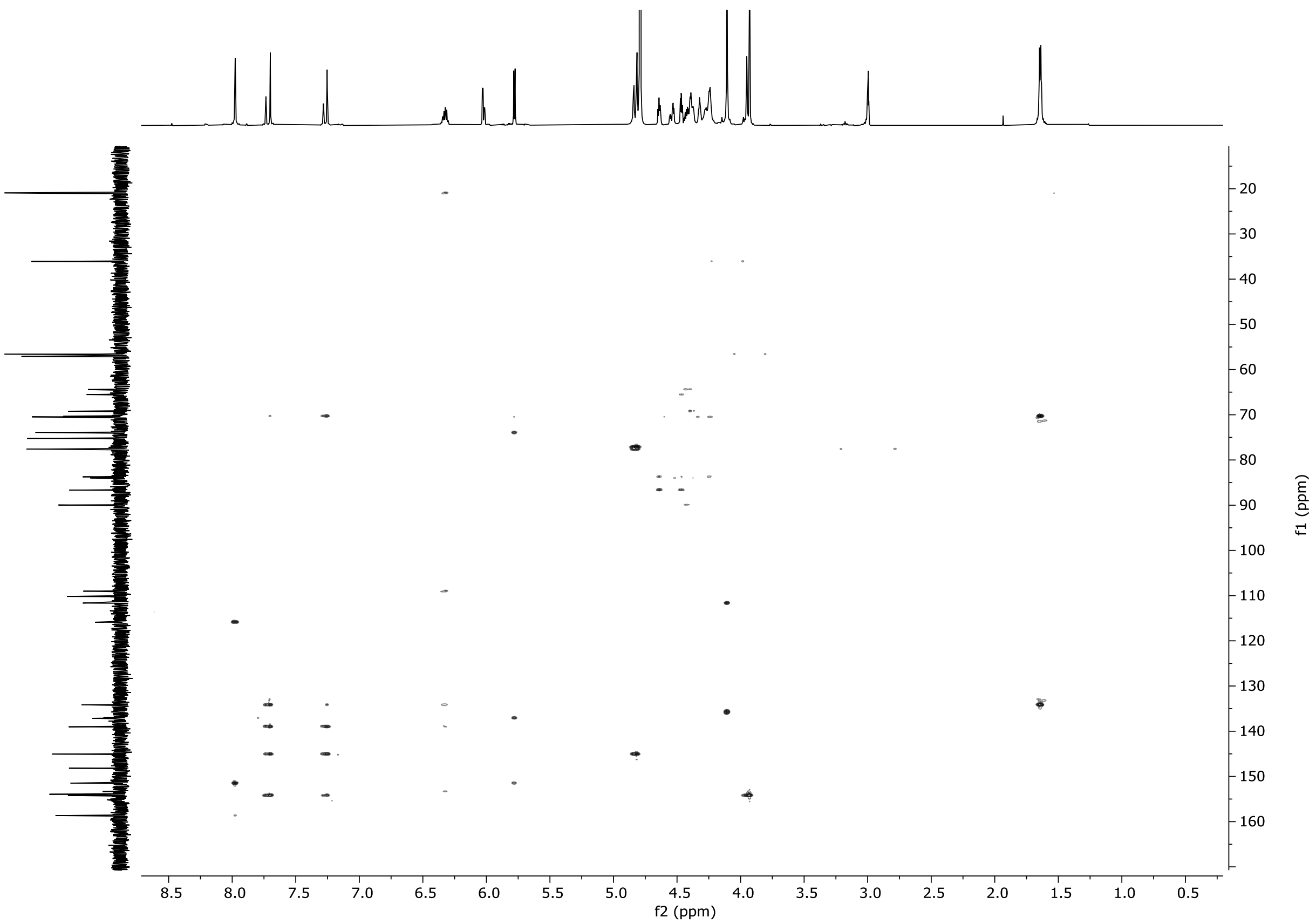

Figure S55 ^{1}H-^{13}C HMBC spectrum of compound **1**.

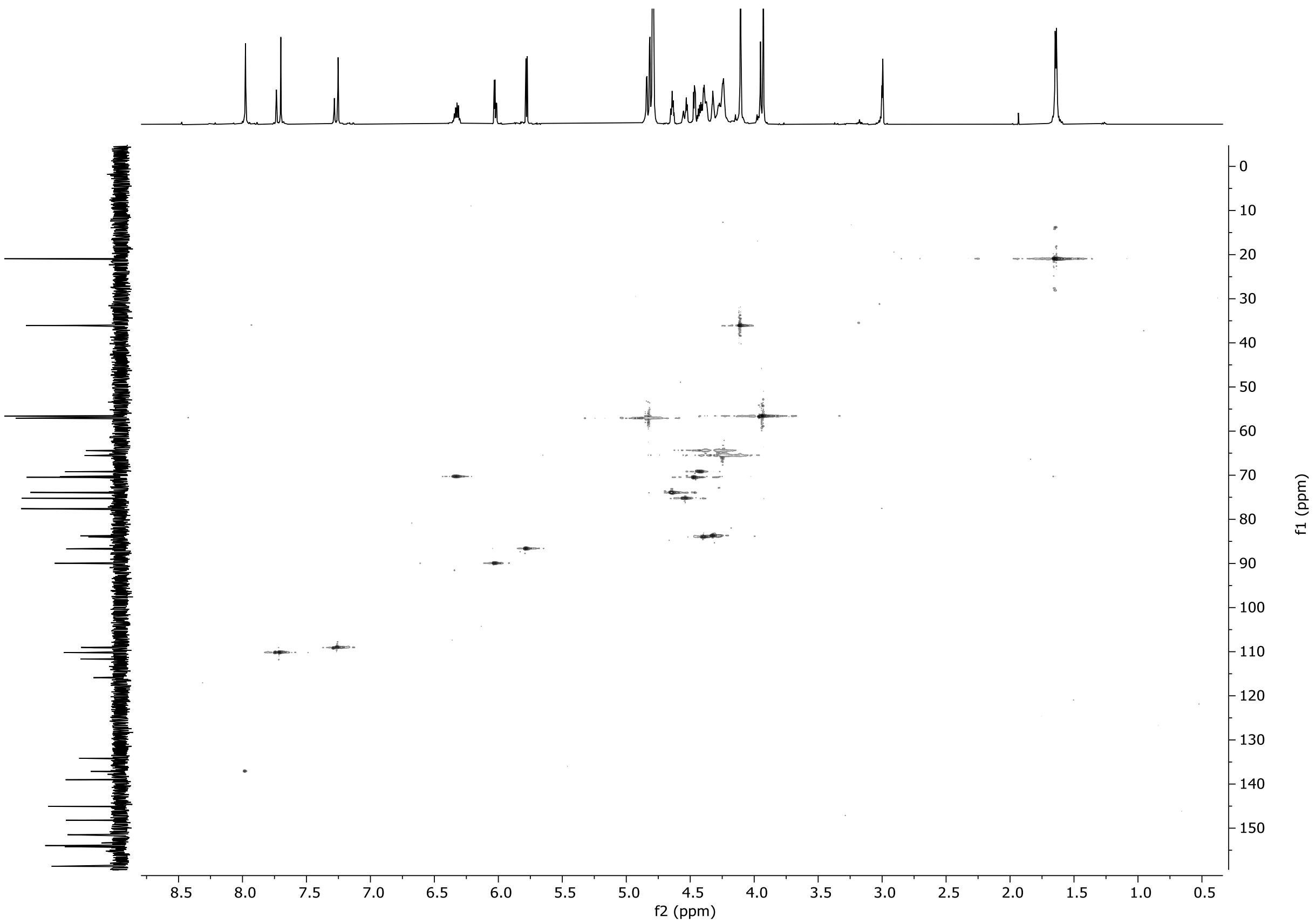

Figure S56 ^{1}H-^{13}C HSQC spectrum of compound **1**.

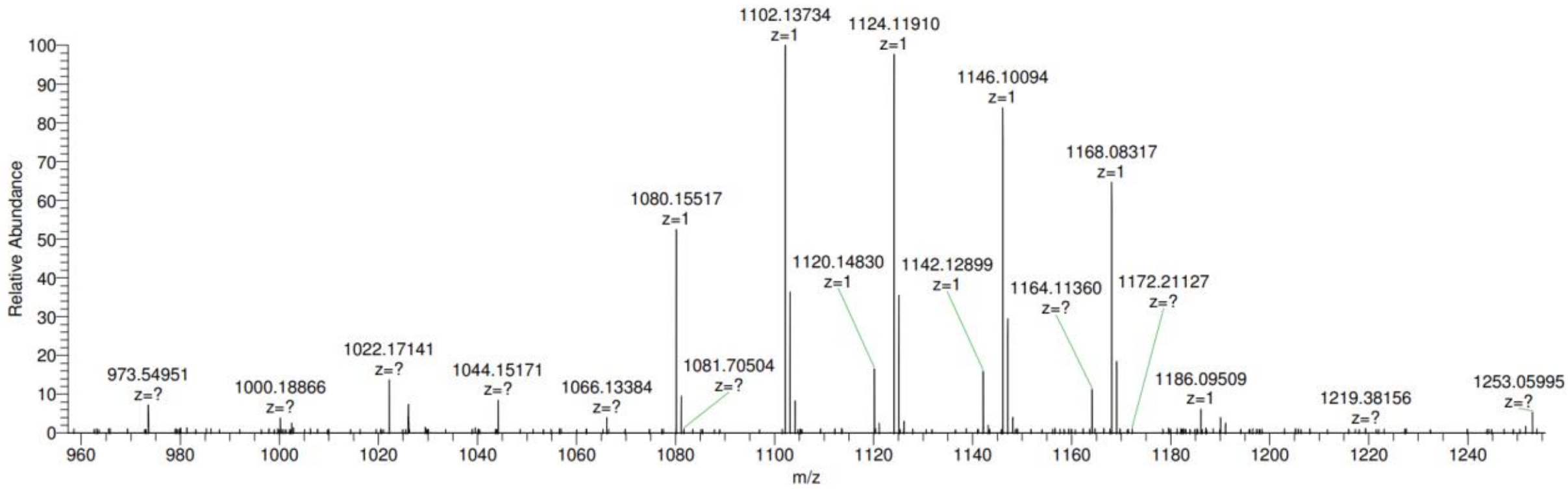

Figure S57 HRMS (m/z) of compound **1**: M⁺ calculated for $C_{34}H_{41}N_{11}O_{24}P_3$ 1080.15333, found 1080.15517.

6. References

[42] S. J. Teague, "Facile synthesis of a o-nitrobenzyl photolabile linker for combinatorial chemistry" *Tetrahedron Letters* **37**, (1996): 5751.

[43] J. Jemielity, T. Fowler, J. Zuberek, J. Stepinski, M. Lewdorowicz, A. Niedzwiecka, R. Stolarski, E. Darzynkiewicz, R. E. Rhoads, "Novel "anti-reverse" cap analogs with superior translational properties" *RNA (New York, N.Y.)* **9**, (2003): 1108.
